# Three-Component Synthesis of Pyridylacetic Acid Derivatives by Arylation/Decarboxylative Substitution of Meldrum’s Acids

**DOI:** 10.1021/acs.joc.2c01597

**Published:** 2022-10-18

**Authors:** Tarn C. Johnson, Stephen P. Marsden

**Affiliations:** School of Chemistry and Institute of Process Research and Development, University of Leeds, Leeds LS2 9JT, U.K.

## Abstract

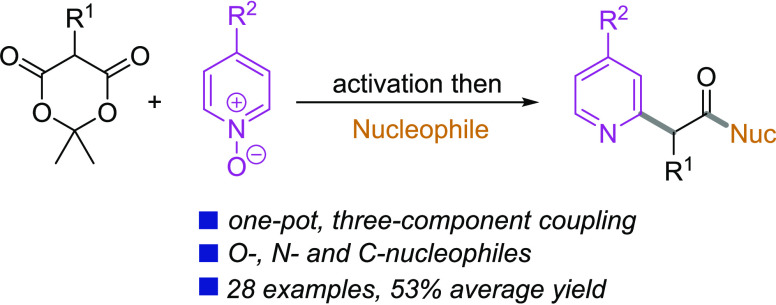

A convenient and simple three-component synthesis of substituted pyridylacetic acid derivatives is reported. The approach centers on the dual reactivity of Meldrum’s acid derivatives, initially as nucleophiles to perform substitution on activated pyridine-*N*-oxides, then as electrophiles with a range of nucleophiles to trigger ring-opening and decarboxylation.

## 1 Introduction

**Figure 1:**
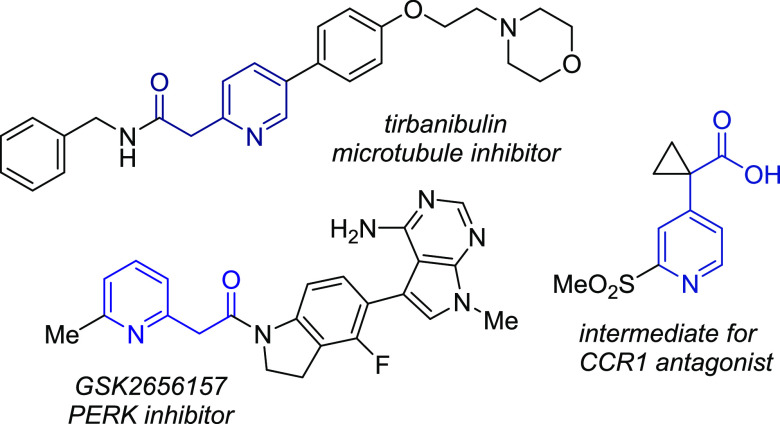
Representative pyridylacetic acid derivatives and proposed synthetic approach.

Pyridines are the most prevalent heterocyclic structures found in pharmaceutical products,^1^ among which pyridylacetic acid derivatives find use both as subunits of drugs and drug candidates and also as intermediates for their synthesis (Figure 1).^2^ Synthetic routes to substituted pyridylacetate derivatives (and their benzo-fused (iso)quinoline analogues) frequently start from halopyridines under conditions of palladium-catalyzed cross-coupling with lithium enolates,^2a,3^ silyl enol ethers,^4^ or Reformatsky reagents.^5^ Alternatively, metal-catalyzed^3,6^ coupling or direct S_N_Ar reactions^7^ of halopyridines and (iso)quinolines can be carried out with activated methylene compounds such as malonates,^6a,6b,7a^ ketoesters,^6d^ cyanoacetate,^3,6c^ or Meldrum’s/barbituric acids,^7b^ followed by hydrolysis/decarboxylation or deacylation.

Precious metal-free direct S_N_Ar reactions can also be carried out with metallated alkylnitrile nucleophiles, followed by hydrolysis.^2b,2c^ A consideration with all such approaches is that 2- or 4-pyridylacetic acids are themselves prone to ready decarboxylation,^7a^ so care is needed in the choice of conditions.

An alternative approach to the substitution of halopyridines is to employ pyridine-*N*-oxides (or benzo-fused variants) in conjunction with an electrophilic activating agent. In this way, a similarly broad range of active methylene nucleophiles may be heteroarylated,^8^ along with alternative nucleophiles such as silyl ketene acetals^9,10^ and aldehydes (under conditions of enamine organocatalysis).^11^ We have previously reported the use of azlactones as nucleophiles for the substitution of activated pyridine-*N*-oxides.^12^ The intermediate azlactones served as electrophiles that could be opened with a diverse range of nucleophiles (alcohols, amines, organometallics, and hydride reagents) to give α,α-disubstituted amino acid derivatives;^12a^ alternatively, the use of water as a nucleophile triggered a hydrolysis/decarboxylation sequence to achieve a formal “umpoled” synthesis of 2-(1-amidoalkyl)pyridines.^12b^

**Scheme 1:**
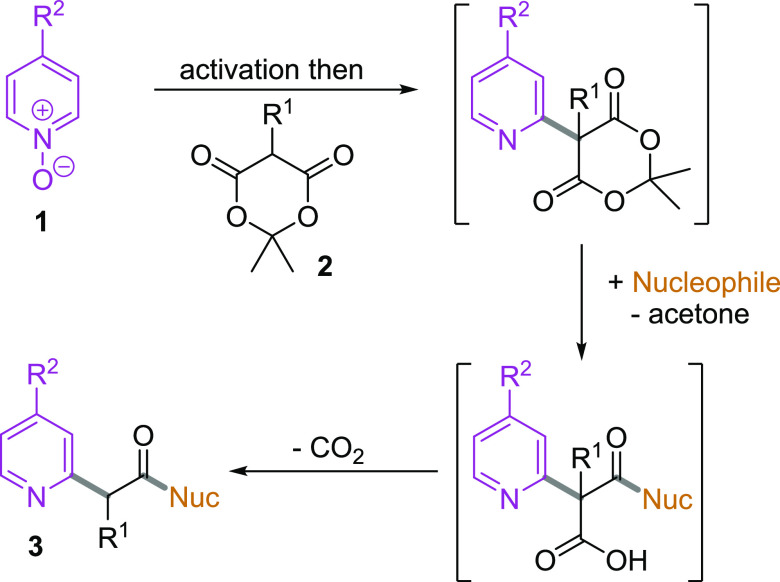
Proposed Synthetic Approach

We envisaged that a general synthesis of diverse pyridylcarboxylate derivatives would be possible using Meldrum’s acids in place of azlactones (Scheme 1). Thus, activation of pyridine-*N*-oxides **1** and nucleophilic substitution by the Meldrum’s acid derivatives **2** would generate an intermediate, which could act as an electrophilic partner for ring-opening by a range of nucleophiles; the resulting carboxylic acids would undergo facile decarboxylation to yield the desired product **3**. Below, we describe the successful implementation of this simple three-component approach to substituted pyridylacetic acid derivatives, along with an investigation into the substrate scope of the process.^13,14^

## 2 Results and Discussion

We began by investigating the coupling of 5-methyl Meldrum’s acid **2a** with pyridine-*N*-oxide **1a** under our previously developed activation conditions using tosyl chloride and triethylamine (Table 1). On completion of the substitution reaction, the solvent was swapped for methanol, and sodium methoxide was added. We were pleased to find that the desired pyridyl-substituted propionate ester **3a** was isolated in 63% yield as a single regioisomer, with substitution readily identified as occurring at the 4-position by analysis of the ^1^H and ^13^C NMR spectra.

**Table 1:**
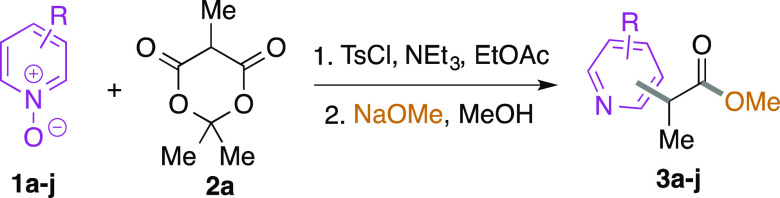

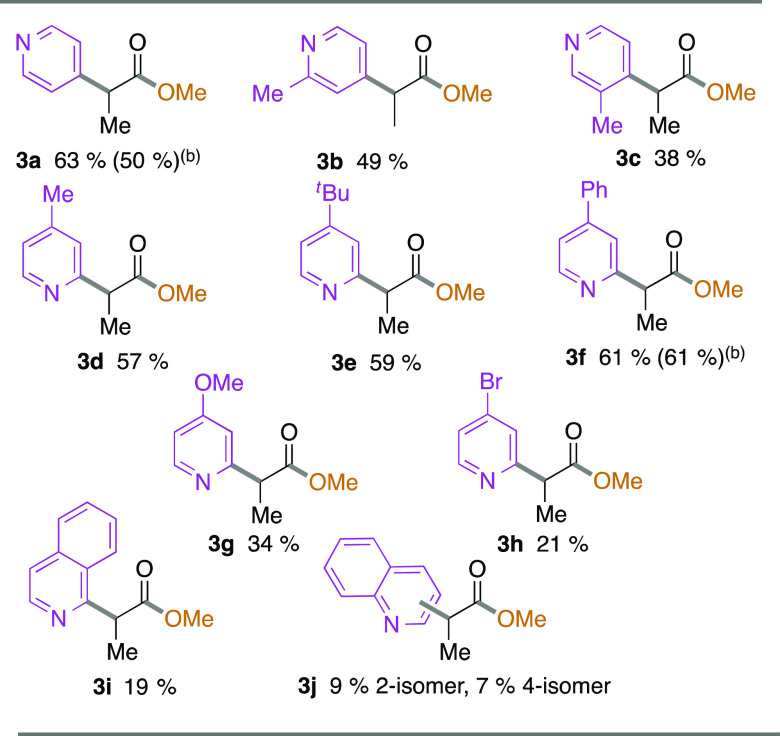
Three-Component Coupling: Scope of the Pyridine-*N*-oxide

a **1** (1.1 equiv), **2** (1.0 equiv), TsCl, (1.1 equiv), Et_3_N (2.1 equiv), EtOAc (0.2 M), r.t., overnight; remove solvent; then NaOMe (2.2 equiv), MeOH (2.5 M), r.t., 2–6 h.

b Yields for reactions on 1.25 mmol scale.

Notably, this regiochemical outcome is complementary to that reported in the addition of other activated methylene compounds to **1a** activated by Py-BroP, where clean 2-substitution is observed.^8d^ In that case, selectivity for the 2-position was rationalized based on charge association of the activated pyridine-*N*-oxide with the nucleophile. In our case, differences may arise because of the greater stabilization and hence lower nucleophilicity^15^ of the Meldrum’s acid-derived nucleophile, leading to a preference for addition to the 4-position consistent with the kinetic addition of soft nucleophiles to the 4-position of *N*-alkylpyridinium salts.^16^

We proceeded to examine the scope of the coupling of **2a** with various substituted pyridine-*N*-oxides **1** (Table 1). Substitution at the 4-position was also observed with 2- and 3-methylpyridine-*N*-oxides **1b**/**c** to give esters **3b**/**c**, while when 4-substituted pyridine-*N*-oxide substrates were employed, clean substitution at the 2-position was observed.

Alkyl (**3d**,**e**) and aryl (**3f**) substituents were well tolerated. The presence of an electron-donating alkoxy group was also tolerated, albeit that **3g** was formed in a slightly lower yield, as was bromo-substituted **3h**. Finally, isoquinoline-*N*-oxide and quinoline-*N*-oxide gave somewhat lower yields of the substitution products **3i**,**j**, the latter as an effectively equimolar mixture of regioisomers. Synthesis of compounds **3a** and **3f** was also repeated on a larger (1.25 mmol) scale, with comparable yields being observed.

Variation of the carboxylate side chain was investigated using substituted Meldrum’s acids **2b**–**i**, which were prepared using a modified one-pot reductive coupling of Meldrum’s acid itself with aldehydes, mediated by sodium triacetoxyborohydride (see the Supporting Information).^17^ The resulting nucleophiles were reacted with pyridine-*N*-oxide **1a** under the standard conditions and then subjected to methanolysis/decarboxylation (Table 2).

**Table 2:**
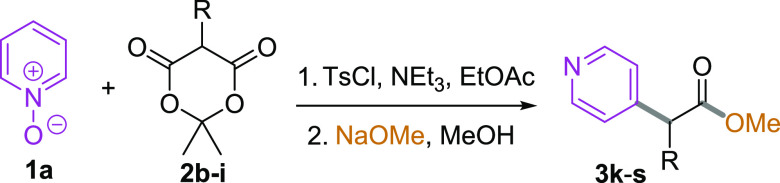

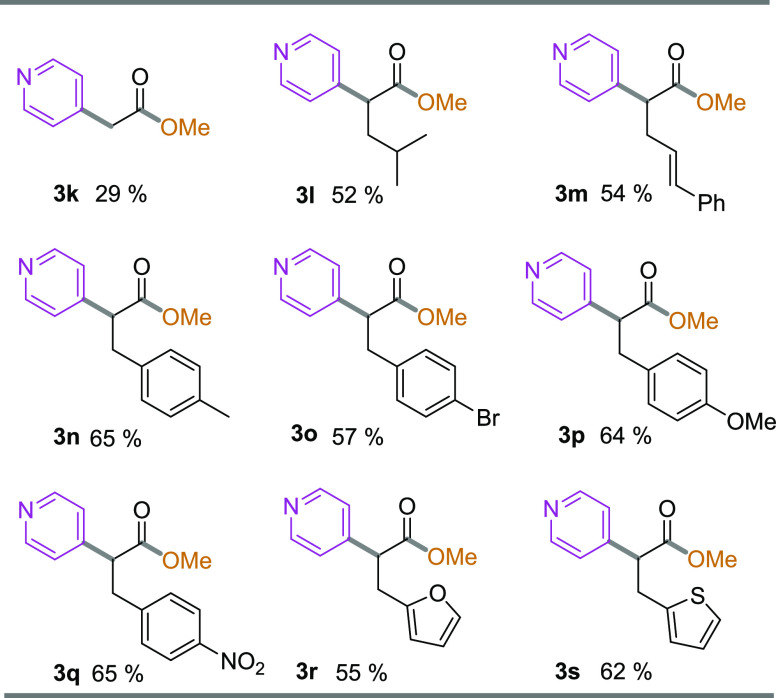
Three-Component Coupling: Scope of the Meldrum’s Acid^a^

a **1** (1.1 equiv), **2** (1.0 equiv), TsCl, (1.1 equiv), Et_3_N (2.1 equiv), EtOAc (0.2 M), r.t., overnight; remove solvent; then NaOMe (2.2 equiv), MeOH (2.5 M), r.t., 2–6 h.

While Meldrum’s acid itself gave only a moderate 29% yield of the (4-pyridyl)acetate **3k**, the efficiency of the process for substituted variants **3l**–**s** was relatively unaffected by the nature of the side chain, with all yields falling in the range 52–65%. In all cases, the products were isolated as single regioisomers (4-substitution).

Finally, we examined the scope of the nucleophilic partner (Table 3). Activation of 4-methylpyridine-*N*-oxide **1d** with tosyl chloride and substitution with methyl-substituted Meldrum’s acid **2a** was carried out as normal; following removal of the solvent, the resulting crude material was exposed to different nucleophilic ring-opening conditions. The formation of different esters was achieved conveniently by treating the intermediate with a mixture of the relevant alcohol and potassium *tert*-butoxide in THF at room temperature. In this way, benzyl ester **3t** and allyl ester **3u** were prepared in yields similar to that of **3d** from the methanolytic procedure. We also investigated the use of an organometallic nucleophile and found that exposure to *iso*-butylmagnesium bromide at −40 °C gave, after warming to room temperature and aqueous workup, the ketone **3v** in 39% yield. Finally, we examined the use of amine nucleophiles. The crude intermediate was taken up in toluene, the relevant amine was added, and the mixture was heated in a sealed microwave vial at 200 °C for 20 min. Following cooling, extractive workup, and purification, good yields of the resulting amides **3w**–**ab** were obtained. The reaction worked well with both primary and secondary amines, including less nucleophilic aromatic amines such as indoline.

**Table 3:**
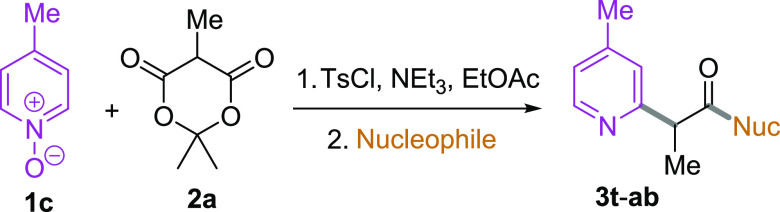

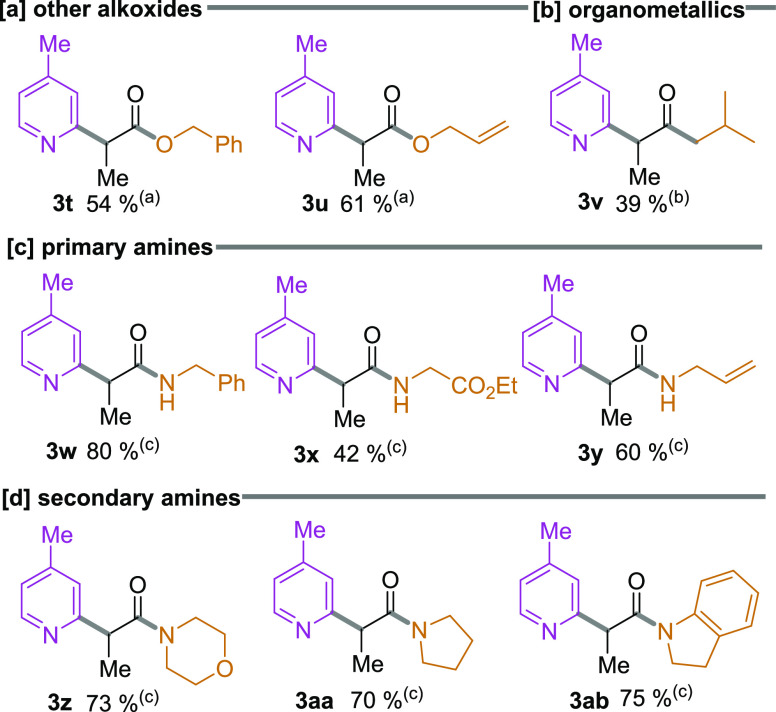
Three-Component Coupling: Scope of the Nucleophile

a Step 2: alcohol (2.5 equiv), KO^t^Bu (1 equiv), THF, r.t.

b Step 2: iBuMgBr (2 M in diethyl ether, 2 equiv), THF, −40 °C to r.t.

c Step 2: amine (2.5 equiv), toluene, microwave, 200 °C.

## 3 Conclusions

In summary, a convenient new approach to the synthesis of diverse substituted 2-(pyridyl)acetic acid derivatives is reported utilizing Meldrum’s acids as linchpin reagents, acting initially as nucleophiles to effect substitution at activated pyridine-*N*-oxides, and subsequently as electrophiles to trigger ring-opening and decarboxylation. Distinct from approaches that utilize halopyridine substrates, the process avoids the need for metal catalysts, preformed enolate equivalents (e.g., silyl ketene acetals), or strongly basic species, as well as obviates the need to handle potentially decarboxylation-sensitive pyridylacetic acids themselves. Additionally, the three-component nature of the process lends itself well to the ready generation of analogues through parallel synthesis protocols. The method therefore complements existing approaches to this class of biologically relevant molecules, and we hope it will prove useful to the community.

## Supporting Information Available

Data underlying this study are available in the published article and its online supplementary material; full experimental procedures and spectroscopic characterization for all reactions and synthesis of Meldrum’s acid derivatives and copies of the ^1^H and ^13^C NMR spectra of products **3** (PDF)
