# Supplementary material for: Three-Component Synthesis of Pyridylacetic Acid Derivatives by Arylation/Decarboxylative Substitution of Meldrum’s Acids
Source: J Org Chem. 2022 Oct 18;87(21):13891–4. doi: 10.1021/acs.joc.2c01597 (PMC9638998; doi:10.1021/acs.joc.2c01597)

Three-Component Synthesis of Pyridylacetic Acid Derivatives by Arylation/Decarboxylative Substitution of Meldrum's Acids

Tarn C. Johnson and Stephen P. Marsden*

(* s.p.marsden@leeds.ac.uk)

Supporting Information

S1 Table of Contents

S2 Experimental Section

S2 General Experimental

S3-S6 Synthesis of Substituted Meldrum's Acids **2**

S7-S19 Synthesis of Products **3**

S20-S48 ^{1}H and ^{13}C NMR spectra of Products **3**

EXPERIMENTAL SECTION

General Experimental

All non-aqueous reactions were performed under an atmosphere of nitrogen unless otherwise stated. Water-sensitive reactions were performed in oven-dried glassware, cooled under nitrogen before use. Dichloromethane and toluene were dried and purified by means of a Pure Solv MD solvent purification system (Innovative Technology Inc.). Ethyl acetate was dried over 3 Å molecular sieves. All other solvents used were of chromatography or analytical grade. Commercially available starting materials were obtained from Sigma-Aldrich, Fluka, Acros, Alfa-Aesar or Fluorochem and were used without purification.

Thin layer chromatography (TLC) was carried out on aluminium backed silica plates (Merck silica gel 60 F254). Visualisation of the plates was achieved using an ultraviolet lamp (λ_{max} = 254 nm) and $KMnO_4$. Flash chromatography was carried out using silica gel 60 (60-63 µm particles) supplied by Merck. Strong cation exchange solid phase extraction (SCX-SPE) was carried out using pre-packed Discovery DSC-SCX cartridges supplied by Supelco.

Infrared spectra were recorded on a Perkin-Elmer Spectrum One FT-IR spectrometer with absorption reported in wavenumbers (cm^{-1}). High resolution mass spectra (HRMS) were recorded on a Bruker MaXis Impact spectrometer with electrospray ionisation (ESI) source. Melting points were determined on a Reichert hot stage microscope and are uncorrected. Proton (^1H) and carbon ($^{13}C\{^1H\}$) NMR spectral data were collected on Bruker Advance 500, Bruker DPX500 or DPX300 spectrometers. Chemical shifts (δ) are quoted in parts per million (ppm) and referenced to residual solvent peaks or tetramethylsilane. Coupling constants (*J*) are quoted in Hertz (Hz) and splitting patterns reported in an abbreviated manner: s (singlet), d (doublet), t (triplet), q (quartet), m (multiplet). Assignments were made with the aid of COSY, DEPT-135 and HMQC experiments.

Meldrum's acid and the 5-Me homologue **2a** were commercially available. Pyridine *N*-oxides were commercially available and used without further purification with the exception of the 4-^{t}Bu and 4-Br variants which were synthesised according to published procedures.[1,2]

Synthesis of Substituted Meldrum's Acids 2b-i

General Procedure A (Modification of the procedure of Fillion *et al.*[3])
Sodium triacetoxyborohydride (2.0 equiv) was added to a solution of aldehyde (1.0 equiv) and Meldrum's acid (1.05 equiv) in dichloroethane (final concentration 0.5 M in aldehyde) at 0 °C. The mixture was allowed to warm to room temperature and was stirrred overnight after which a saturated $NaHCO_3$ solution (20 mL) was added and the mixture was extracted with dichloromethane (3 × 20 mL). The combined organic extracts were dried ($MgSO_4$), filtered and the solvent was removed under reduced pressure.

2,2-Dimethyl-5-(2-methylpropyl)-1,3-dioxane-4,6-dione 2b

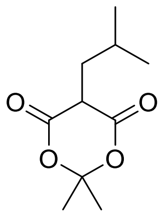

Made following general procedure A on a 2 mmol scale. Recrystallisation from ethyl acetate/pentane gave the product as colourless crystals (195 mg, 0.97 mmol, 49 %). The reported data is in accordance with the literature.[5] **Mp** 124-125 °C (EtOAc/pentane), lit.[5] 119-120 °C (EtOAc); **^{1}H NMR ($CDCl_3$, 500 MHz) δ_H** 3.43 (t, 1H, *J* = 5.6 Hz), 1.95-2.06 (m, 3H), 1.79 (s, 3H), 1.75 (s, 3H), 0.90-0.99 (m, 6H); **^{13}C{^{1}H} NMR ($CDCl_3$, 125 MHz) δ_C** 166.0, 104.8, 44.2, 35.4, 28.6, 26.8, 25.9, 22.1; **HRMS (ESI) m/z:** $[M+H]^+$ Calcd for $C_{10}H_{17}O_4$ 201.1121; Found 201.1120; **ν_{max}** 2950, 1789, 1744, 1377, 1329, 1302, 1205, 1125, 1050, 997, 917 cm^{-1}.

(*E*)-2,2-Dimethyl-5-(3-Phenylprop-2-enyl)-1,3-dioxane-4,6-dione 2c

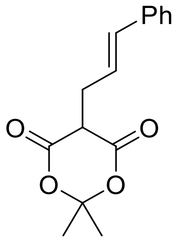

Made following general procedure A on a 2 mmol scale. Recrystallisation from ethyl acetate/pentane gave the product as yellow crystals (315 mg, 1.21 mmol, 61 %). The reported data is in accordance with the literature.[4] **Mp** 113-114 °C (EtOAc/pentane), lit.[4] 108-109 °C (hexane/PhMe); **^{1}H NMR ($CDCl_3$, 500 MHz) δ_H** 7.32-7.38 (m, 2H), 7.27-7.32 (m, 2H), 7.19-7.24 (m,

1H), 6.60 (d, 1H, J = 15.8 Hz), 6.22-6.30 (m, 1H), 3.66 (t, 1H, J = 5.0 Hz), 3.00-3.06 (m, 2H), 1.78 (s, 3H), 1.74 (s, 3H); **$^{13}C\{^1H\}$ NMR ($CDCl_3$, 125 MHz) δ_C** 164.9, 136.8, 134.6, 128.5, 127.6, 126.3, 123.9, 105.0, 46.6, 29.6, 28.4, 26.9; **HRMS (ESI) m/z:** $[M+H]^+$ Calcd for $C_{15}H_{17}O_4$ 261.1121; Found 261.1123; **ν_{max}** 1774, 1738, 1300, 1202, 1038, 918, 737, 692 cm^{-1}.

2,2-Dimethyl-5-(2-methylpropyl)-1,3-dioxane-4,6-dione 2d

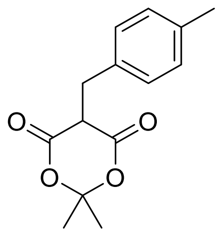

Made following general procedure A on a 1.7 mmol scale. Recrystallisation from ethyl acetate/pentane gave the product as colourless crystals (310 mg, 1.25 mmol, 72 %). The reported data is in accordance with the literature.[4] **Mp** 107-109 °C (EtOAc/pentane), lit.[4] 98 °C; **^1H NMR ($CDCl_3$, 500 MHz) δ_H** 7.18-7.24 (m, 2H), 7.07-7.14 (m, 2H), 3.74 (t, 1H, J = 5.0 Hz), 3.44 (d, 2H, J = 5.0 Hz), 2.30 (s, 3H), 1.72 (s, 3H), 1.50 (s, 3H); **$^{13}C\{^1H\}$ NMR ($CDCl_3$, 125 MHz) δ_C** 165.3, 136.7, 134.1, 129.6, 129.2, 105.1, 48.1, 31.7, 28.4, 27.2, 21.0; **HRMS (ESI) m/z:** $[M+H]^+$ Calcd for $C_{14}H_{17}O_4$ 249.1121; Found 249.1128; **ν_{max}** 1785, 1744, 1311, 1273, 1200, 1090, 1060, 1025, 940, 912, 793 cm^{-1}.

2,2-Dimethyl-5-(4-nitrophenylmethyl)-1,3-dioxane-4,6-dione 2e

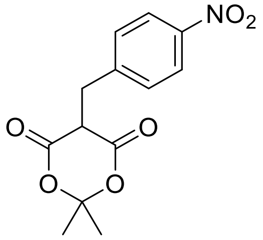

Made following general procedure A on a 3 mmol scale. Recrystallisation from ethyl acetate/pentane gave the product as yellow crystals (432 mg, 1.55 mmol, 45 %). The reported data is in accordance with the literature.[4] **Mp** 139-140 °C (EtOAc/pentane), lit.[4] 120 °C (EtOAc); **^1H NMR ($CDCl_3$, 500 MHz) δ_H** 8.10-8.15 (m, 2H), 7.50-7.55 (m, 2H), 3.83 (t, 1H, J = 5.1 Hz), 3.57 (d, 2H, J = 5.1 Hz), 1.79 (s, 3H), 1.67 (s, 3H); **$^{13}C\{^1H\}$ NMR ($CDCl_3$, 125 MHz) δ_C** 164.6, 147.4, 144.8, 130.8, 123.7, 105.4, 47.8, 31.7, 28.4, 27.0; **HRMS (ESI) m/z:** $[M+H]^+$ Calcd for $C_{13}H_{14}NO_6$ 280.0816; Found 280.0814; **ν_{max}** 1781, 1737, 1520, 1347, 1315, 1197, 1064, 943, 912, 802, 742, 697 cm^{-1}.

5-(4-Methoxyphenylmethyl)-2,2-dimethyl-1,3-dioxane-4,6-dione 2f

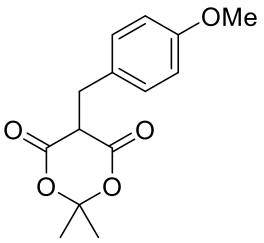

Made following general procedure A on a 3.5 mmol scale. The product was recrystallized twice from diethyl ether to give yellow crystals (759 mg, 2.87 mmol, 83 %). The reported data is in accordance with the literature.[5] **Mp** 77-79 °C (EtOAc/pentane), lit.[5] 82-85 °C (EtOAc); **^{1}H NMR (CDCl$_3$, 500 MHz) δ_H** 7.21-7.25 (m, 2H), 6.79-6.83 (m, 2H), 3.77 (s, 3H), 3.72 (t, 1H, *J* = 4.8 Hz), 3.43 (d, 2H, *J* = 4.8 Hz), 1.71 (s, 3H), 1.48 (s, 3H); **^{13}C{^{1}H} NMR (CDCl$_3$, 125 MHz) δ_C** 165.3, 159.0, 130.9, 129.3, 114.1, 105.1, 55.2, 48.4, 31.7, 28.5, 27.4; **HRMS (ESI) m/z:** $[M+H]^+$ Calcd for $C_{14}H_{17}O_5$ 265.1071; Found 265.1072; **ν_{max}** 1783, 1743, 1513, 1329, 1282, 1242, 1205, 1174, 1056, 1030, 1000, 949, 827, 798 cm^{-1}.

5-(4-Bromophenylmethyl)-2,2-dimethyl-1,3-dioxane-4,6-dione 2g

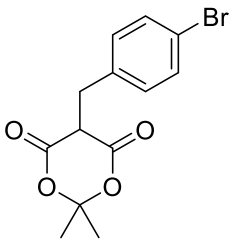

Made following general procedure A on a 3.5 mmol scale. Recrystallisation from ethyl acetate/pentane gave the product as colourless crystals (963 mg, 3.08 mmol, 89 %). The reported data is in accordance with the literature.[4] **Mp** 136-137 °C (EtOAc/pentane), lit.[4] 128 °C (EtOAc); **^{1}H NMR (CDCl$_3$, 500 MHz) δ_H** 7.38-7.43 (m, 2H), 7.19-7.23 (m, 2H), 3.74 (t, 1H, *J* = 5.0 Hz), 3.42 (d, 2H, *J* = 5.0 Hz), 1.74 (s, 3H), 1.58 (s, 3H); **^{13}C{^{1}H} NMR (CDCl$_3$, 125 MHz) δ_C** 164.9, 136.3, 131.7, 131.6, 121.3, 105.2, 48.0, 31.6, 28.5, 27.2; **HRMS (ESI) m/z:** $[M+H]^+$ Calcd for $C_{13}H_{14}{}^{79}BrO_4$ 313.0070; Found 313.0069; **ν_{max}** 1784, 1737, 1360, 1328, 1288, 1203, 1059, 1007, 953, 937, 884, 780 cm^{-1}.

2,2-Dimethyl-5-(2-furylmethyl)-1,3-dioxane-4,6-dione 2h

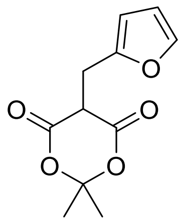

Made following general procedure A on a 2 mmol scale. Recrystallisation from ethyl acetate/pentane gave the product as colourless crystals (226 mg, 1.01 mmol, 50 %). The reported

data is in accordance with the literature.[4] **Mp** 93-94 °C (EtOAc/pentane), lit.[6] 92-93 °C (hexane/PhMe); **^{1}H NMR ($CDCl_3$, 500 MHz) δ_H** 7.27-7.31 (m, 1H), 6.26-6.30 (m, 1H), 6.10-6.19 (m, 1H), 3.84 (t, 1H, *J* = 5.0 Hz), 3.50 (d, 2H, *J* = 5.0 Hz), 1.78 (s, 3H), 1.67 (s, 3H); **^{13}C{^{1}H} NMR ($CDCl_3$, 125 MHz) δ_C** 164.7, 150.6, 141.5, 110.6, 107.8, 105.2, 45.4, 28.3, 27.0, 24.9; **HRMS (ESI) m/z:** $[M+H]^+$ Calcd for $C_{11}H_{13}O_5$ 225.0758; Found 225.0754; **ν_{max}** 1782, 1739, 1359, 1322, 1292, 1236, 1202, 1066, 1023, 955, 942, 890, 804, 738, 720 cm^{-1}.

2,2-Dimethyl-5-(2-thienylmethyl)-1,3-dioxane-4,6-dione 2i

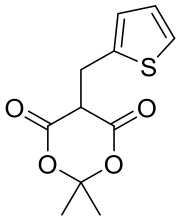

Made following general procedure A on a 3 mmol scale. The residue was crystallised from ethyl acetate/pentane, dissolved in dichloromethane (10 mL) and extracted with saturated aqueous $NaHCO_3$ (3 x 10 mL). The combined aqueous extracts were acidified with 1 M HCl solution and extracted with dichloromethane (3 × 40 mL). After the combined organic extracts were dried ($MgSO_4$), filtered and the solvent removed under reduced pressure a colourless solid was obtained (343 mg, 1.43 mmol, 41 %). The reported data is in accordance with the literature.[7] **Mp** 96-97 °C (EtOAc/pentane), lit.[7] 100 °C (EtOH); **^{1}H NMR ($CDCl_3$, 500 MHz) δ_H** 7.13-7.17 (m, 1H), 6.96-6.99 (m, 1H), 6.89-6.93 (m, 1H), 3.77 (t, 1H, *J* = 4.7 Hz), 3.69 (d, 2H, *J* = 4.7 Hz), 1.76 (s, 3H), 1.59 (s, 3H); **^{13}C{^{1}H} NMR ($CDCl_3$, 125 MHz) δ_C** 164.8, 138.4, 127.7, 126.9, 124.9, 105.2, 48.4, 28.5, 27.2, 26.6; **HRMS (ESI) m/z:** $[M+H]^+$ Calcd for $C_{11}H_{13}O_4S$ 241.0529; Found 241.0526; **ν_{max}** 2876, 1776, 1740, 1347, 1295, 1200, 1138, 1069, 1042, 932, 875, 733 cm^{-1}.

Arylation of Meldrum's Acids

General Procedure B - Methanolysis

Triethylamine (0.147 mL, 1.05 mmol) was added to a solution of Meldrum's acid derivative (0.50 mmol), 4-toluenesulfonyl chloride (105 mg, 0.55 mmol) and pyridine-*N*-oxide (0.55 mmol) in ethyl acetate (2.5 mL) and the mixture was allowed to stir at RT overnight after which the solvent was removed under reduced pressure. Methanol (2 mL) was added followed by sodium methoxide (60 mg, 1.10 mmol) and the mixture was stirred for a further 2-6 h (LCMS) prior to filtration and removal of the volatiles under reduced pressure.

General Procedure C – Opening with other alcohols

Triethylamine (0.147 mL, 1.05 mmol) was added to a solution of 2,2,5-trimethyl-1,3-dioxane-4,6-dione (79 mg, 0.50 mmol), 4-toluenesulfonyl chloride (105 mg, 0.55 mmol) and 4-methylpyridine-*N*-oxide (60 mg, 0.55 mmol) in ethyl acetate (2.5 mL) and the mixture was allowed to stir at RT overnight after which water (10 mL) was added and the mixture was extracted with ethyl acetate (3 × 10 mL). The combined organic extracts were dried ($MgSO_4$), filtered and the solvent was removed under reduced pressure. Tetrahydrofuran (2 mL) was added followed by KO^{t}Bu (168 mg, 0.50 mmol) and alcohol (1.25 mmol) and the mixture was stirred for a further 2-6 h (LCMS) at RT prior to the addition of water (10 mL) and extraction with ethyl acetate (3 × 10 mL). The combined organic extracts were combined, dried ($MgSO_4$), filtered and the volatiles were removed under reduced pressure.

General Procedure D – Opening with amines

Triethylamine (0.147 mL, 1.05 mmol) was added to a solution of 2,2,5-trimethyl-1,3-dioxane-4,6-dione (79 mg, 0.50 mmol), 4-toluenesulfonyl chloride (105 mg, 0.55 mmol) and 4-methylpyridine-*N*-oxide (60 mg, 0.55 mmol) in ethyl acetate (2.5 mL) and the mixture was allowed to stir at RT overnight after which the solvent was removed under reduced pressure. Toluene (2 mL) was added and the mixture was transferred to a thick-walled glass vial containing the amine (1.25 mmol) which was capped and heated in a microwave at 200 °C for 20 minutes (CEM Discover SP, using auto-temperature ramp to a fixed temperature of 200 °C, monitored using internal infra-red sensor, maximum pressure 300 psi, maximum power 300 W). Water (10 mL) was added and the mixture was extracted with ethyl acetate (3 × 10 mL). The combined organic extracts were dried ($MgSO_4$), filtered and the volatiles were removed under reduced pressure.

Methyl 2-(pyridin-4-yl)propanoate, 3a

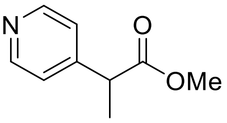

Made following general procedure B. Column chromatography on silica with a gradient elution from 20-100 % ethyl acetate in hexane gave the product as a yellow oil (52 mg, 0.32 mmol, 63 %) whose spectral data matched that in the literature.[8] **^{1}H NMR ($CDCl_3$, 500 MHz) δ_H** 8.49-8.54 (m, 2H), 7.17-7.22 (m, 2H), 3.68 (q, 1H, *J* = 7.2 Hz), 3.64 (s, 3H), 1.47 (d, 3H, *J* = 7.2 Hz); **$^{13}C\{^1H\}$ NMR ($CDCl_3$, 125 MHz) δ_C** 173.5, 149.9, 149.1, 122.7, 52.2, 44.8, 17.8; **HRMS (ESI) m/z:** $[M+H]^+$ Calcd for $C_9H_{12}NO_2$ 166.0863; Found 166.0858; **ν_{max}** 2984, 1733, 1597, 1435, 1209, 1165, 1103 cm^{-1}.

Larger-scale preparation: 4-Toluenesulfonyl chloride (260 mg, 1.36 mmol) was added to pyridine-*N*-oxide (130 mg, 1.36 mmol) in dry EtOAc (4 mL) and the mixture stirred for 60 minutes, during which time a fine cloudy suspension formed. A solution of 2,2,5-trimethyl-1,3-dioxane-4,6-dione (197 mg, 1.24 mmol) in EtOAc (1 mL) was added, followed by triethylamine (350 μL, 2.54 mmol), and the mixture left to stir for 20.5 hours, after which time a cream precipitate had formed. The solvent was removed *in vacuo*, and the residue taken up in dry MeOH (5.5 mL) prior to the addition of sodium methoxide (150 mg, 2.73 mmol), and the mixture left to stir for 24 hours. The solvent was removed *in vacuo* and the residue purified by column chromatography (gradient 20-60% EtOAc:hexanes) to yield the product as a colourless oil (102 mg, 50%).

Methyl 2-(2-methylpyridin-4-yl)propanoate, 3b

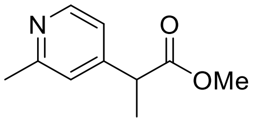

Made following general procedure B. Column chromatography on silica with a gradient elution from 0-100 % ethyl acetate in hexane gave the product as a yellow oil (44 mg, 0.25 mmol, 49 %). **^{1}H NMR ($CDCl_3$, 500 MHz) δ_H** 8.37-8.41 (m, 1H), 7.02-7.06 (m, 1H), 6.97-7.01 (m, 1H), 3.59-3.65 (m, 4H), 2.50 (s, 3H), 1.44 (d, 3H, *J* = 7.2 Hz); **$^{13}C\{^1H\}$ NMR ($CDCl_3$, 125 MHz) δ_C** 173.6, 158.6, 149.5, 149.1, 122.3, 119.9, 52.2, 44.8, 24.1, 17.8; **HRMS (ESI) m/z:** $[M+H]^+$ Calcd for $C_{10}H_{14}NO_2$ 180.1019; Found 180.1015; **ν_{max}** 2952, 1733, 1602, 1435, 1198, 1161, 1092, 901, 839 cm^{-1}.

Methyl 2-(3-methylpyridin-4-yl)propanoate, 3c

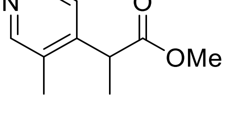

Made following general procedure B. Column chromatography on silica with a gradient elution from 0-100 % ethyl acetate in hexane gave the product as a yellow film (34 mg, 0.19 mmol, 38 %). **^{1}H NMR ($CDCl_3$, 500 MHz) δ_H** 8.34-8.39 (m, 2H), 7.11-7.14 (m, 1H), 3.88 (q, 1H, *J* = 7.1 Hz), 3.64 (s, 3H), 2.30 (s, 3H), 1.45 (d, 3H, *J* = 7.1 Hz); **$^{13}C\{^{1}H\}$ NMR ($CDCl_3$, 125 MHz) δ_C** 173.7, 151.0, 147.9, 147.5, 131.1, 121.1, 52.1, 40.9, 17.1, 16.1; **HRMS (ESI) m/z:** $[M+H]^+$ Calcd for $C_{10}H_{14}NO_2$ 180.1019; Found 180.1018; **ν_{max}** 2952, 1732, 1593, 1454, 1435, 1201, 1092, 863, 838 cm^{-1}.

Methyl 2-(4-methylpyridin-2-yl)propanoate, 3d

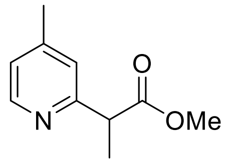

Made following general procedure B. Column chromatography on silica with a gradient elution from 0-40 % ethyl acetate in hexane followed by an SCX column gave the product as a yellow oil (51 mg, 0.29 mmol, 57 %). **^{1}H NMR ($CDCl_3$, 500 MHz) δ_H** 8.33-8.38 (m, 1H), 7.03-7.07 (m, 1H), 6.92-6.98 (m, 1H), 3.86 (q, 1H, *J* = 7.2 Hz), 3.64 (s, 3H), 2.29 (s, 3H), 1.49 (d, 3H, *J* = 7.2 Hz); **$^{13}C\{^{1}H\}$ NMR ($CDCl_3$, 125 MHz) δ_C** 174.0, 159.5, 149.0, 147.9, 123.0, 122.7, 51.9, 47.6, 20.9, 17.1; **HRMS (ESI) m/z:** $[M+H]^+$ Calcd for $C_{10}H_{14}NO_2$ 180.1019; Found 180.1020; **ν_{max}** 2951, 1734, 1604, 1406, 1248, 1198, 1075, 900, 829 cm^{-1}.

Methyl 2-(4-*tert*-butylpyridin-2-yl)propanoate, 3e

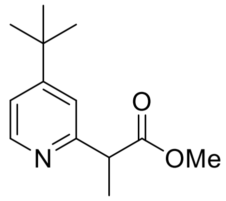

Made following general procedure B. Column chromatography on silica with a gradient elution from 0-100 % ethyl acetate in hexane followed by an SCX column gave the product as a colourless oil (65 mg, 0.29 mmol, 59 %). **^{1}H NMR ($CDCl_3$, 500 MHz) δ_H** 8.40-8.43 (m, 1H), 7.19-7.22 (m, 1H), 7.11-7.15 (m, 1H), 3.90 (q, 1H, *J* = 7.2 Hz), 3.66 (s, 3H), 1.52 (d, 3H, *J* = 7.2 Hz), 1.27 (s, 9H); **$^{13}C\{^{1}H\}$ NMR ($CDCl_3$, 125 MHz) δ_C** 174.1, 160.8, 159.6, 149.1, 119.2, 118.8, 52.0, 47.9, 34.7, 30.4, 17.3; **HRMS (ESI) m/z:** $[M+H]^+$ Calcd for $C_{13}H_{20}NO_2$ 222.1489; Found 222.1491; **ν_{max}** 2963, 1736, 1599, 1550, 1434, 1402, 1212, 1165, 1076, 890, 842 cm^{-1}.

Methyl 2-(4-phenylpyridin-2-yl)propanoate, 3f

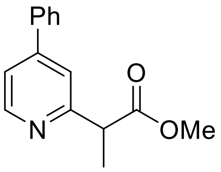

Made following general procedure B. Column chromatography on silica with a gradient elution from 0-40 % ethyl acetate in hexane gave the product as a yellow oil (74 mg, 0.31 mmol, 61 %). **^{1}H NMR ($CDCl_3$, 500 MHz) δ_H** 8.56-8.61 (m, 1H), 7.58-7.64 (m, 2H), 7.35-7.51 (m, 5H), 4.01 (q, 1H, *J* = 7.2 Hz), 3.69 (s, 3H), 1.60 (d, 3H, *J* = 7.2 Hz); **$^{13}C\{^1H\}$ NMR ($CDCl_3$, 125 MHz) δ_C** 173.9, 160.3, 149.7, 149.2, 138.0, 128.9, 126.9, 120.1, 119.9, 52.0, 47.8, 17.2, one ArC not found; **HRMS (ESI) m/z:** $[M+H]^+$ Calcd for $C_{15}H_{16}NO_2$ 242.1176; Found 242.1176; **ν_{max}** 2949, 1732, 1596, 1547, 1204, 1163, 1076, 762, 696, 614 cm^{-1}.

Larger-scale preparation: 4-Toluenesulfonyl chloride (260 mg, 1.36 mmol) was added to 4-phenylpyridine-*N*-oxide (235 mg, 1.36 mmol) in dry EtOAc (4 mL) and the mixture stirred for 60 minutes. A solution of 2,2,5-trimethyl-1,3-dioxane-4,6-dione (197 mg, 1.24 mmol) in EtOAc (1 mL) was added, followed by triethylamine (350 μL, 2.54 mmol), and the mixture left to stir for 20.5 hours, after which time a cream precipitate had formed. The solvent was removed *in vacuo*, and the residue taken up in dry MeOH (5.5 mL) prior to the addition of sodium methoxide (150 mg, 2.73 mmol), and the mixture left to stir for 24 hours. The solvent was removed *in vacuo* and the residue purified by column chromatography (gradient 20-40% EtOAc:hexanes) to yield the product as a colourless oil (201 mg, 61%).

Methyl 2-(4-methoxypyridin-2-yl)propanoate, 3g

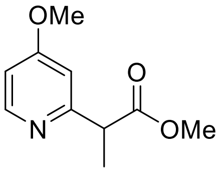

Made following general procedure B. Column chromatography on silica with a gradient elution from 0-100 % ethyl acetate in hexane gave the product as a colourless oil (33 mg, 0.17 mmol, 34 %). **^{1}H NMR ($CDCl_3$, 500 MHz) δ_H** 8.32-8.37 (m, 1H), 6.75-6.79 (m, 1H), 6.66-6.70 (m, 1H), 3.87 (q, 1H, *J* = 7.2 Hz), 3.81 (s, 3H), 3.66 (s, 3H), 1.51 (d, 3H, *J* = 7.2 Hz); **$^{13}C\{^1H\}$ NMR ($CDCl_3$, 125 MHz) δ_C** 173.9, 166.3, 161.3, 150.4, 108.2, 108.0, 55.0, 52.0, 47.7, 17.1; **HRMS (ESI) m/z:** $[M+H]^+$ Calcd for $C_{10}H_{14}NO_3$ 196.0968; Found 196.0968; **ν_{max}** 2949, 1733, 1593, 1567, 1456, 1302, 1202, 1160, 1035, 831 cm^{-1}.

Methyl 2-(4-bromopyridin-2-yl)propanoate, 3h

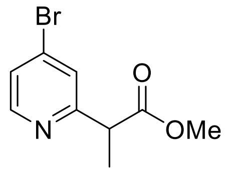

Made following general procedure B. Column chromatography on silica with a gradient elution from 0-50 % ethyl acetate in hexane gave the product as a colourless oil (25 mg, 0.10 mmol, 21 %) whose spectral data matched that in the literature.[8] **^{1}H NMR ($CDCl_3$, 300 MHz) δ_H** 8.31-8.40 (m, 1H), 7.42-7.49 (m, 1H), 7.30-7.38 (m, 1H), 3.90 (q, 1H, *J* = 7.2 Hz), 3.68 (s, 3H), 1.53 (d, 3H, *J* = 7.2 Hz); **^{13}C{^{1}H} NMR ($CDCl_3$, 125 MHz) δ_C** 173.4, 161.3, 150.1, 133.4, 125.52, 125.50, 52.3, 47.6, 17.1; **HRMS (ESI) m/z:** $[M+H]^+$ Calcd for $C_9H_{11}{}^{79}BrNO_2$ 243.9968; Found 243.9962; **ν_{max}** 2950, 1733, 1568, 1553, 1463, 1434, 1386, 1329, 1210, 1165, 1091, 867, 825, 682 cm^{-1}.

Methyl 2-(isoquinolin-1-yl)propanoate, 3i

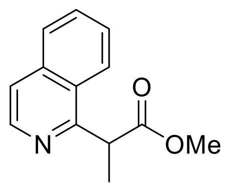

Made following general procedure B. Column chromatography on silica with a gradient elution from 0-50 % ethyl acetate in hexane gave the product as a yellow oil (20 mg, 0.09 mmol, 19 %) whose spectral data matched that in the literature.[9] **^{1}H NMR ($CDCl_3$, 500 MHz) δ_H** 8.48-8.51 (m, 1H), 8.13-8.17 (m, 1H), 7.82-7.85 (m, 1H), 7.65-7.70 (m, 1H), 7.59-7.64 (m, 1H), 7.54-7.57 (m, 1H), 4.73 (q, 1H, *J* = 7.1 Hz), 3.68 (s, 3H), 1.70 (d, 3H, *J* = 7.1 Hz); **^{13}C{^{1}H} NMR ($CDCl_3$, 125 MHz) δ_C** 174.0, 159.5, 142.0, 136.6, 129.9, 127.6, 127.5, 126.5, 124.5, 120.1, 52.2, 44.3, 16.7; **HRMS (ESI) m/z:** $[M+H]^+$ Calcd for $C_{13}H_{14}NO_2$ 216.1019; Found 216.1023; **ν_{max}** 2949, 1735, 1624, 1585, 1562, 1501, 1454, 1384, 1354, 1317, 1202, 1092, 1004, 926, 873, 825, 748 cm^{-1}.

Methyl 2-(quinolin-2-yl)propanoate, and methyl 2-(quinolin-4-yl)propanoate, 3j

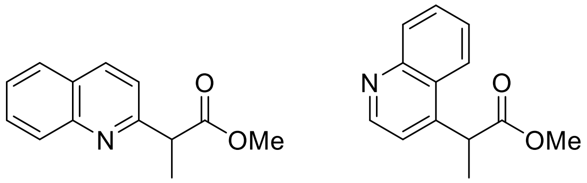

Made following general procedure B. Purification by SCX column followed by column chromatography on silica with a gradient elution from 0-60 % ethyl acetate in hexane gave the 2-regioisomer as a yellow film (10 mg, 0.05 mmol, 9 %) and the 4-regioisomer as a yellow film (7 mg,

0.03 mmol, 7 %). The spectral data for the known 2-regioisomer matched that in the literature.[9] **2-Regioisomer; ^{1}H NMR (CDCl$_3$, 500 MHz) δ_H** 8.11-8.15 (m, 1H), 8.06-8.09 (m, 1H), 7.77-7.81 (m, 1H), 7.67-7.72 (m, 1H), 7.49-7.54 (m, 1H), 7.40-7.44 (m, 1H), 4.16 (q, 1H, *J* = 7.2 Hz), 3.70 (s, 3H), 1.66 (d, 3H, *J* = 7.2 Hz); **^{13}C{^{1}H} NMR (CDCl$_3$, 125 MHz) δ_C** 174.0, 160.0, 147.8, 136.8, 129.5, 129.3, 127.5, 127.2, 126.3, 119.8, 52.1, 48.8, 17.3; **HRMS (ESI) m/z:** [M+H]$^+$ Calcd for $C_{13}H_{14}NO_2$ 216.1019**;** Found 216.1016; **ν_{max}** 2950, 1732, 1598, 1503, 1429, 1307, 1251, 1204, 1166, 1070, 832, 754 cm^{-1}. **4-Regioisomer; ^{1}H NMR (CDCl$_3$, 500 MHz) δ_H** 8.86-8.90 (m, 1H), 8.13-8.17 (m, 1H), 8.05-8.09 (m, 1H), 7.70-7.75 (m, 1H), 7.57-7.62 (m, 1H), 7.35-7.38 (m, 1H), 4.51 (q, 1H, *J* = 7.2 Hz), 3.67 (s, 3H), 1.67 (d, 3H, *J* = 7.2 Hz); **^{13}C{^{1}H} NMR (CDCl$_3$, 125 MHz) δ_C** 174.0, 150.3, 148.5, 146.2, 130.5, 129.3, 126.9, 126.7, 122.9, 119.2, 52.4, 40.8, 17.7; **HRMS (ESI) m/z:** [M+H]$^+$ Calcd for $C_{13}H_{14}NO_2$ 216.1019; Found 216.1022; **ν_{max}** 2951, 1732, 1590, 1569, 1509, 1434, 1199, 1172, 1092, 1060, 872, 760 cm^{-1}.

Methyl 2-(pyridin-4-yl)ethanoate, 3k

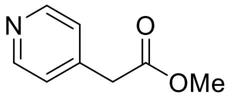

Made following general procedure B. An SCX column followed by column chromatography on silica with a gradient elution from 20-100 % ethyl acetate in hexane gave the product as a yellow film (22 mg, 0.15 mmol, 29 %) whose spectral data matched that in the literature.[10] **^{1}H NMR (CDCl$_3$, 500 MHz) δ_H** 8.53-8.57 (m, 2H), 7.19-7.24 (m, 2H), 3.71 (s, 3H), 3.62 (s, 2H); **^{13}C{^{1}H} NMR (CDCl$_3$, 125 MHz) δ_C** 170.4, 149.9, 142.7, 124.5, 52.3, 40.4; **HRMS (ESI) m/z:** [M+H]$^+$ Calcd for $C_8H_{10}NO_2$ 152.0706; Found 152.0702; **ν_{max}** 2954, 1733, 1600, 1562, 1417, 1228, 1161, 995, 804 cm^{-1}.

Methyl 2-(pyridin-4-yl)-4-methylpentanoate, 3l

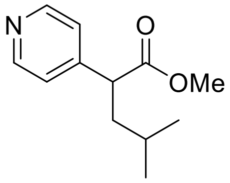

Made following general procedure B. Column chromatography on silica with a gradient elution from 20-100 % ethyl acetate in hexane followed by an SCX column gave the product as a yellow oil (54 mg, 0.26 mmol, 52 %). **^{1}H NMR (CDCl$_3$, 500 MHz) δ_H** 8.47-8.53 (m, 2H), 7.17-7.23 (m, 2H), 3.57-3.65 (m, 4H), 1.87-1.98 (m, 1H), 1.57-1.66 (m, 1H), 1.35-1.46 (m, 1H), 0.82-0.92 (m, 6H): **^{13}C{^{1}H} NMR (CDCl$_3$, 125 MHz) δ_C** 173.2, 149.9, 148.0, 123.1, 52.1, 49.0, 41.9, 25.8, 22.4, 22.0; **HRMS (ESI) m/z:** [M+H]$^+$ Calcd for $C_{12}H_{18}NO_2$ 208.1332; Found 208.1332; **ν_{max}** 2955, 1734, 1597, 1416, 1278, 1239, 1199, 1161, 992, 832, 540 cm^{-1}.

Methyl 2-(pyridin-4-yl)-2-cinnamylethanoate, 3m

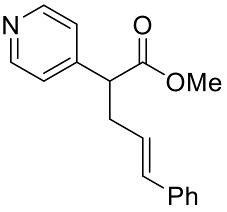

Made following general procedure B. Column chromatography on silica with a gradient elution from 20-100 % ethyl acetate in hexane followed by an SCX column gave the product as a yellow oil (72 mg, 0.27 mmol, 54 %). **^{1}H NMR ($CDCl_3$, 500 MHz) δ_H** 8.54-8.59 (m, 2H), 7.23-7.29 (m, 6H), 7.17-7.23 (m, 1H), 6.42 (d, 1H, *J* = 15.8), 6.01-6.09 (m, 1H), 3.66-3.72 (m, 4H), 2.92-3.00 (m, 1H), 2.63-2.70 (m, 1H); **^{13}C{^{1}H} NMR ($CDCl_3$, 125 MHz) δ_C** 172.4, 150.1, 147.0, 136.9, 132.9, 128.4, 127.4, 126.0, 125.6, 123.1, 52.2, 51.1, 36.3; **HRMS (ESI) m/z:** $[M+H]^+$ Calcd for $C_{17}H_{18}NO_2$ 268.1332; Found 268.1338; **ν_{max}** 3026, 1733, 1596, 1416, 1219, 1160, 966, 819, 744, 693 cm^{-1}.

Methyl 2-(pyridin-4-yl)-3-(4-methylphenyl)propanoate, 3n

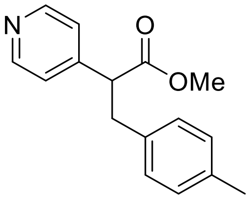

Made following general procedure B. Column chromatography on silica with a gradient elution from 20-100 % ethyl acetate in hexane followed by an SCX column gave the product as a yellow oil (83 mg, 0.33 mmol, 65 %). **^{1}H NMR ($CDCl_3$, 500 MHz) δ_H** 8.48-8.55 (m, 2H), 7.17-7.22 (m, 2H), 6.99-7.05 (m, 2H), 6.92-6.99 (m, 2H), 3.77-3.84 (m, 1H), 3.61 (s, 3H), 3.36 (dd, 1H, *J* = 13.8, 8.3 Hz), 2.97 (dd, 1H, *J* = 13.8, 7.3 Hz), 2.27 (s, 3H); **^{13}C{^{1}H} NMR ($CDCl_3$, 125 MHz) δ_C** 172.5, 149.9, 147.1, 136.1, 134.8, 129.1, 128.6, 123.2, 53.0, 52.1, 38.7, 20.9; **HRMS (ESI) m/z:** $[M+H]^+$ Calcd for $C_{16}H_{18}NO_2$ 256.1332; Found 256.1336; **ν_{max}** 2951, 1733, 1597, 1515, 1435, 1416, 1278, 1217, 1157, 811, 548 cm^{-1}.

Methyl 2-(pyridin-4-yl)-3-(4-bromophenyl)propanoate, 3o

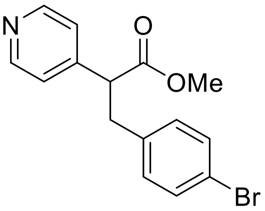

Made following general procedure B. Column chromatography on silica with a gradient elution from 20-100 % ethyl acetate in hexane gave the product as a yellow oil (92 mg, 0.29 mmol, 57 %). **^{1}H NMR ($CDCl_3$, 500 MHz) δ_H** 8.48-8.53 (m, 2H), 7.29-7.34 (m, 2H), 7.13-7.18 (m, 2H), 6.90-6.95 (m, 2H), 3.73-

3.80 (m, 1H), 3.60 (s, 3H), 3.33 (dd, 1H, *J* = 13.8, 8.3 Hz), 2.94 (dd, 1H, *J* = 13.8, 7.3 Hz); **$^{13}C\{^1H\}$ NMR ($CDCl_3$, 125 MHz) δ_C** 172.1, 150.1, 146.8, 137.0, 131.5, 130.5, 123.0, 120.6, 52.7, 52.1, 38.6; **HRMS (ESI) m/z:** $[M+H]^+$ Calcd for $C_{15}H_{15}{}^{79}BrNO_2$ 320.0281; Found 320.0278; **ν_{max}** 2951, 1733, 1597, 1488, 1435, 1415, 1218, 1159, 1071, 1011, 813, 541 cm^{-1}.

Methyl 2-(pyridin-4-yl)-3-(4-methoxyphenyl)propanoate, 3p

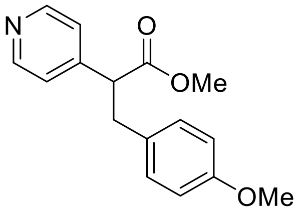

Made following general procedure B. Column chromatography on silica with a gradient elution from 20-100 % ethyl acetate in hexane gave the product as an orange oil (87 mg, 0.32 mmol, 64 %). **^1H NMR ($CDCl_3$, 500 MHz) δ_H** 8.48-8.52 (m, 2H), 7.16-7.20 (m, 2H), 6.94-6.99 (m, 2H), 6.72-6.77 (m, 2H), 3.75-3.80 (m, 1H), 3.72 (s, 3H), 3.60 (s, 3H), 3.32 (dd, 1H, *J* = 13.8, 8.3 Hz), 2.94 (dd, 1H, *J* = 13.8, 7.3 Hz); **$^{13}C\{^1H\}$ NMR ($CDCl_3$, 125 MHz) δ_C** 172.4, 158.5, 149.9, 147.3, 130.0, 129.7, 123.2, 114.0, 55.1, 53.2, 52.0, 38.5; **HRMS (ESI) m/z:** $[M+H]^+$ Calcd for $C_{16}H_{18}NO_3$ 272.1281; Found 272.1282; **ν_{max}** 2953, 1733, 1598, 1512, 1438, 1416, 1245, 1157, 1032, 820, 751, 550 cm^{-1}.

Methyl 2-(pyridin-4-yl)-3-(4-nitrophenyl)propanoate, 3q

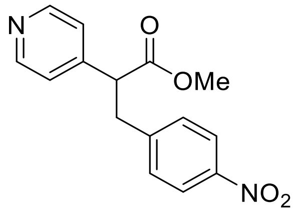

Made following general procedure B. Column chromatography on silica with a gradient elution from 20-100 % ethyl acetate in hexane gave the product as a yellow, crystalline solid (94 mg, 0.33 mmol, 65 %). **Mp** 80-82 °C (EtOAc/pentane); **^1H NMR ($CDCl_3$, 500 MHz) δ_H** 8.54-8.59 (m, 2H), 8.08-8.13 (m, 2H), 7.26-7.32 (m, 2H), 7.20-7.24 (m, 2H), 3.86-3.91 (m, 1H), 3.66 (s, 3H), 3.53 (dd, 1H, *J* = 13.9, 8.5 Hz), 3.15 (dd, 1H, *J* = 13.9, 7.2); **$^{13}C\{^1H\}$ NMR ($CDCl_3$, 125 MHz) δ_C** 171.7, 150.2, 147.1, 146.4, 145.6, 129.7, 123.6, 122.9, 52.4, 52.3, 38.8; **HRMS (ESI) m/z:** $[M+H]^+$ Calcd for $C_{15}H_{15}N_2O_4$ 287.1026; Found 287.1031; **ν_{max}** 1724, 1597, 1513, 1343, 1287, 1246, 1165, 1105, 1016, 856 cm^{-1}.

Methyl 2-(pyridin-4-yl)-3-(furan-2-yl)propanoate, 3r

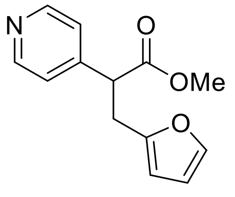

Made following general procedure B. Column chromatography on silica with a gradient elution from 20-100 % ethyl acetate in hexane followed by an SCX column gave the product as a yellow oil (64 mg, 0.28 mmol, 55 %). **^{1}H NMR ($CDCl_3$, 500 MHz) δ_H** 8.48-8.52 (m, 2H), 7.22-7.25 (m, 1H), 7.14-7.18 (m, 2H), 6.15-6.19 (m, 1H), 5.89-5.91 (m, 1H), 3.92-3.97 (m, 1H), 3.63 (s, 3H), 3.39 (dd, 1H, *J* = 15.2, 8.1 Hz), 3.03 (dd, 1H, *J* = 15.2, 7.3 Hz); **^{13}C{^{1}H} NMR ($CDCl_3$, 125 MHz) δ_C** 172.1, 151.6, 150.0, 146.6, 141.5, 122.9, 110.1, 106.8, 52.3, 49.8, 31.3; **HRMS (ESI) m/z:** $[M+H]^+$ Calcd for $C_{13}H_{14}NO_3$ 232.0968; Found 232.0966; **ν_{max}** 2953, 1734, 1597, 1435, 1417, 1204, 1156, 1074, 1011, 921, 808, 734, 599, 529 cm^{-1}.

Methyl 2-(pyridin-4-yl)-3-(thiophen-2-yl)propanoate, 3s

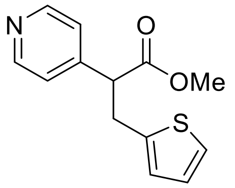

Made following general procedure B. Column chromatography on silica with a gradient elution from 20-100 % ethyl acetate in hexane followed by an SCX column gave the product as an orange oil (77 mg, 0.31 mmol, 62 %). **^{1}H NMR ($CDCl_3$, 500 MHz) δ_H** 8.43-8.48 (m, 2H), 7.11-7.16 (m, 2H), 6.99-7.03 (m, 1H), 6.74-6.78 (m, 1H), 6.60-6.64 (m, 1H), 3.76-3.82 (m, 1H), 3.58 (s, 3H), 3.54 (dd, 1H, *J* = 14.9, 8.1 Hz), 3.17 (dd, 1H, *J* = 14.9, 7.2 Hz); **^{13}C{^{1}H} NMR ($CDCl_3$, 125 MHz) δ_C** 172.0, 150.1, 146.7, 140.1, 126.8, 125.9, 124.1, 123.1, 53.3, 52.2, 33.2; **HRMS (ESI) m/z:** $[M+H]^+$ Calcd for $C_{13}H_{14}NO_2S$ 248.0740; Found 248.0742; **ν_{max}** 2952, 1732, 1596, 1435, 1416, 1217, 1164, 851, 823, 698, 533 cm^{-1}.

Benzyl 2-(4-methylpyridin-2-yl)propanoate, 3t

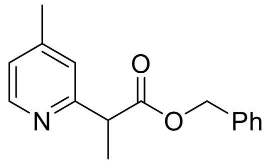

Made following general procedure C. Column chromatography on silica with a gradient elution from 0-40 % ethyl acetate in hexane followed an SCX column gave a yellow oil (69 mg, 0.27 mmol, 54 %). **^{1}H NMR ($CDCl_3$, 500 MHz) δ_H** 8.37-8.41 (m, 1H), 7.22-7.32 (m, 5H), 7.02-7.05 (m, 1H), 6.94-6.98 (m, 1H), 5.14 (s, 2H), 3.95 (q, 1H, *J* = 7.2 Hz), 2.29 (s, 3H), 1.55 (d, 3H, *J* = 7.2 Hz); **^{13}C{^{1}H} NMR ($CDCl_3$, 125**

MHz) δ$_C$ 173.4, 159.5, 149.0, 147.7, 135.9, 128.3, 127.9, 127.8, 123.0, 122.7, 66.3, 47.7, 20.9, 17.1; **HRMS (ESI) m/z:** $[M+H]^+$ Calcd for $C_{16}H_{18}NO_2$ 256.1332; Found 256.1339; **ν_{max}** 2936, 1733, 1604, 1454, 1228, 1176, 1152, 1079, 828, 737, 697 cm^{-1}.

Allyl 2-(4-methylpyridin-2-yl)propanoate, 3u

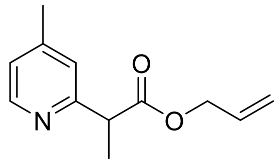

Made following general procedure C. Column chromatography on silica with a gradient elution from 0-40 % ethyl acetate in hexane gave a colourless oil (63 mg, 0.31 mmol, 61 %). **^{1}H NMR ($CDCl_3$, 500 MHz) δ$_H$** 8.33-8.40 (m, 1H), 7.05-7.09 (m, 1H), 6.93-6.98 (m, 1H), 5.78-5.88 (m, 1H), 5.16-5.22 (m, 1H), 5.11-5.16 (m, 1H), 4.53-4.62 (m, 2H), 3.90 (q, 1H, *J* = 7.2 Hz), 2.30 (s, 3H), 1.52 (d, 3H, *J* = 7.2 Hz); **$^{13}C\{^1H\}$ NMR ($CDCl_3$, 125 MHz) δ$_C$** 173.3, 159.5, 149.0, 147.7, 132.0, 123.0, 122.7, 117.8, 66.3, 47.7, 20.9, 17.2; **HRMS (ESI) m/z:** $[M+H]^+$ Calcd for $C_{12}H_{16}NO_2$ 206.1176; Found 206.1181; **ν_{max}** 2937, 1733, 1604, 1453, 1230, 1180, 1154, 1080, 992, 927, 828 cm^{-1}.

5-Methyl-2-(4-methylpyridin-2-yl)hexan-3-one, 3v

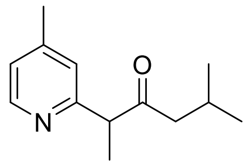

Triethylamine (0.147 mL, 1.05 mmol) was added to a solution of 2,2,5-trimethyl-1,3-dioxane-4,6-dione (79 mg, 0.50 mmol), 4-toluenesulfonyl chloride (105 mg, 0.55 mmol) and 4-methylpyridine-*N*-oxide (60 mg, 0.55 mmol) in ethyl acetate (2.5 mL) and the mixture was allowed to stir at RT overnight after which water (10 mL) was added and the mixture was extracted with EtOAc (3 × 10 mL). The combined organic extracts were dried ($MgSO_4$), filtered and the solvent was removed under reduced pressure. The residue was dissolved in tetrahydrofuran (5 mL), transferred to an oven-dried flask and cooled to -40 °C. A 2 M diethyl ether solution of *iso*-butylmagnesium bromide (0.50 mL, 1.0 mmol) was added dropwise and the mixture was allowed to warm to room temperature over ~1 h. After stirring for a further 2 h at room temperature the reaction was quenched with an aqueous $NaHCO_3$ solution (15 mL) and the mixture was extracted with ethyl acetate (3 × 15 mL). The combined organic extracts were dried ($MgSO_4$), filtered and the solvent was removed under reduced pressure. Purification by an SCX column followed by column chromatography on silica with a gradient elution from 0-40 % ethyl acetate in hexane gave a colourless oil (40 mg, 0.19 mmol, 39 %). **^{1}H NMR ($CDCl_3$, 500 MHz) δ$_H$** 8.34-8.40 (m, 1H), 6.98-7.01 (m, 1H), 6.94-6.98 (m, 1H), 3.91 (q, 1H, *J* =

7.0 Hz), 2.30 (s, 3H), 2.20-2.30 (m, 2H), 2.04-2.14 (m, 1H), 1.40 (d, 3H, *J* = 7.0 Hz), 0.82 (d, 3H, *J* = 6.6 Hz), 0.75 (d, 3H, *J* = 6.6 Hz); **^{13}C{^{1}H} NMR (CDCl$_3$, 125 MHz) δ_C** 209.8, 159.9, 149.2, 147.9, 123.0, 122.9, 55.4, 50.3, 24.2, 22.5, 22.3, 21.0, 16.1; **HRMS (ESI) m/z:** $[M+H]^+$ Calcd for $C_{13}H_{20}NO$ 206.1539; Found 206.1546; **ν_{max}** 2956, 1712, 1603, 1453, 1366, 1028, 825 cm^{-1}.

***N*-Benzyl 2-(4-methylpyridin-2-yl)propanamide, 3w**

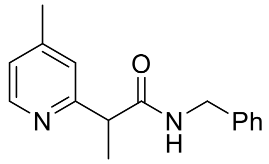

Made following general procedure D. Column chromatography on silica with a gradient elution from 40-100 % ethyl acetate in hexane followed by 10 % methanol in ethyl acetate gave the product which was further purified by an SCX column to give a yellow oil (101 mg, 0.40 mmol, 80 %). **^{1}H NMR (CDCl$_3$, 500 MHz) δ_H** 8.30-8.35 (m, 1H), 7.61 (broad s, 1H), 7.14-7.31 (m, 5H), 7.10-7.13 (m, 1H), 6.96-7.01 (m, 1H), 4.33-4.39 (m, 2H), 3.77 (q, 1H, *J* = 7.2 Hz), 2.32 (s, 3H), 1.58 (d, 3H, *J* = 7.2 Hz); **^{13}C{^{1}H} NMR (CDCl$_3$, 125 MHz) δ_C** 173.0, 160.6, 148.6, 148.2, 138.6, 128.4, 127.3, 127.0, 123.0, 122.9, 49.1, 43.3, 20.8, 18.4; **HRMS (ESI) m/z:** $[M+H]^+$ Calcd for $C_{16}H_{19}N_2O$ 255.1492; Found 255.1494; **ν_{max}** 3291, 2930, 1650, 1604, 1537, 1453, 1217, 730, 698 cm^{-1}.

Ethyl 2-(2-(4-methylpyridin-2-yl)propanamido)acetate, 3x

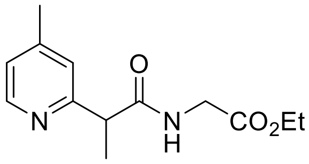

Made following general procedure D but substituting water for saturated $NaHCO_3$ solution in the aqueous workup. Column chromatography on silica with a gradient elution from 60-100 % ethyl acetate in hexane followed by 10 % methanol in ethyl acetate gave a yellow film (53 mg, 0.21 mmol, 42 %). **^{1}H NMR (CDCl$_3$, 500 MHz) δ_H** 8.34-8.41 (m, 1H), 7.68 (broad s, 1H), 7.04-7.08 (m, 1H), 6.94-7.00 (m, 1H), 4.13 (q, 2H, *J* = 7.1 Hz), 3.93-3.98 (m, 2H), 3.73 (q, 1H, *J* = 7.2 Hz), 3.00 (s, 3H), 1.54 (d, 3H, *J* = 7.2 Hz), 1.20 (t, 3H, *J* = 7.1 Hz); **^{13}C{^{1}H} NMR (CDCl$_3$, 125 MHz) δ_C** 173.3, 169.7, 160.3, 148.8, 148.3, 123.1, 123.0, 61.1, 48.9, 41.5, 20.9, 18.4, 14.0; **HRMS (ESI) m/z:** $[M+H]^+$ Calcd for $C_{13}H_{19}N_2O_3$ 251.1390; Found 251.1391; **ν_{max}** 3302, 2980, 1741, 1657, 1605, 1531, 1375, 1192, 1023 cm^{-1}.

***N*-Allyl 2-(4-methylpyridin-2-yl)propanamide, 3y**

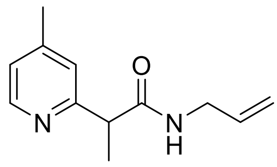

Made following general procedure C. Column chromatography on silica with a gradient elution from 40-100 % ethyl acetate in hexane followed by 10 % methanol in ethyl acetate gave an orange oil (61 mg, 0.30 mmol, 60 %). **^{1}H NMR ($CDCl_3$, 500 MHz) δ_H** 8.36-8.42 (m, 1H), 7.32 (broad s, 1H), 7.09-7.14 (m, 1H), 6.99-7.03 (m, 1H), 5.75-5.84 (m, 1H), 5.02-5.09 (m, 2H), 3.83-3.88 (m, 2H), 3.74 (q, 1H, *J* = 7.2 Hz), 2.34 (s, 3H), 1.57 (d, 3H, *J* = 7.2 Hz); **$^{13}C\{^1H\}$ NMR ($CDCl_3$, 125 MHz) δ_C** 173.0, 160.4, 148.6, 148.3, 134.2, 123.1, 123.0, 115.4, 49.0, 41.5, 20.9, 18.6; **HRMS (ESI) m/z:** $[M+H]^+$ Calcd for $C_{12}H_{17}N_2O$ 205.1335; Found 205.1342; **ν_{max}** 3291, 2931, 1642, 1603, 1535, 1452, 1214, 992, 915, 827 cm^{-1}.

2-(4-Methylpyridin-2-yl)-1-(morpholin-1-yl)propan-1-amide, 3z

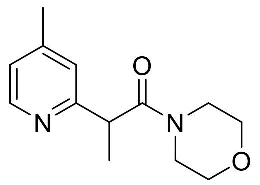

Made following general procedure D. Column chromatography on silica with a gradient elution from 40-100 % ethyl acetate in hexane followed by 10 % methanol in ethyl acetate gave a yellow oil (85 mg, 0.36 mmol, 73 %). **^{1}H NMR ($CDCl_3$, 500 MHz) δ_H** 8.25-8.31 (m, 1H), 7.05-7.09 (m, 1H), 6.90-6.94 (m, 1H), 4.03 (q, 1H, *J* = 6.9 Hz), 3.65-3.73 (m, 1H), 3.56-3.63 (m, 1H), 3.41-3.51 (m, 5H), 3.07-3.14 (m, 1H), 2.27 (s, 3H), 1.44 (d, 3H, *J* = 6.9 Hz); **$^{13}C\{^1H\}$ NMR ($CDCl_3$, 125 MHz) δ_C** 171.6, 161.2, 148.8, 148.1, 122.8, 121.9, 66.6, 46.1, 45.5, 42.5, 20.9, 18.4; **HRMS (ESI) m/z:** $[M+H]^+$ Calcd for $C_{13}H_{19}N_2O_2$ 235.1441; Found 235.1448; **ν_{max}** 3469, 2854, 1640, 1603, 1431, 1299, 1268, 1250, 1234, 1112, 1068, 1031, 841, 575 cm^{-1}.

2-(4-Methylpyridin-2-yl)-1-(pyrrolidin-1-yl)propan-1-amide, 3aa

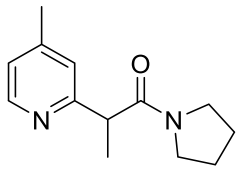

Made following general procedure C. Column chromatography on silica with a gradient elution from 60-100 % ethyl acetate in hexane followed by 10 % methanol in ethyl acetate gave a yellow oil (76 mg, 0.35 mmol, 70 %). **^{1}H NMR ($CDCl_3$, 500 MHz) δ_H** 8.19-8.28 (m, 1H), 7.06-7.15 (m, 1H), 6.82-6.90 (m, 1H), 3.91 (q, 1H, *J* = 7.0 Hz), 3.18-3.49 (m, 4H), 3.21 (s, 3H), 1.61-1.87 (m, 4H), 1.38 (d, 3H, *J* = 7.0 Hz); **$^{13}C\{^1H\}$ NMR ($CDCl_3$, 125 MHz) δ_C** 171.5, 161.2, 148.5, 147.8, 122.7, 122.0, 47.3, 46.4, 45.8, 25.9,

24.0, 20.8, 18.2; **HRMS (ESI) m/z:** $[M+H]^+$ Calcd for $C_{13}H_{18}N_2O$ 219.1492; Found 219.1490; $\mathbf{\nu_{max}}$ 3446, 2972, 2873, 1625, 1603, 1429 cm^{-1}.

2-(4-Methylpyridin-2-yl)-1-(indolin-1-yl)propan-1-amide, 3ab

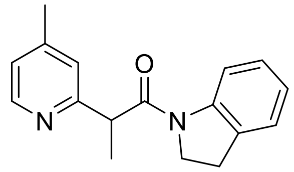

Made following general procedure D. Column chromatography on silica with a gradient elution from 40-100 % ethyl acetate in hexane followed by 10 % methanol in ethyl acetate gave the product which was further purified by an SCX column to give a brown crystalline solid (100 mg, 0.38 mmol, 75 %). **Mp** 105-106 °C (EtOAc/pentane); **^{1}H NMR ($CDCl_3$, 500 MHz) δ_H** 8.34-8.40 (m, 1H), 8.29-8.33 (m, 1H), 7.15-7.24 (m, 2H), 7.10-7.15 (m, 1H), 6.95-7.01 (m, 2H), 4.10-4.19 (m, 2H), 3.94-4.02 (m, 1H), 3.07-3.17 (m, 1H), 2.97-3.07 (m, 1H), 2.31 (s, 3H), 1.58 (d, 3H, *J* = 6.9 Hz); **^{13}C{^{1}H} NMR ($CDCl_3$, 125 MHz) δ_C** 171.4, 160.6, 148.9, 148.4, 143.2, 131.4, 127.4, 124.4, 123.7, 123.2, 122.1, 117.2, 48.6, 48.0, 27.9, 21.0, 18.6; **HRMS (ESI) m/z:** $[M+H]^+$ Calcd for $C_{17}H_{19}N_2O$ 267.1492; Found 267.1495; $\mathbf{\nu_{max}}$ 2930, 1651, 1599, 1558, 1482, 1460, 1407, 1341, 1286, 1261, 1063, 839, 756 cm^{-1}.

References

1. Duric, S.; Tzschucke, C. C. *Org. Lett*, 2011, **13**, 2310-2313.
2. Diemer, V.; Chaumeil, H.; Defoin, A.; Fort, A.; Boeglin, A.; Carré, C. *Eur. J. Org. Chem*, 2008, 1767-1776.
3. Fillion, E.; Fishlock, D.; Wilsily, A.; Goll, J. M. *J. Org. Chem.* **2005**, *70*, 1316-1327.
4. Ramachary, D. B.; Kishor, M.; Reddy, Y. V. *Eur. J. Org. Chem*, 2008, 975-993.
5. Frost, C. G.; Penrose, S. D.; R. Gleave, R. *Synthesis*, 2009, 627-635.
6. Wright, A. D.; Haslego M. L.; Smith, F. X. *Tetrahedron Lett*, 1979, 2325-2326.
7. Frost, C. G.; Penrose, S. D.; Lambshead, K.; Raithby, P. R.; Warren, J. E.; Gleave, R. *Org. Lett*, 2007, **9**, 2119-2122.
8. Londregan, A. T.; Burford, K.; Conn, E. L.; Hesp, K. D. *Org. Lett*. **2014**, *16*, 3336–3339.
9. Kawanishi, R.; Phongphane, K.; Iwasa, S.; Shibatomi, K. *Chem. Eur. J.* **2019**, *25*, 7453-7456.
10. Allen, A. D.; Fedorov, A. V.; Tidwell, T. T.; Vukovic, S. *J. Am. CHem. Soc.* **2004**, *126*, 15777-15783.

Spectra of Novel Compounds

500 MHz ^{1}H NMR of **3a** in $CDCl_3$

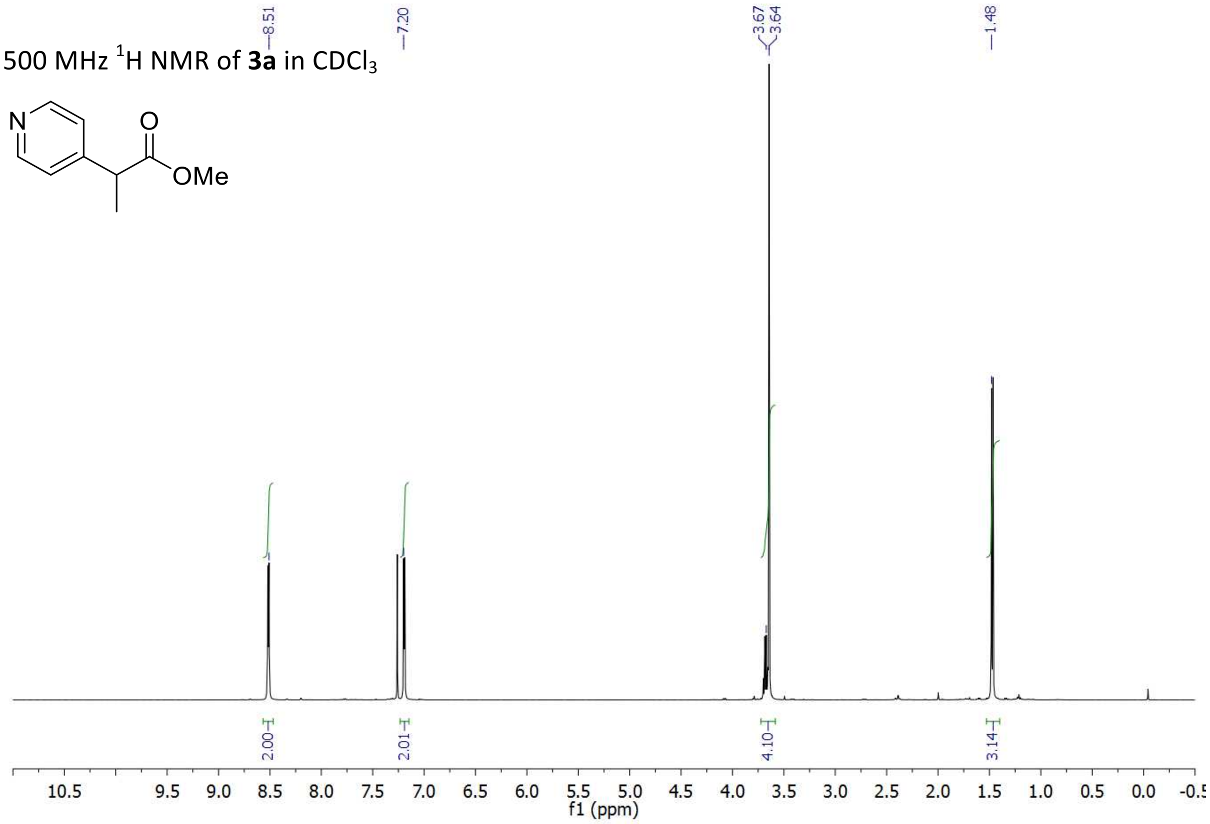

125 MHz $^{13}C\{^{1}H\}$ NMR of **3a** in $CDCl_3$

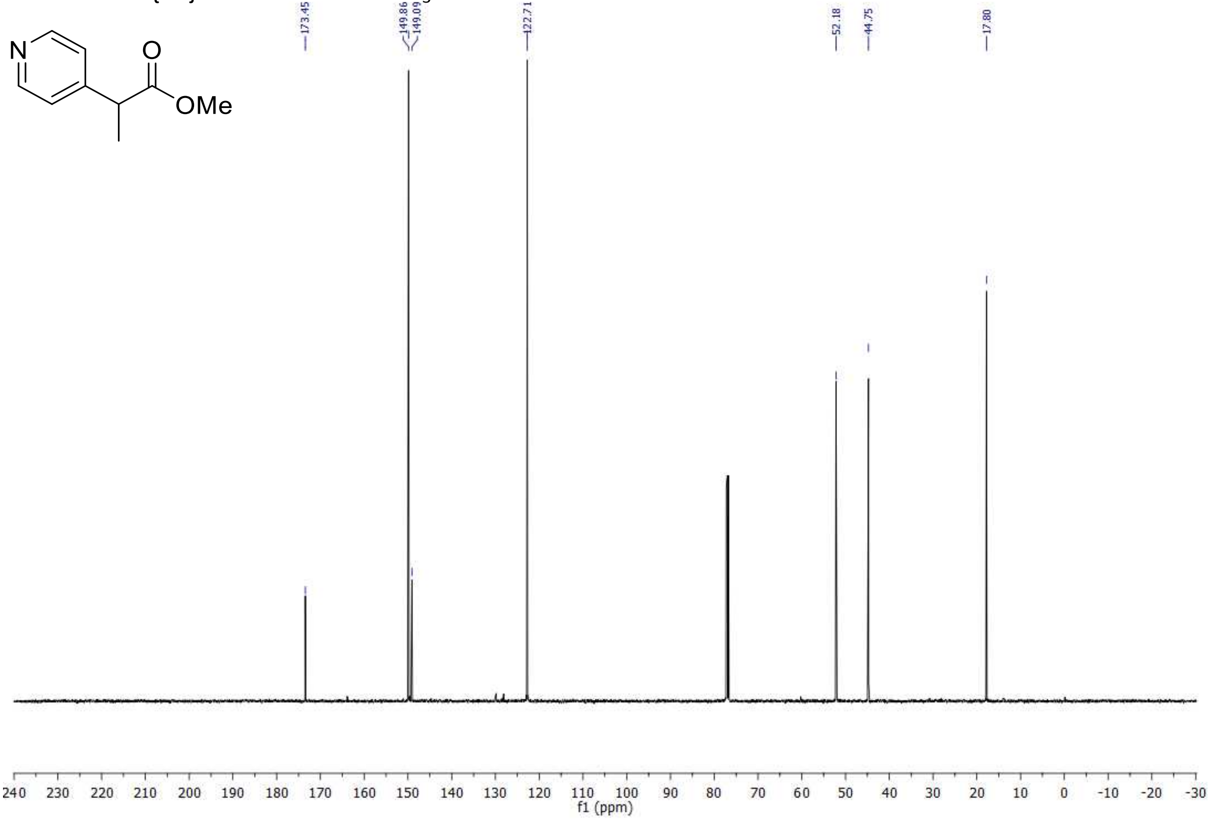

500 MHz ^{1}H NMR of **3b** in $CDCl_3$

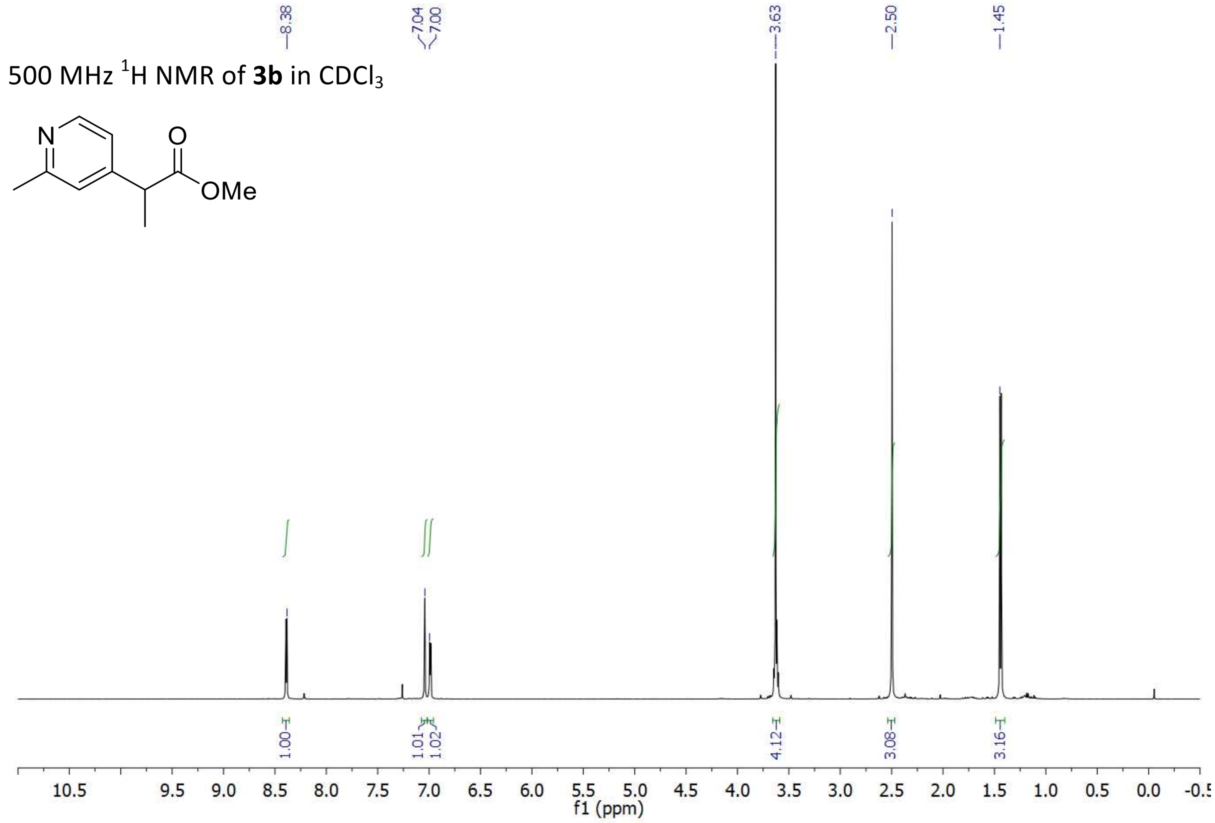

125 MHz $^{13}C\{^{1}H\}$ NMR of **3b** in $CDCl_3$

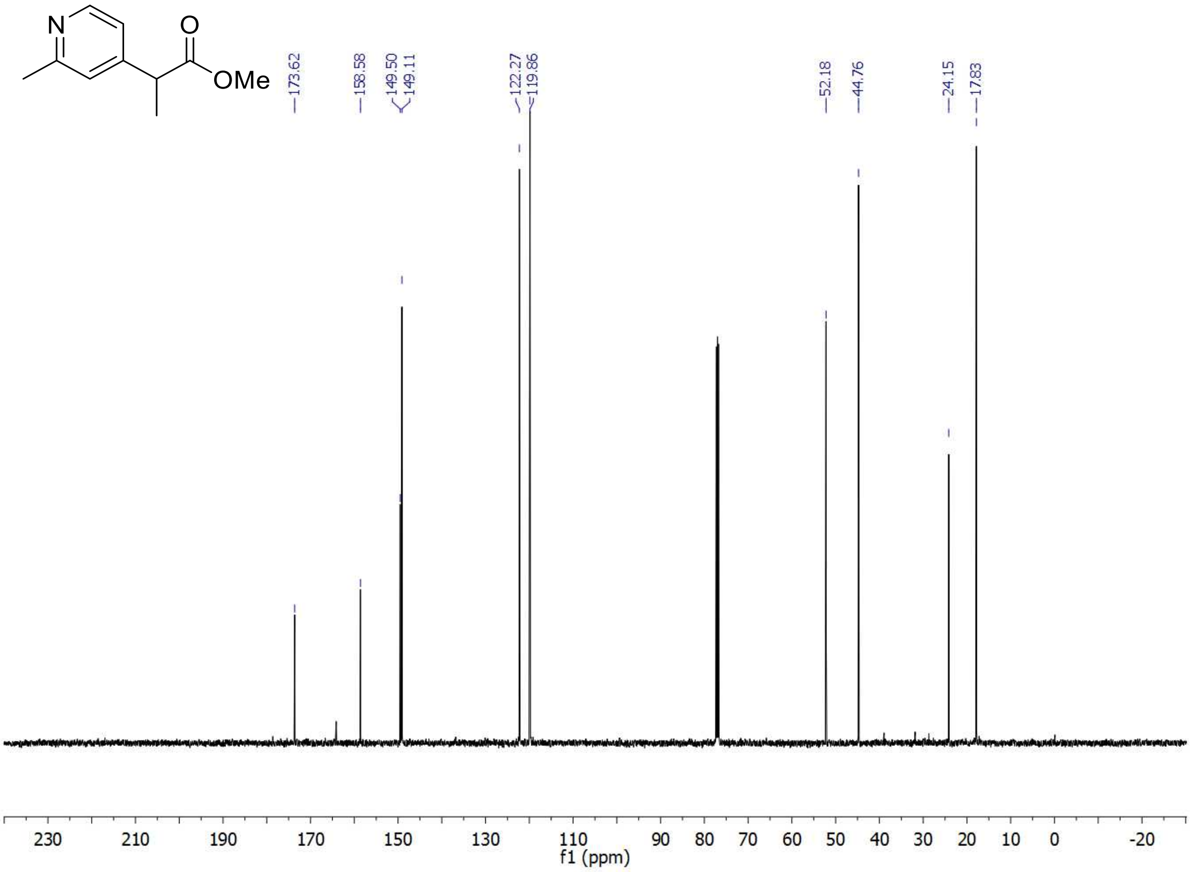

500 MHz ^{1}H NMR of **3c** in $CDCl_3$

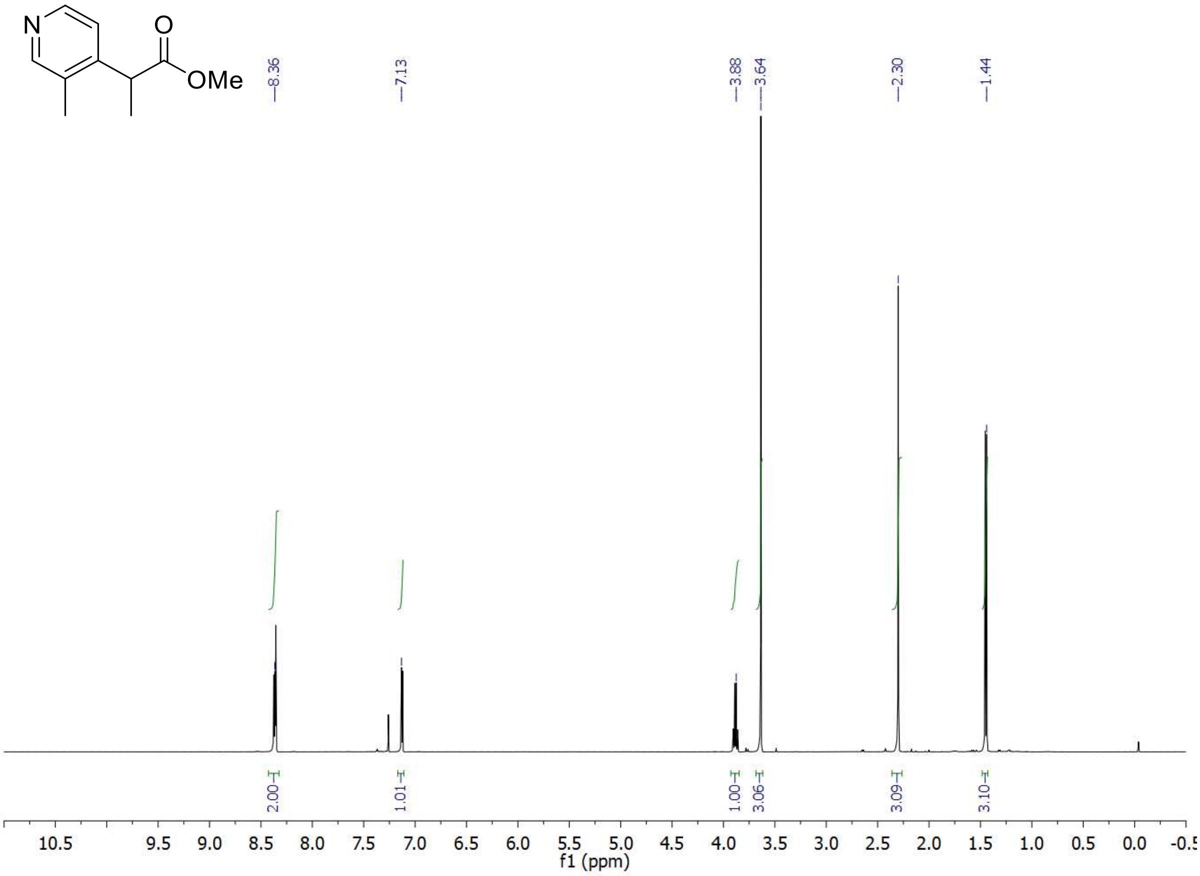

125 MHz $^{13}C\{^{1}H\}$ NMR of **3c** in $CDCl_3$

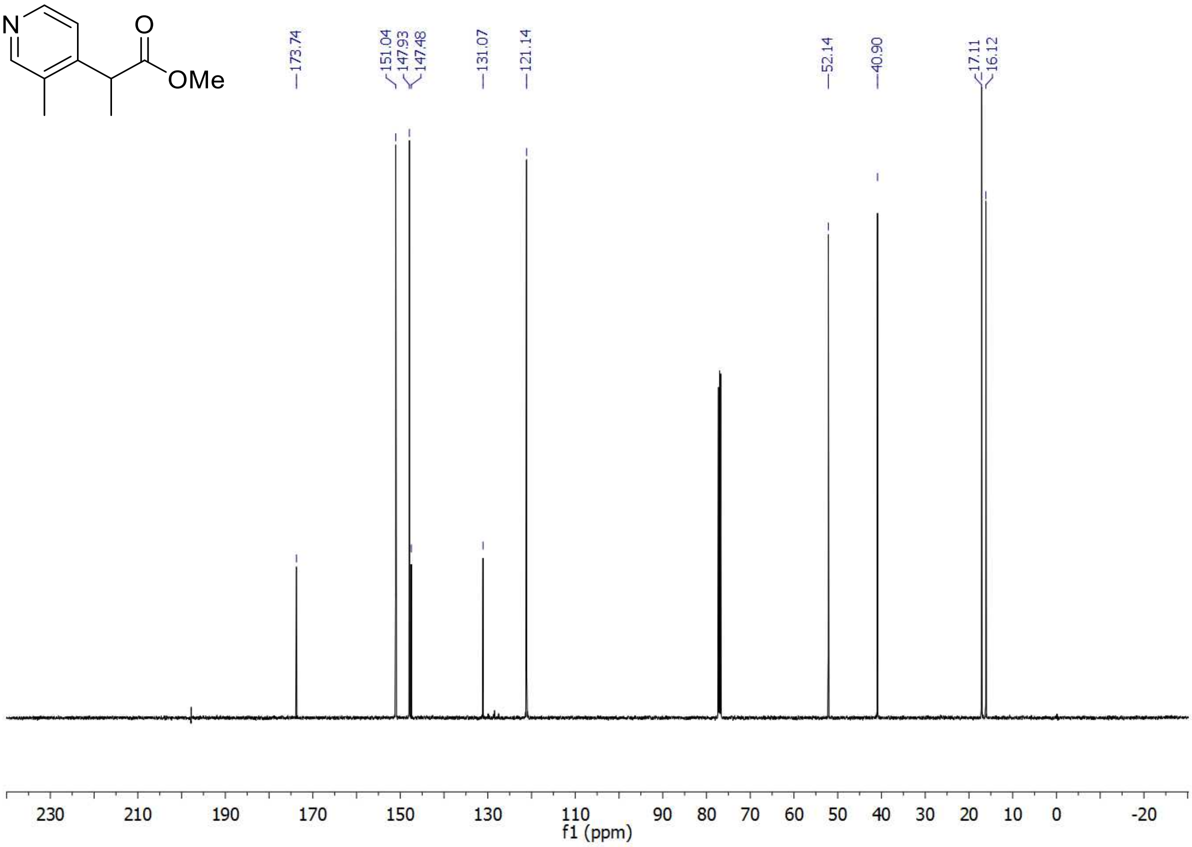

500 MHz ^{1}H NMR of **3d** in $CDCl_3$

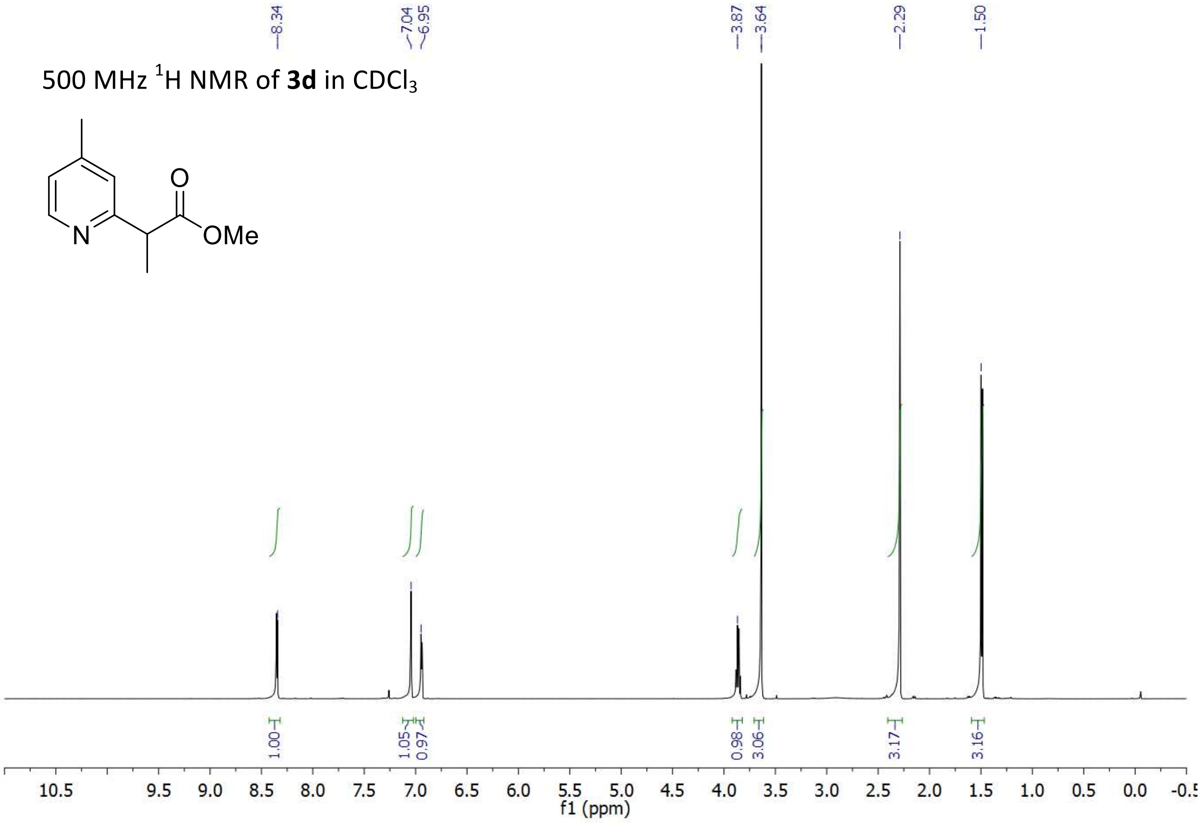

125 MHz $^{13}C\{^{1}H\}$ NMR of **3d** in $CDCl_3$

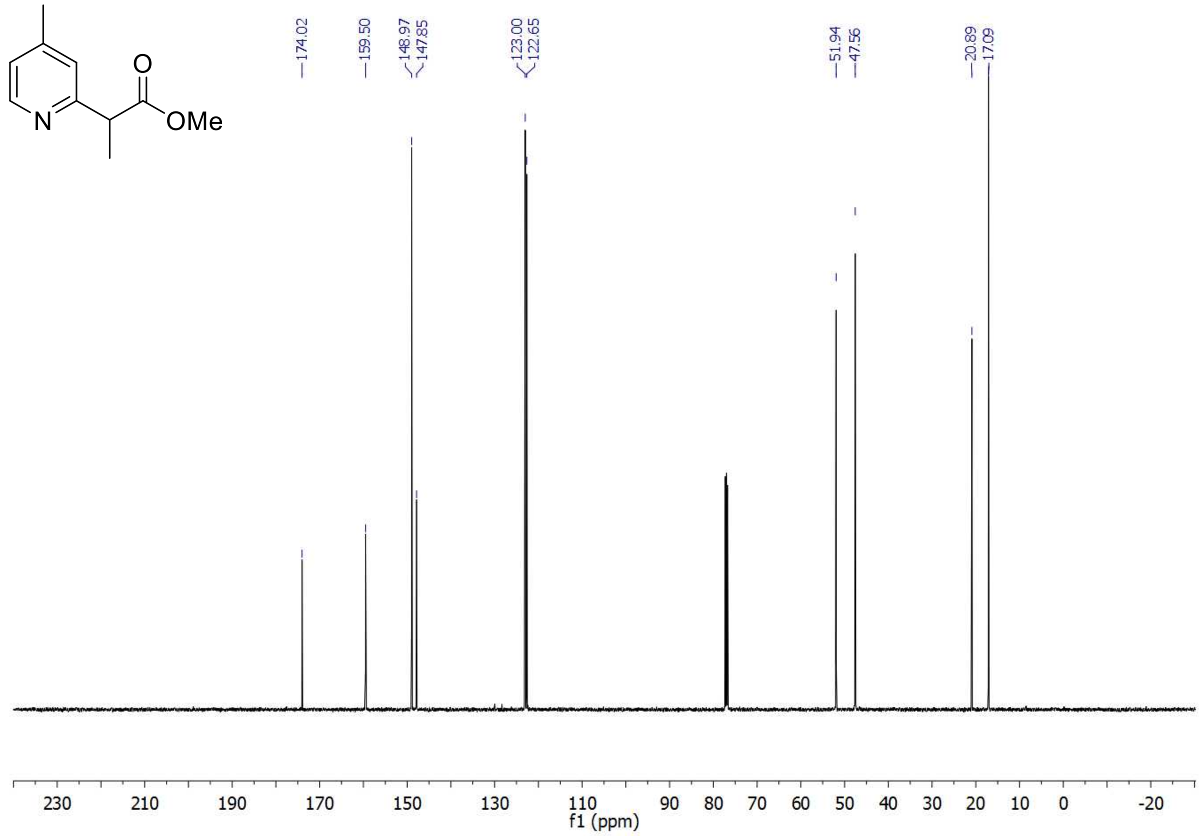

500 MHz ^{1}H NMR of **3e** in $CDCl_3$

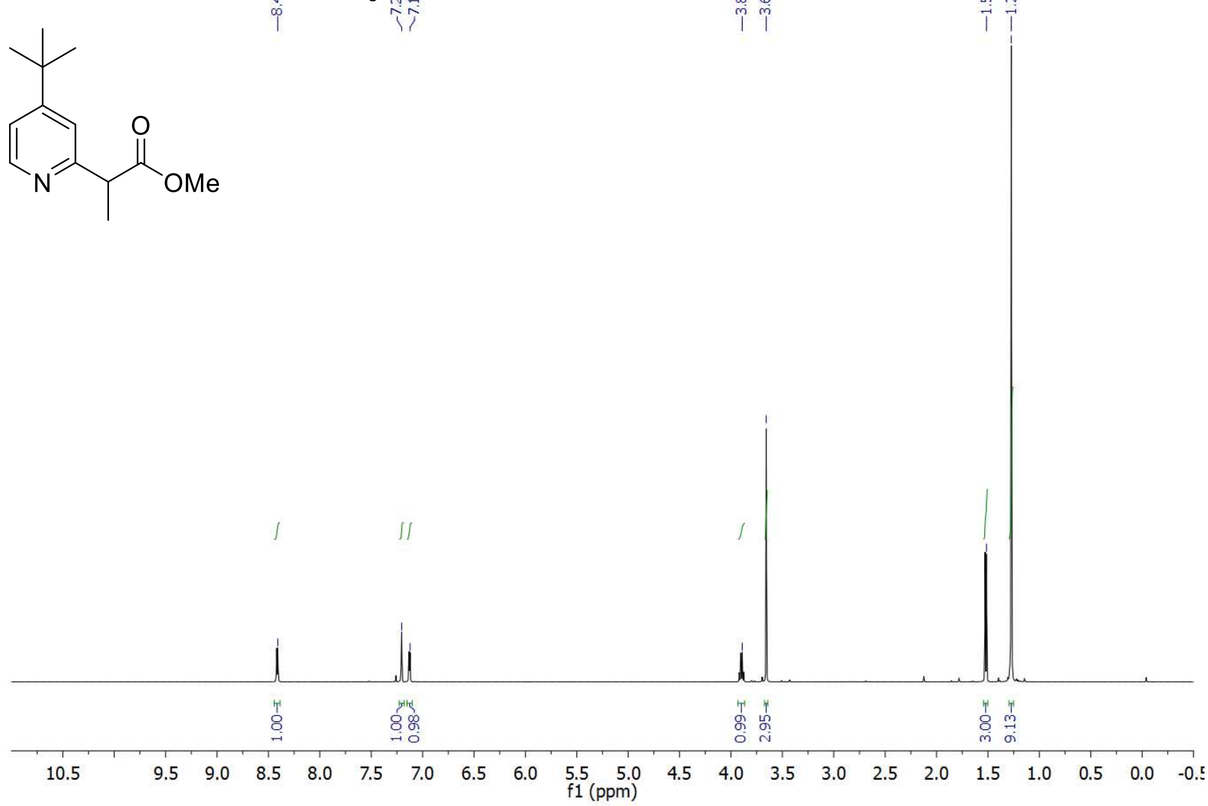

125 MHz $^{13}C\{^{1}H\}$ NMR of **3e** in $CDCl_3$

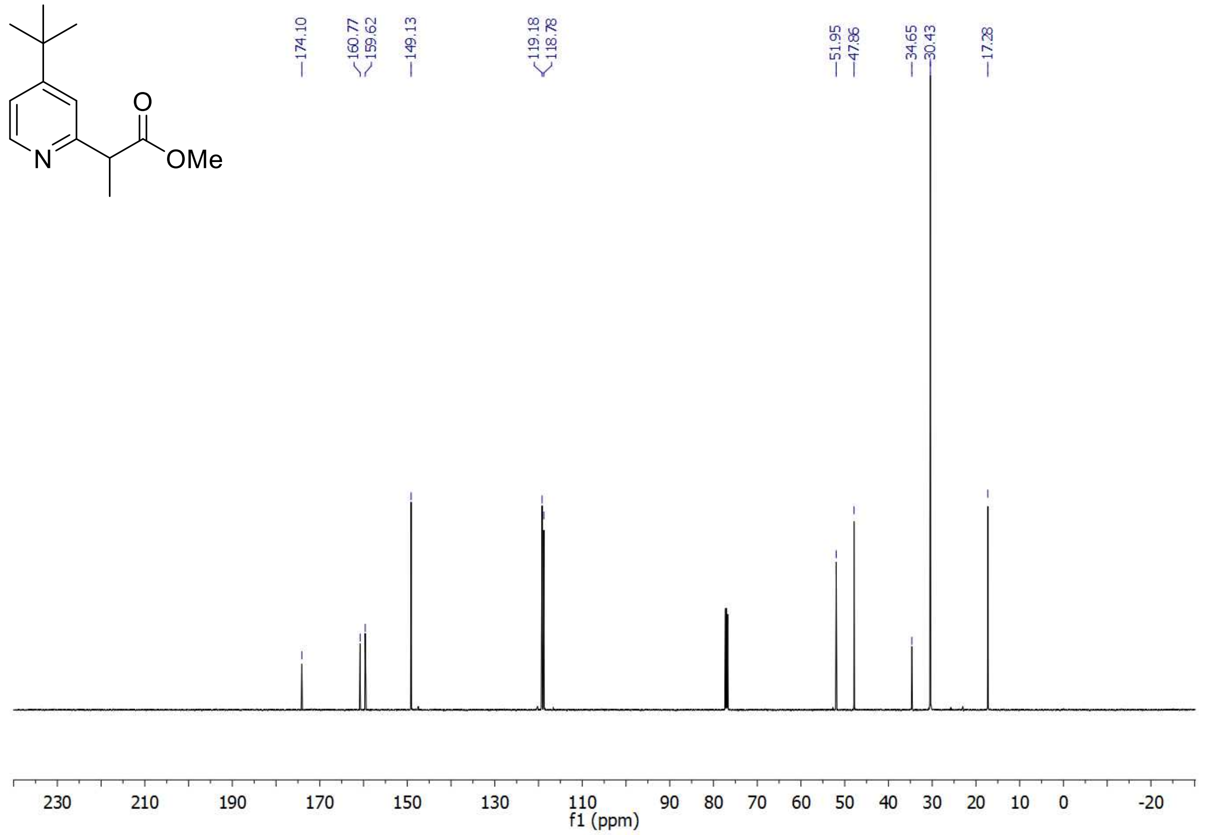

500 MHz ^{1}H NMR of **3f** in $CDCl_3$

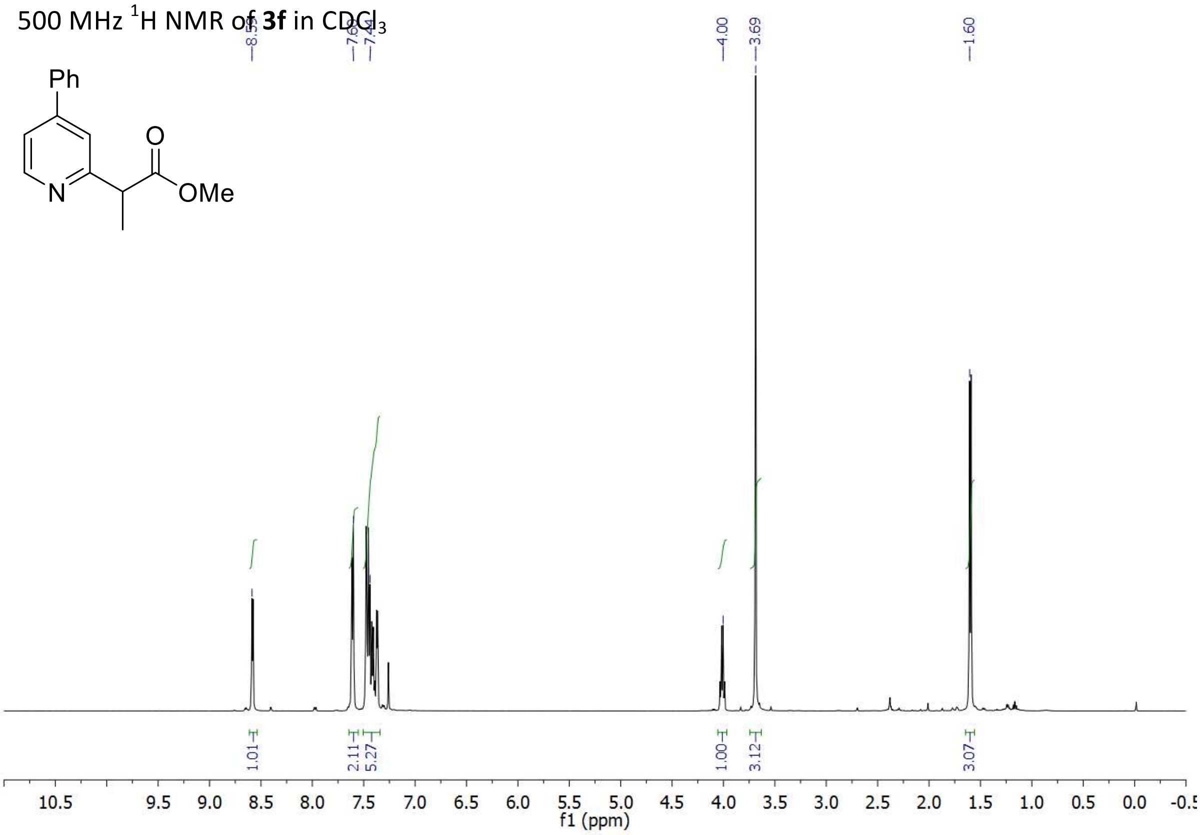

125 MHz $^{13}C\{^{1}H\}$ NMR of **3f** in $CDCl_3$

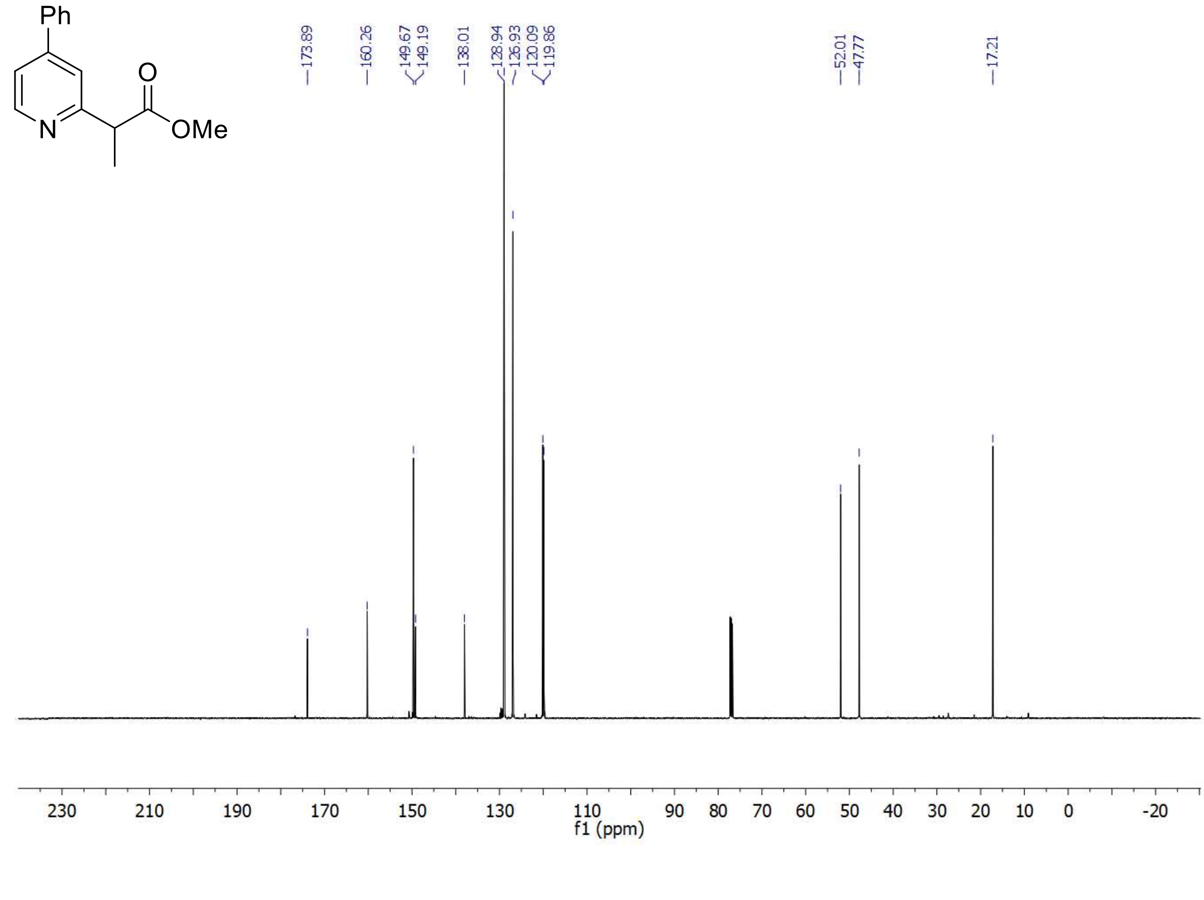

500 MHz ^{1}H NMR of **3g** in $CDCl_3$

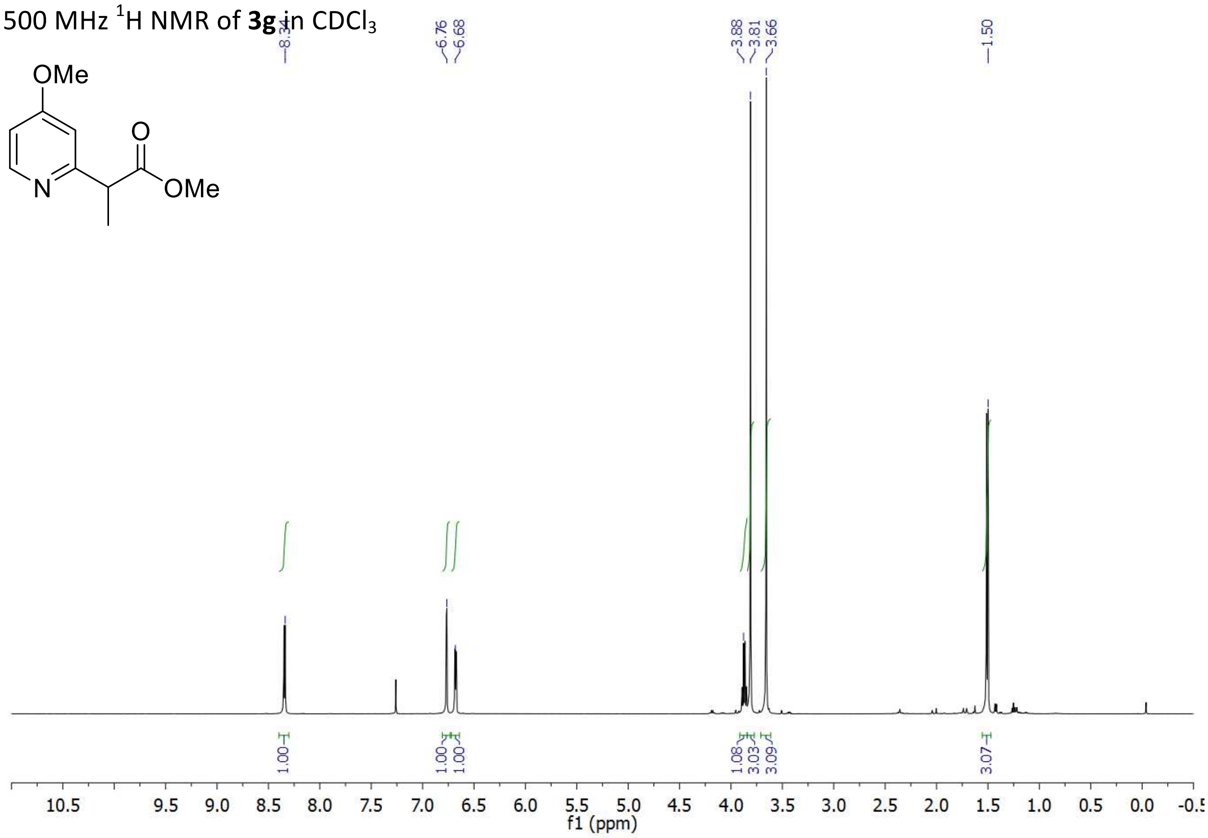

125 MHz $^{13}C\{^{1}H\}$ NMR of **3g** in $CDCl_3$

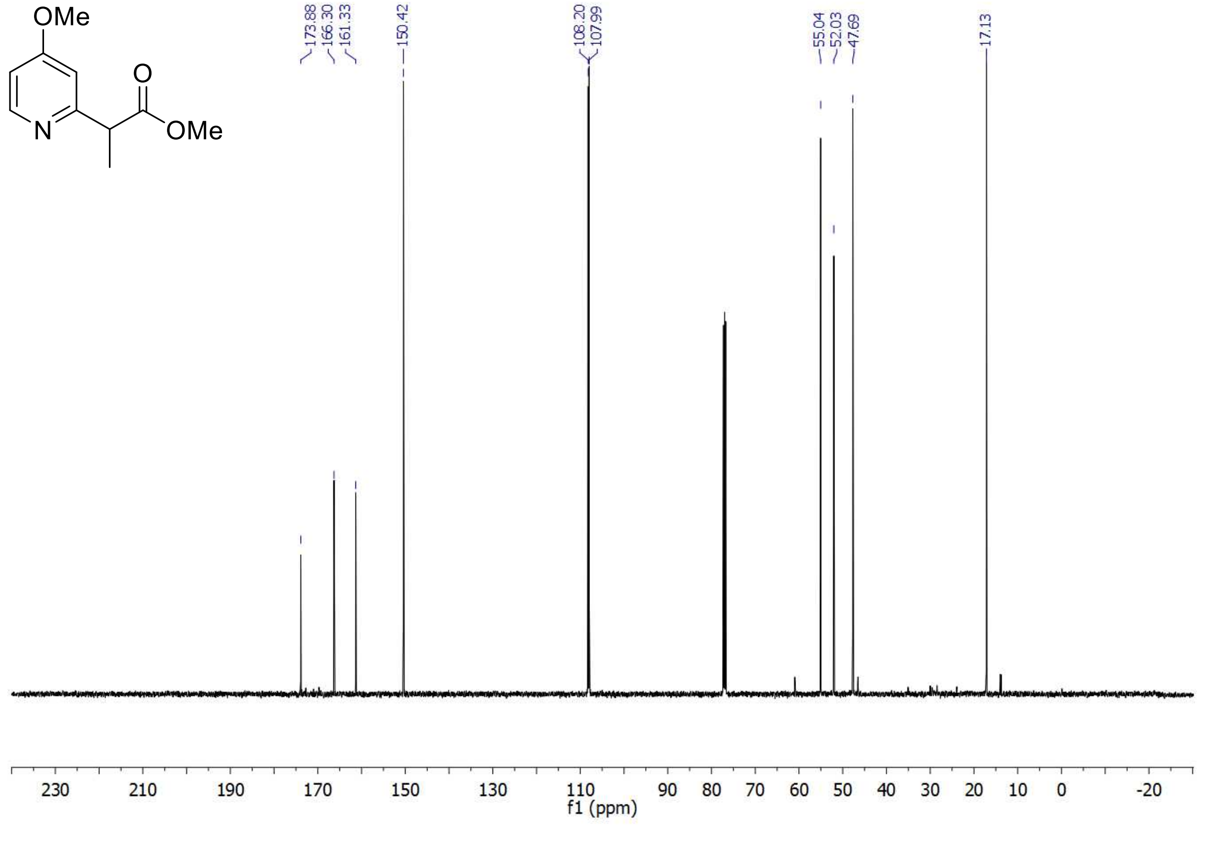

300 MHz ^{1}H NMR of **3h** in $CDCl_3$

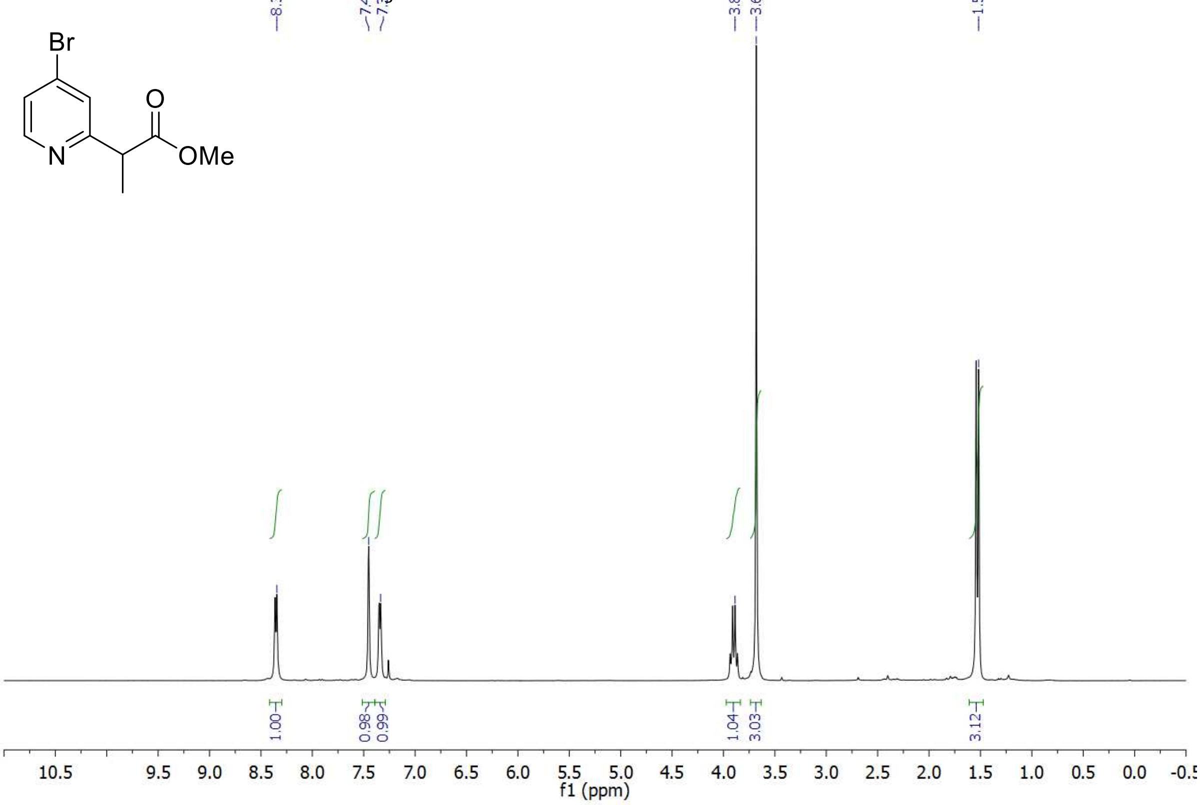

125 MHz $^{13}C\{^{1}H\}$ NMR of **3h** in $CDCl_3$

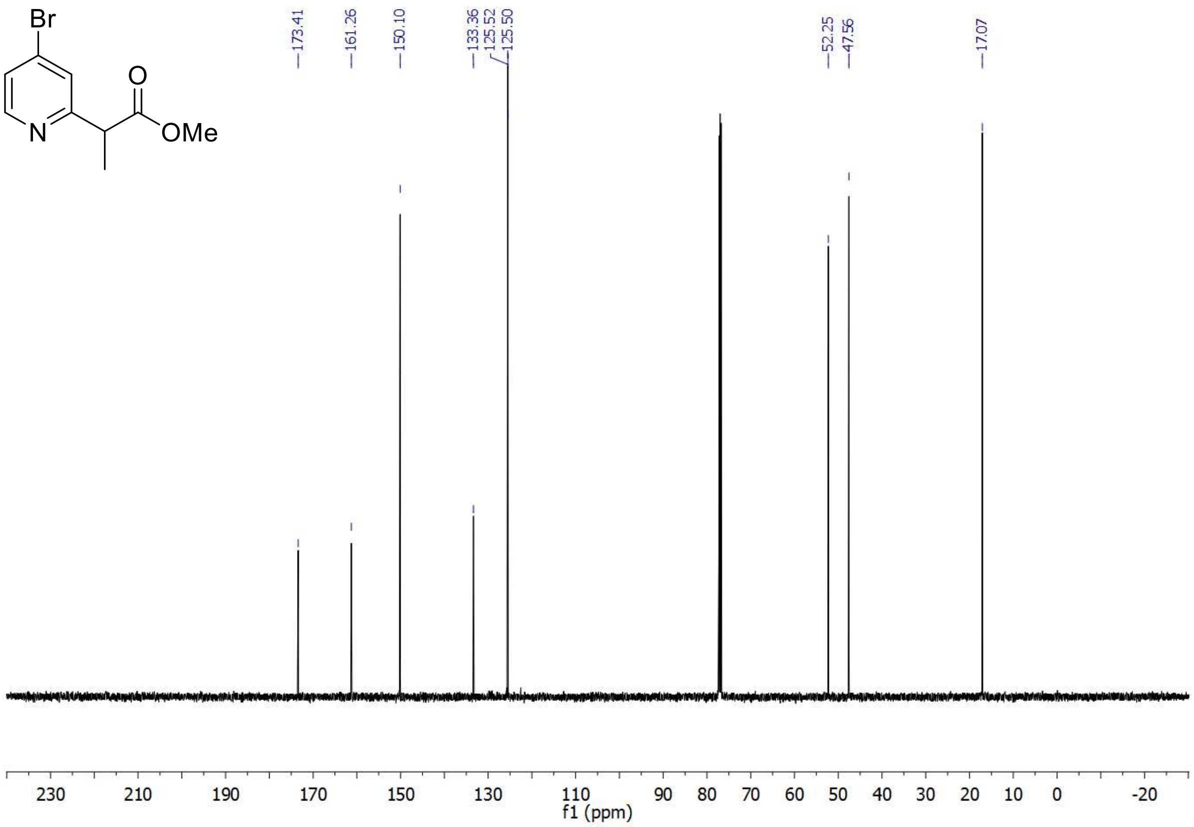

500 MHz ^{1}H NMR of **3j (2-isomer)** in $CDCl_3$

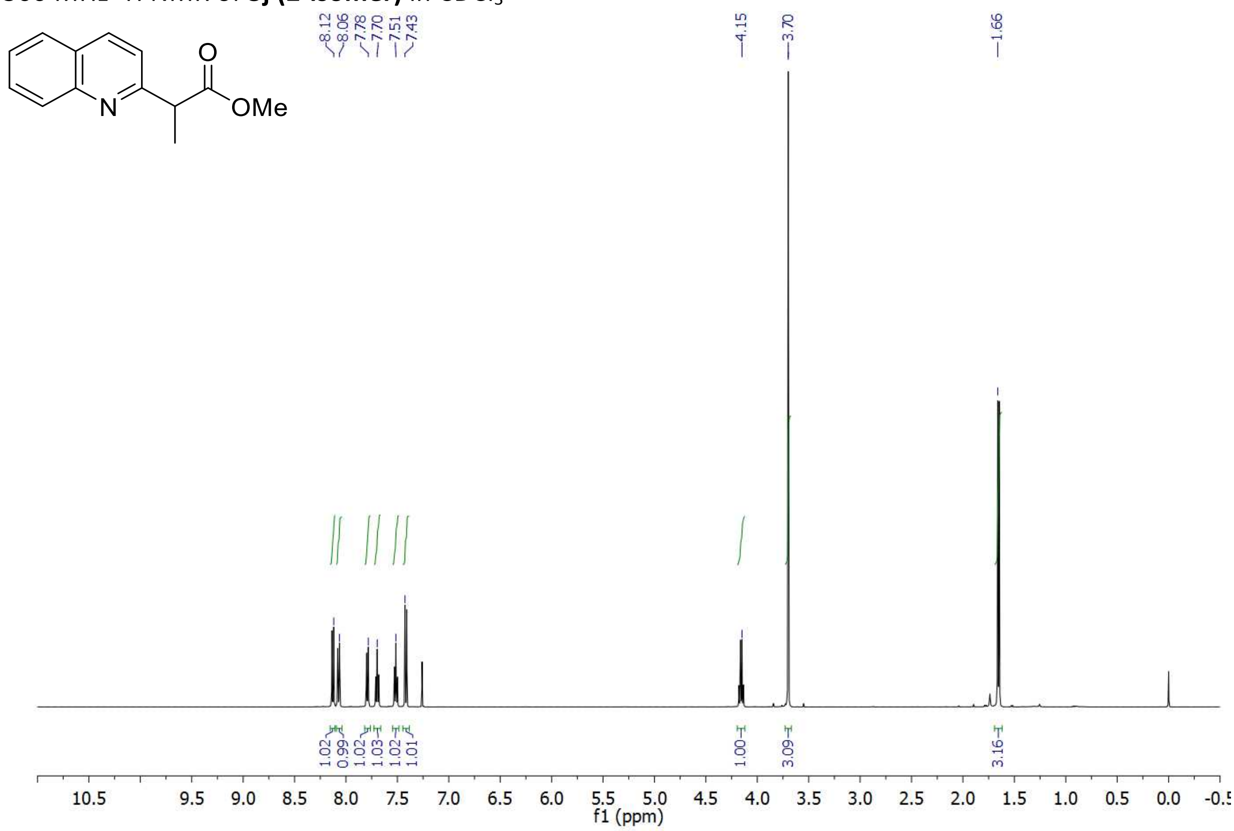

125 MHz $^{13}C\{^{1}H\}$ NMR of **3j (2-isomer)** in $CDCl_3$

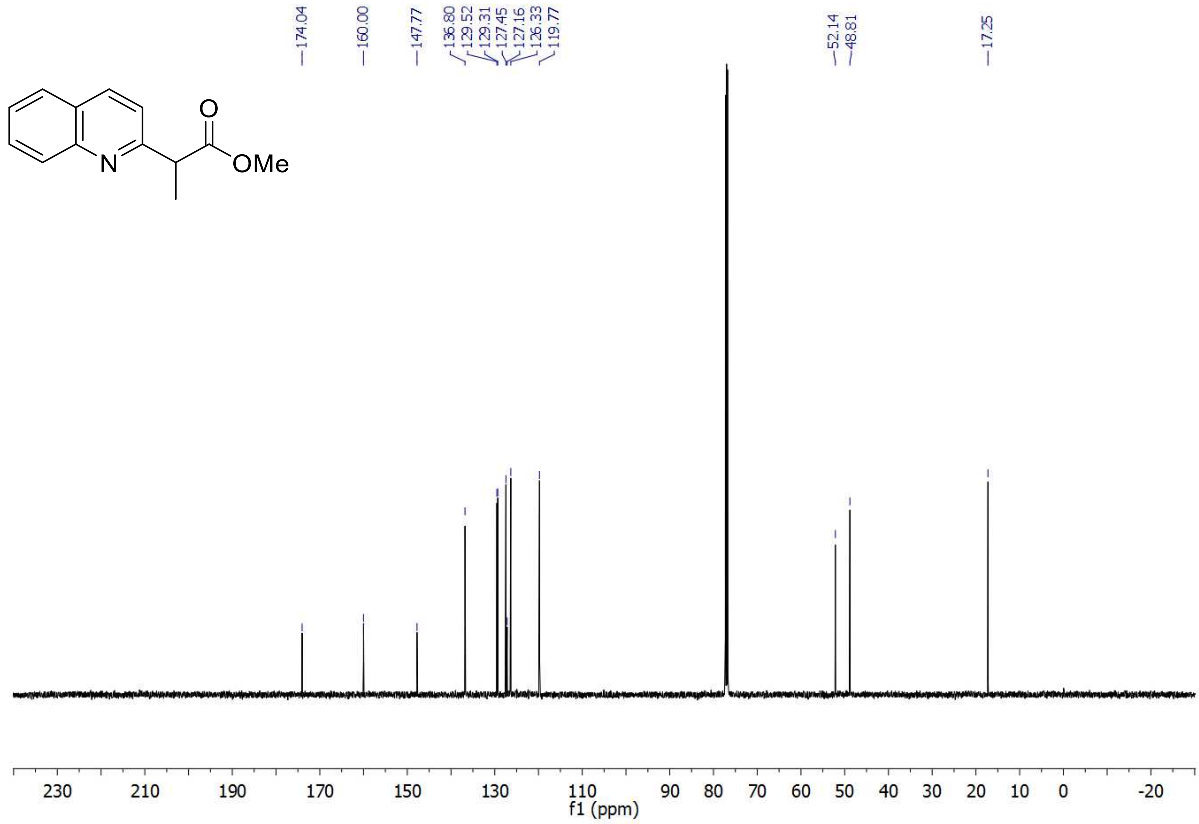

500 MHz ^{1}H NMR of **3i** in $CDCl_3$

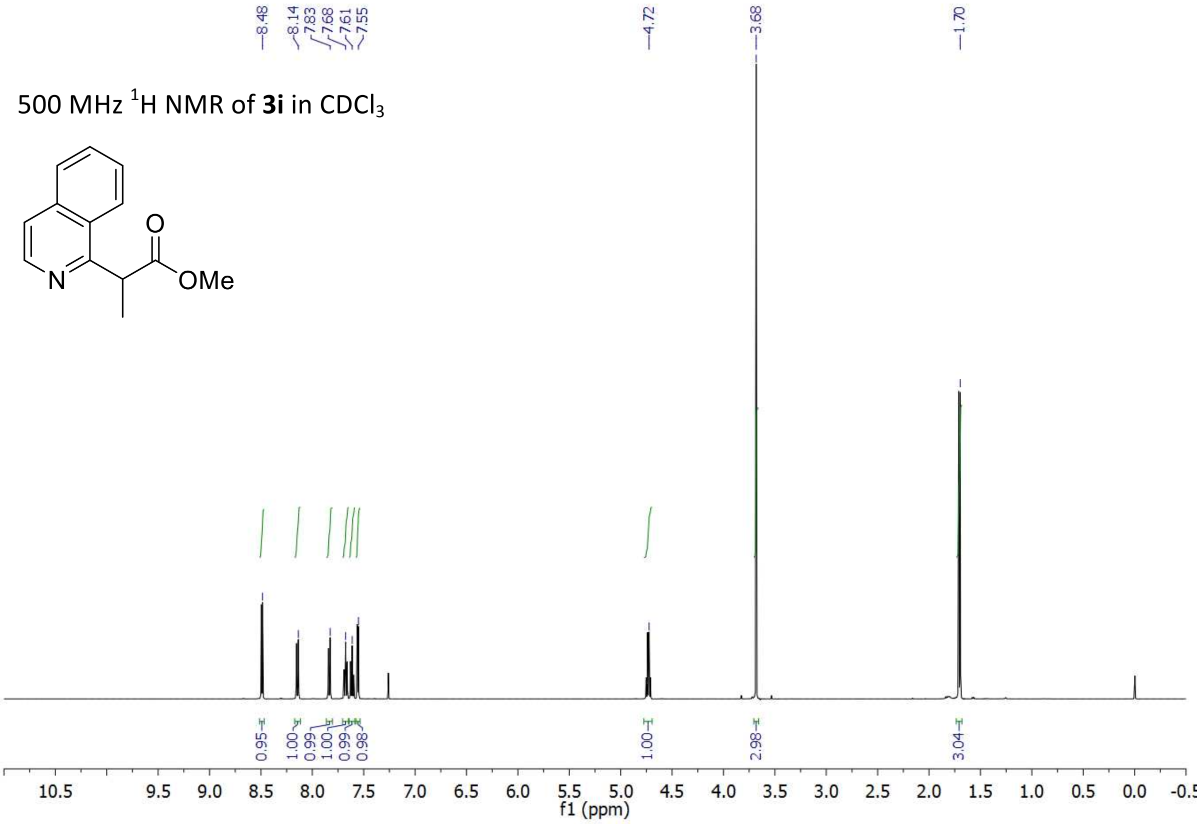

125 MHz $^{13}C\{^{1}H\}$ NMR of **3i** in $CDCl_3$

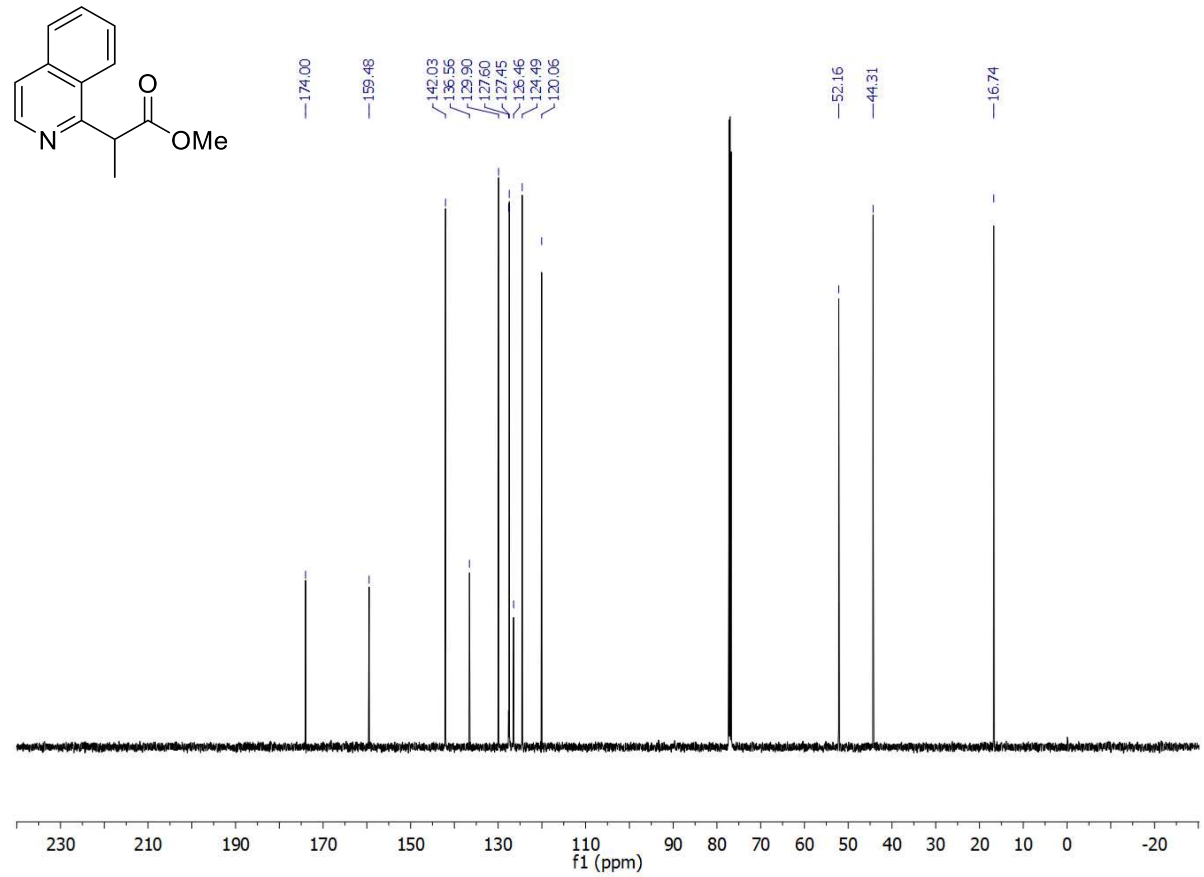

500 MHz ^{1}H NMR of **3j (4-isomer)** in $CDCl_3$

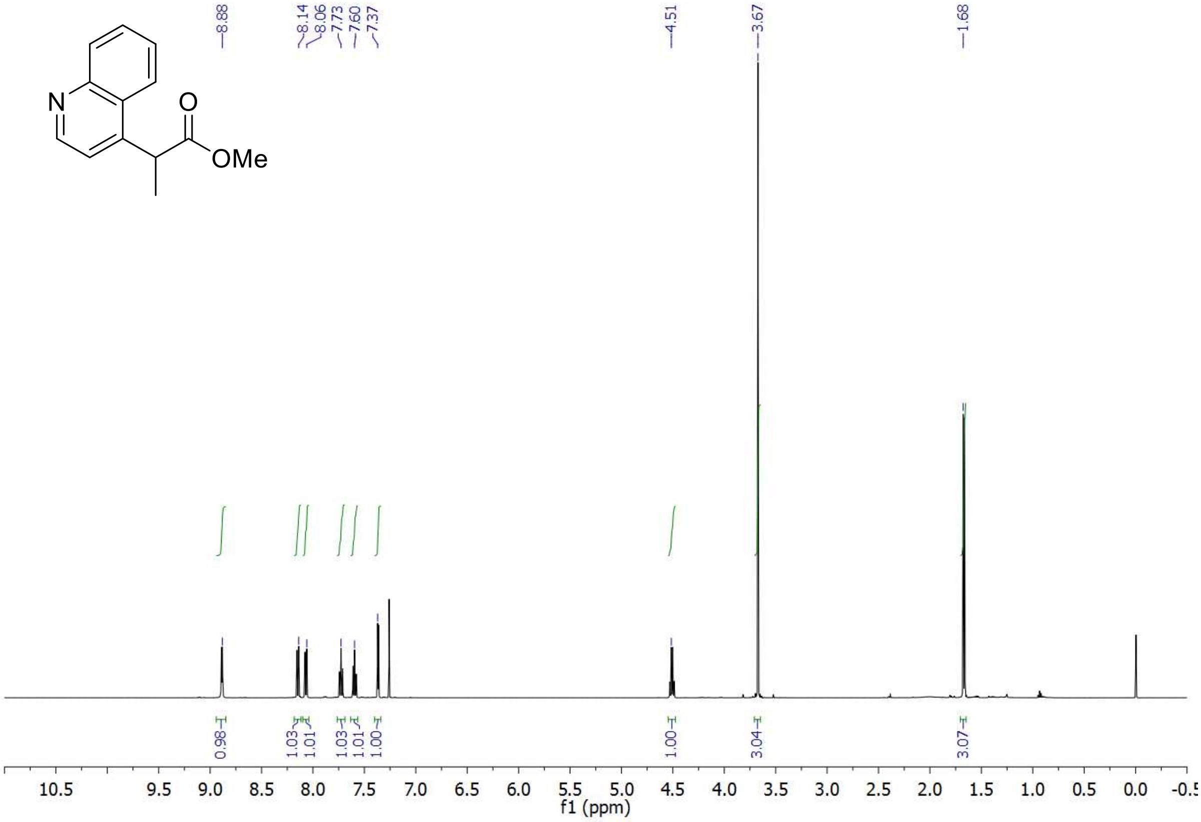

125 MHz $^{13}C\{^{1}H\}$ NMR of **3j (4-isomer)** in $CDCl_3$

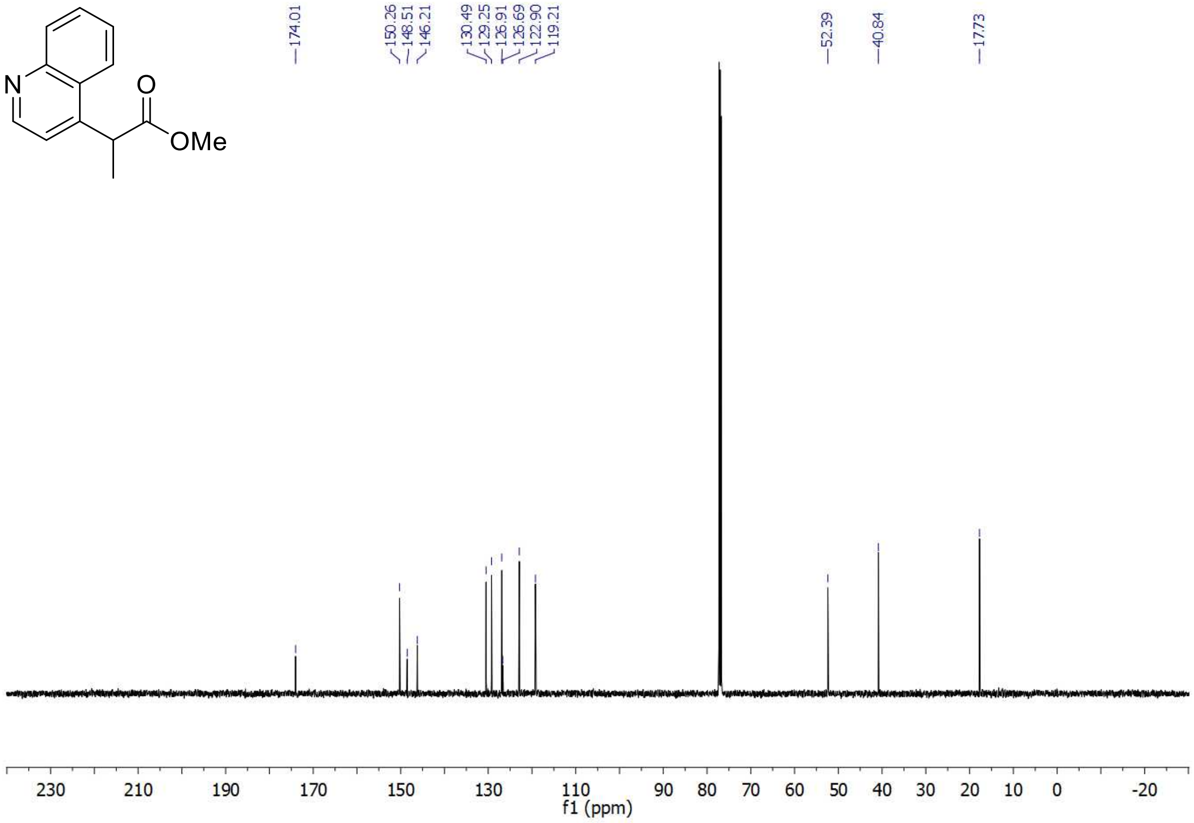

500 MHz ^{1}H NMR of **3k** in $CDCl_3$

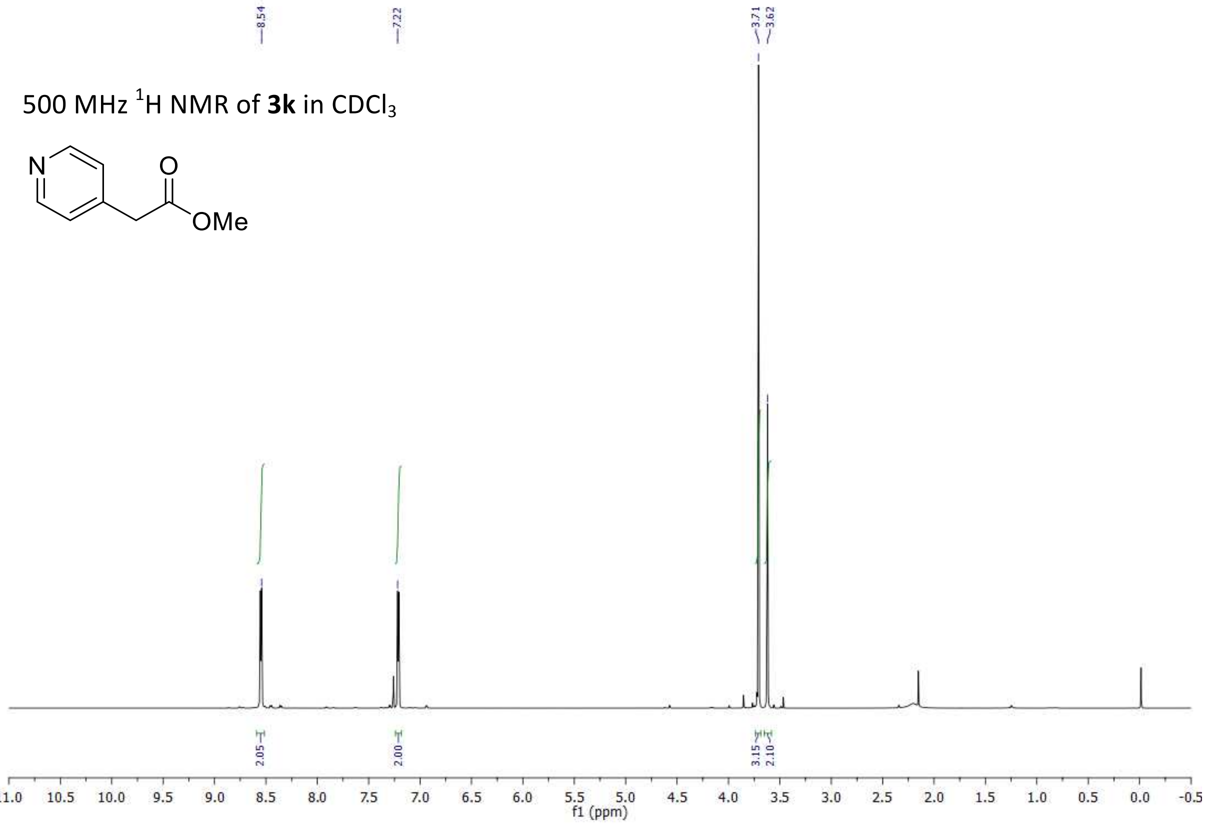

125 MHz $^{13}C\{^{1}H\}$ NMR of **3k** in $CDCl_3$

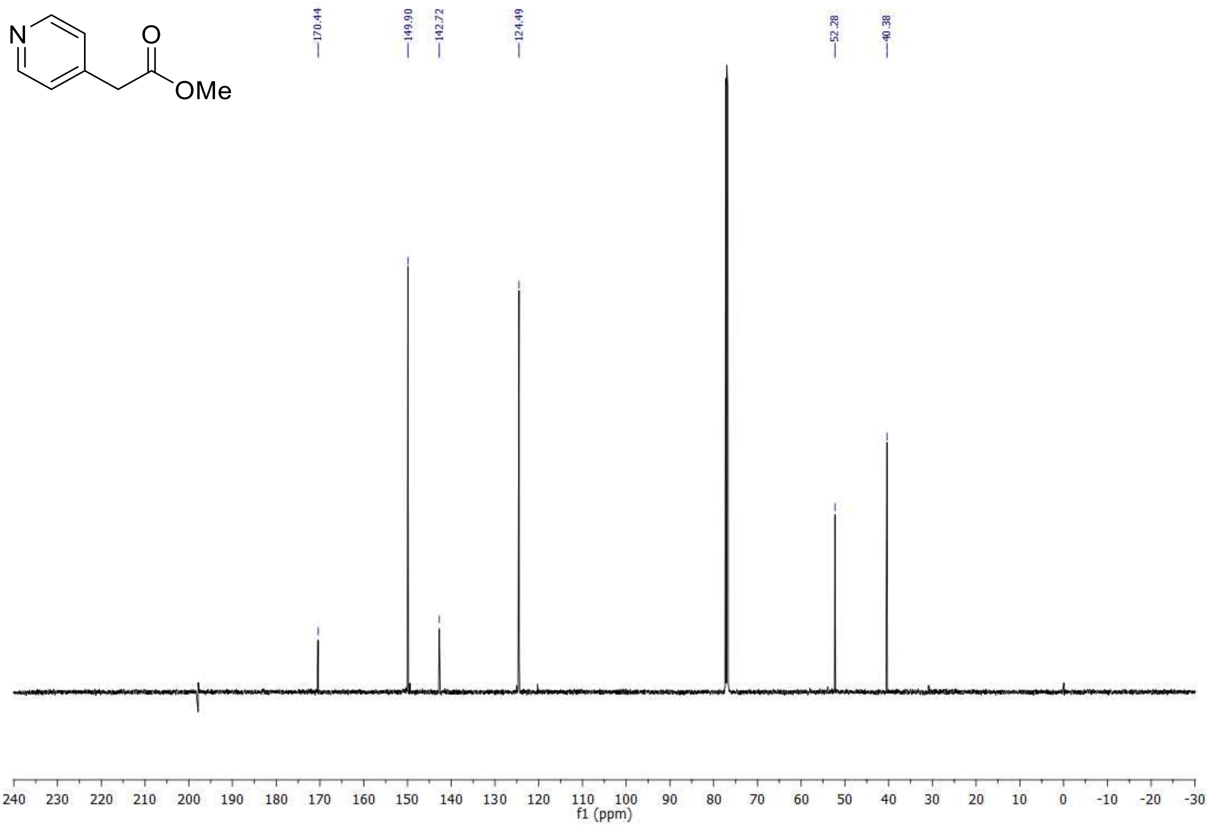

500 MHz ^{1}H NMR of **3l** in $CDCl_3$

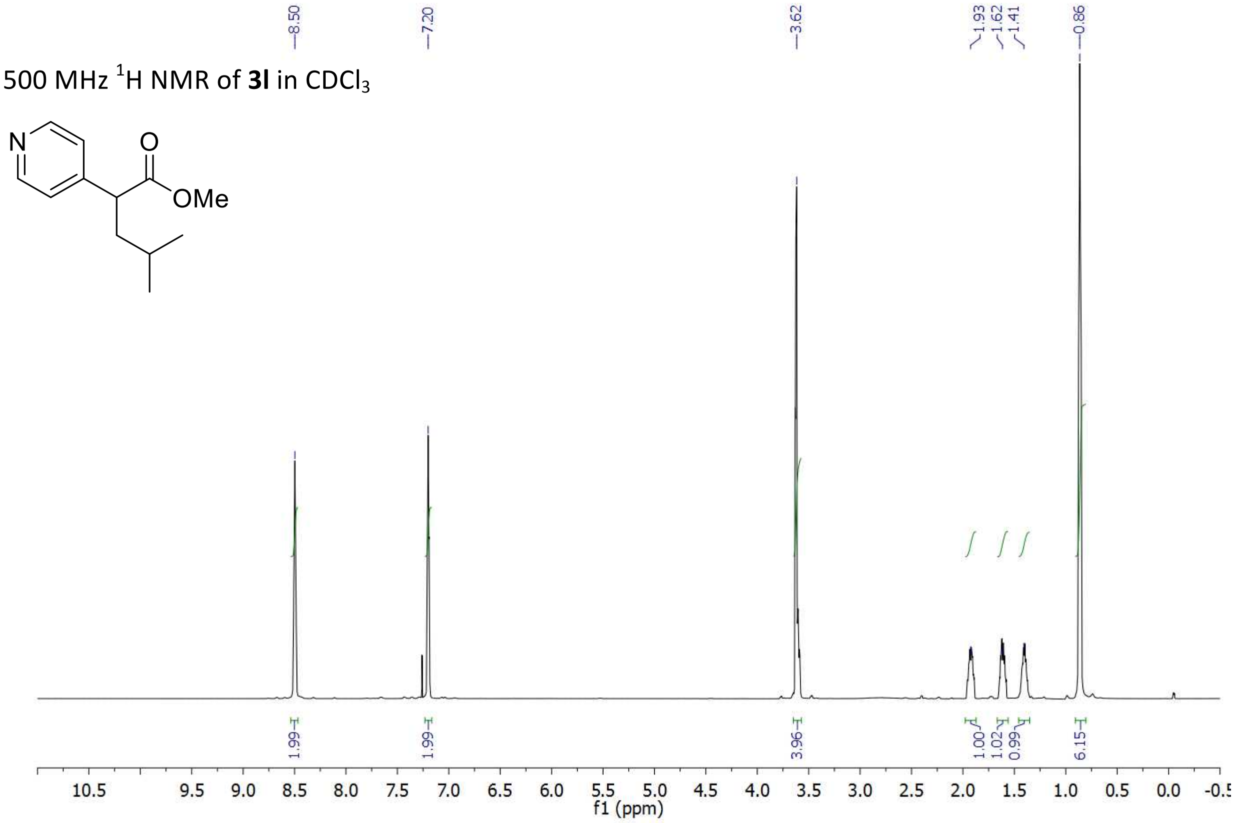

125 MHz $^{13}C\{^1H\}$ NMR of **3l** in $CDCl_3$

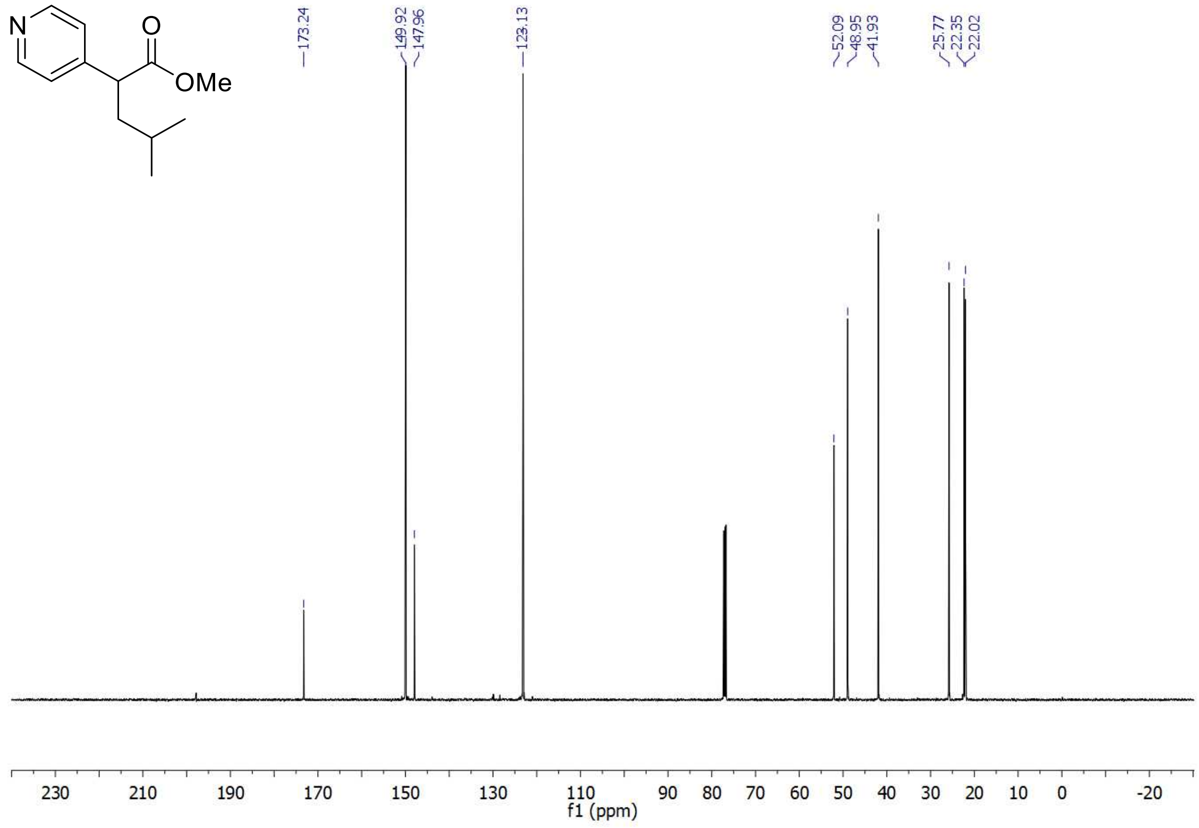

500 MHz ^{1}H NMR of **3m** in $CDCl_3$

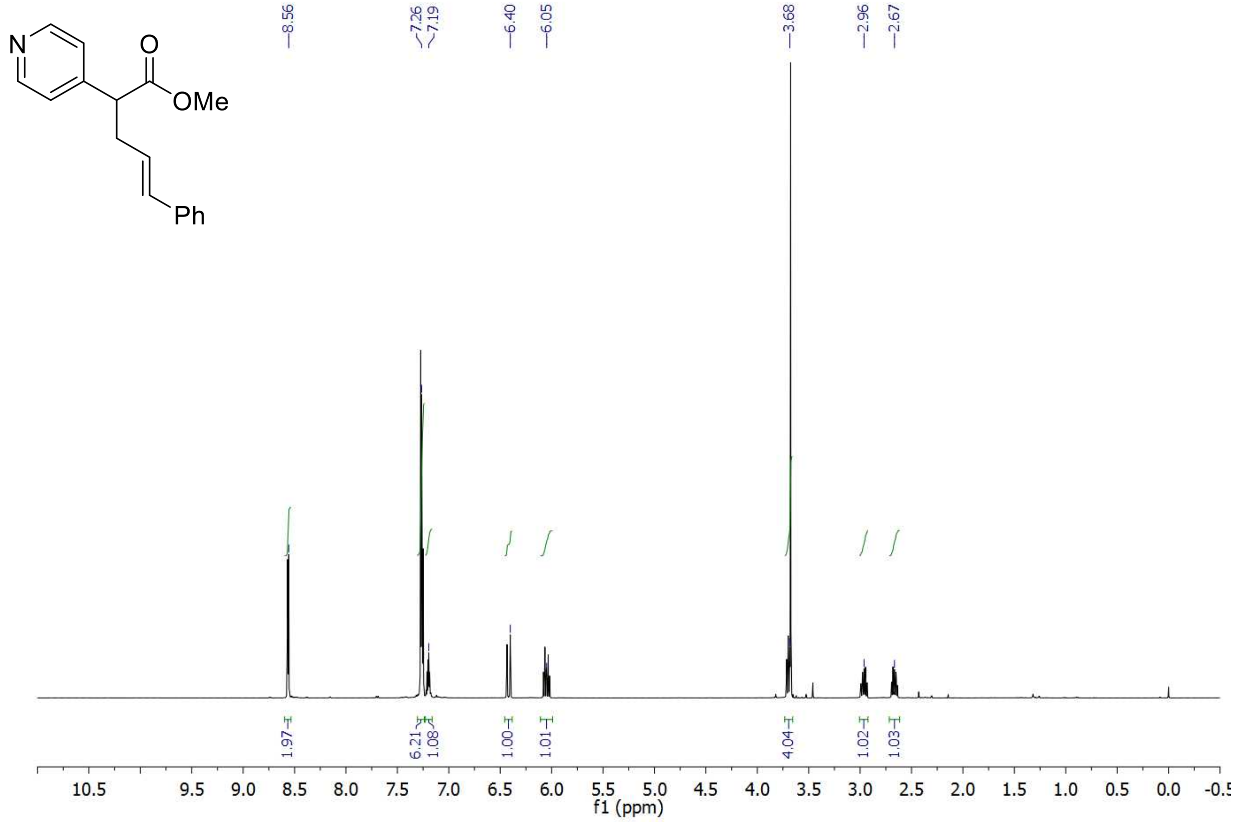

125 MHz $^{13}C\{^{1}H\}$ NMR of **3m** in $CDCl_3$

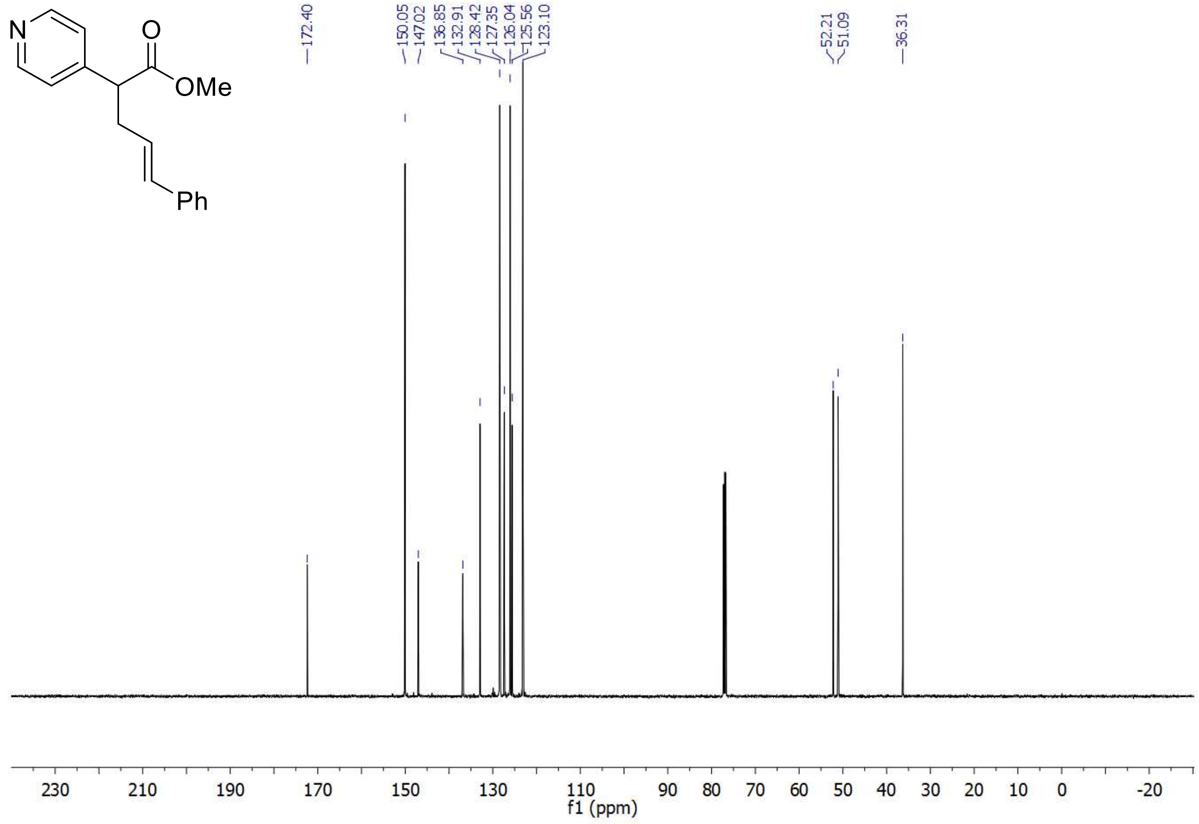

500 MHz ^{1}H NMR of **3n** in $CDCl_3$

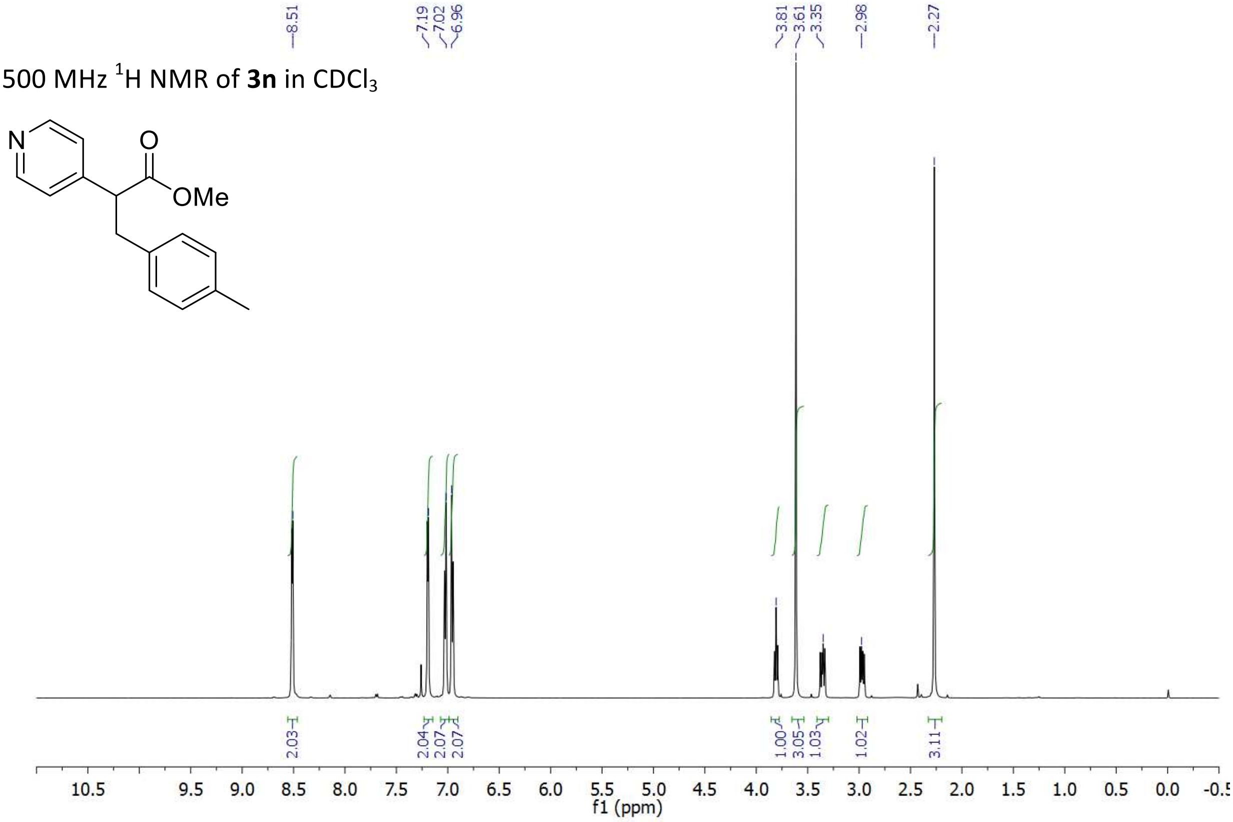

125 MHz $^{13}C\{^{1}H\}$ NMR of **3n** in $CDCl_3$

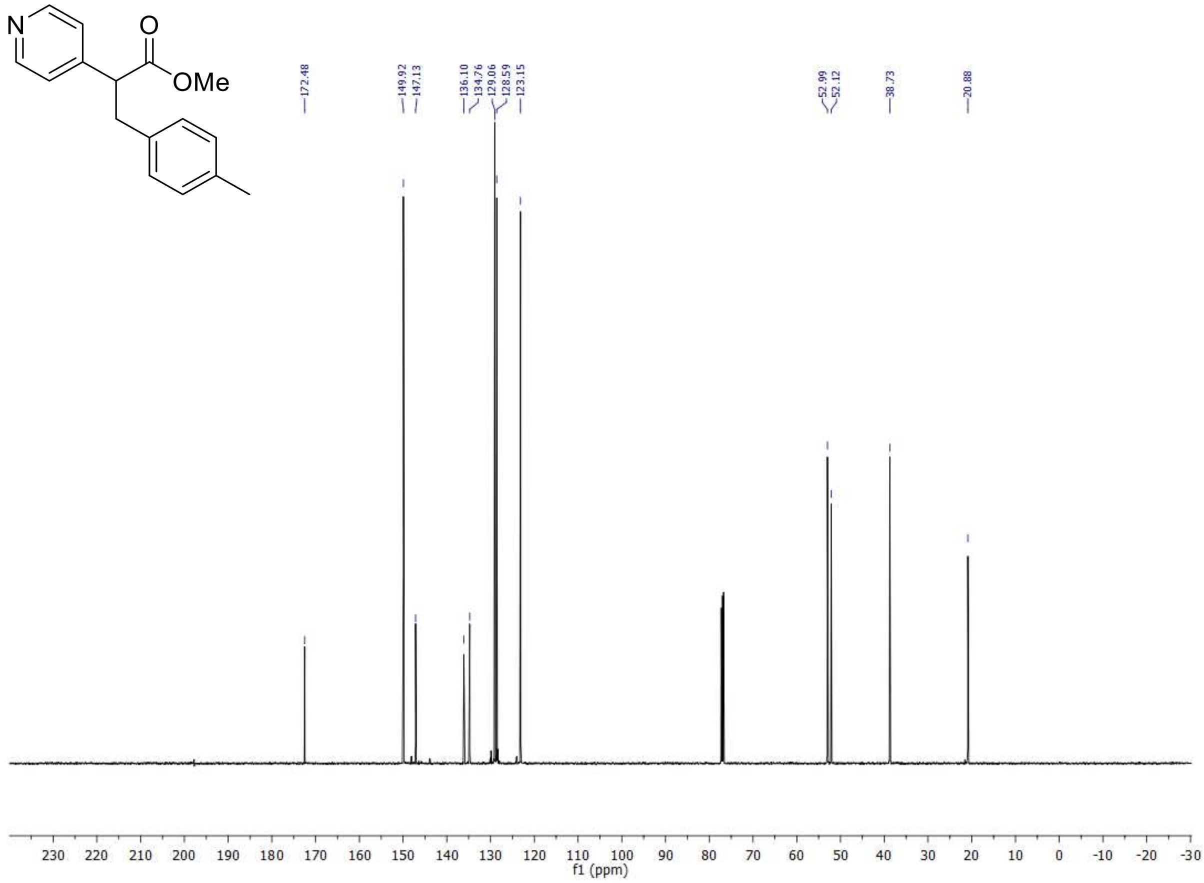

500 MHz ^{1}H NMR of **3o** in $CDCl_3$

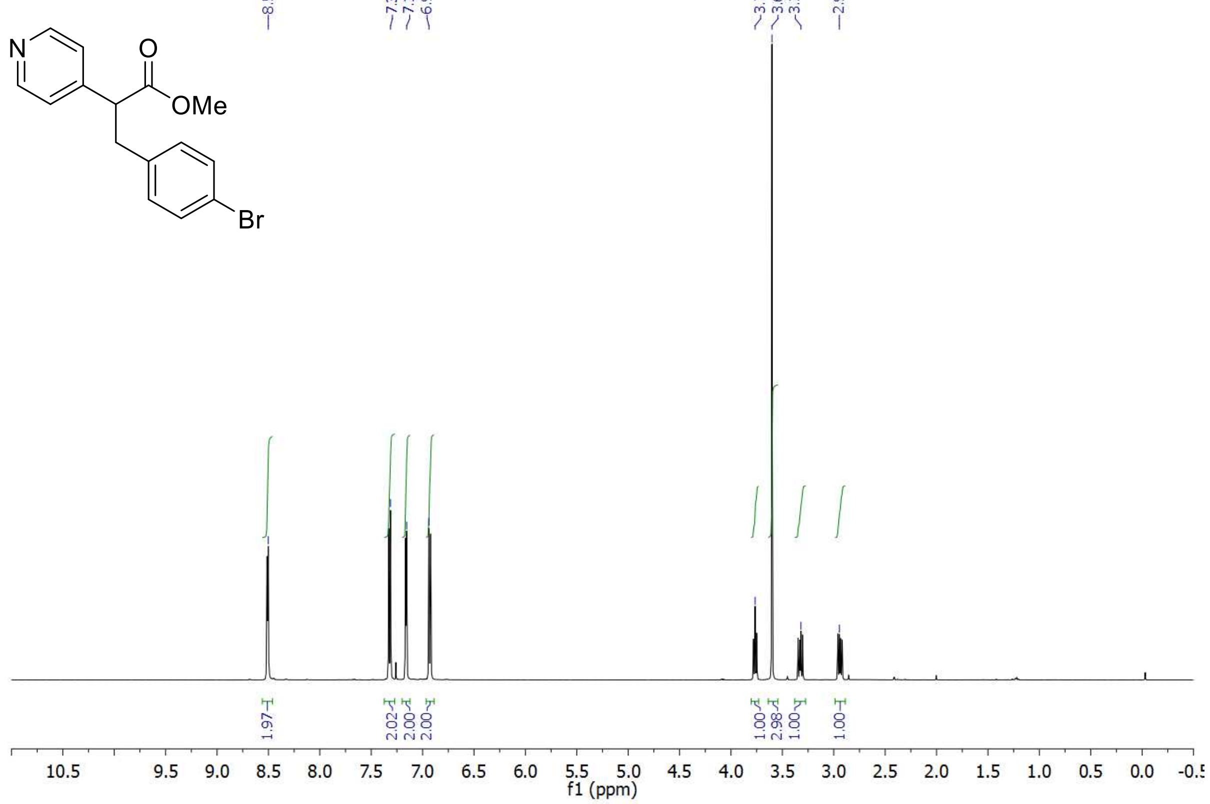

125 MHz $^{13}C\{^{1}H\}$ NMR of **3o** in $CDCl_3$

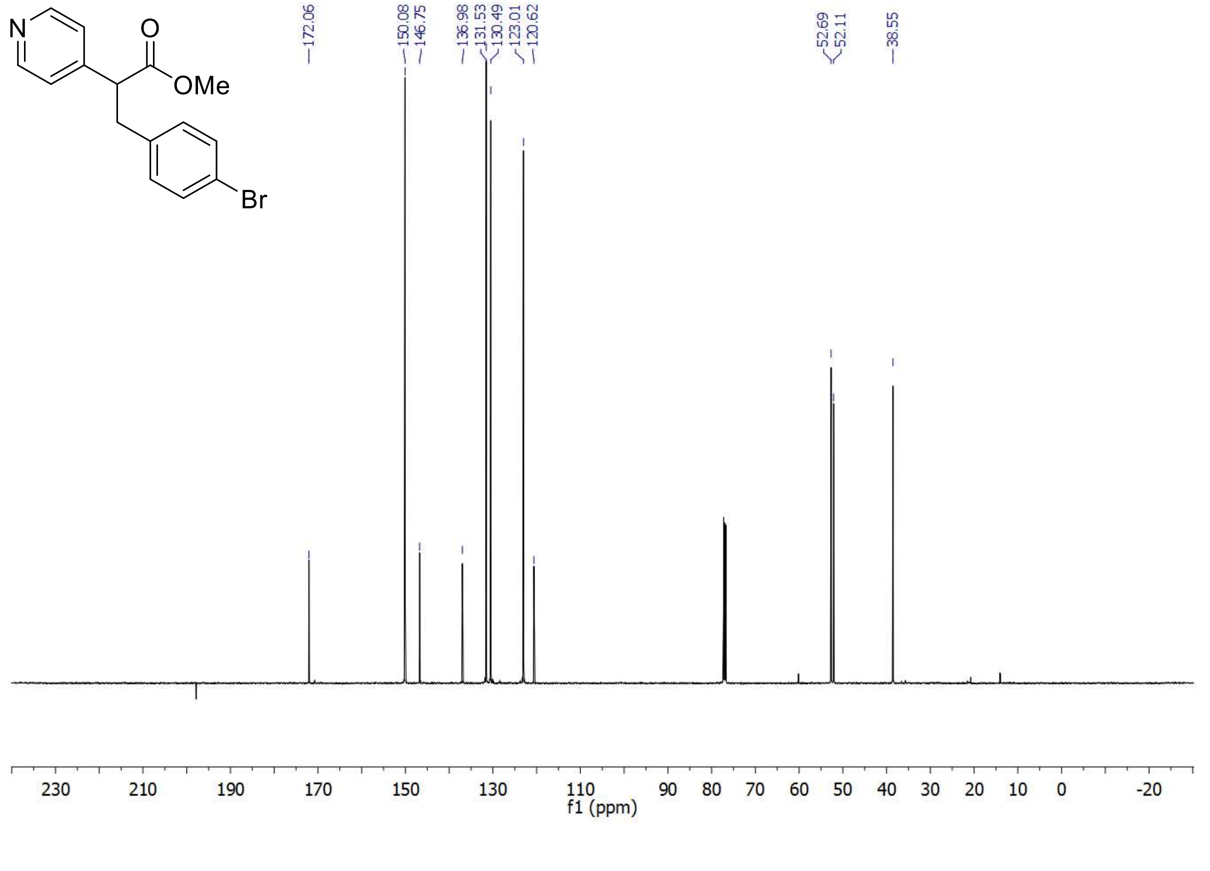

500 MHz ^{1}H NMR of **3p** in $CDCl_3$

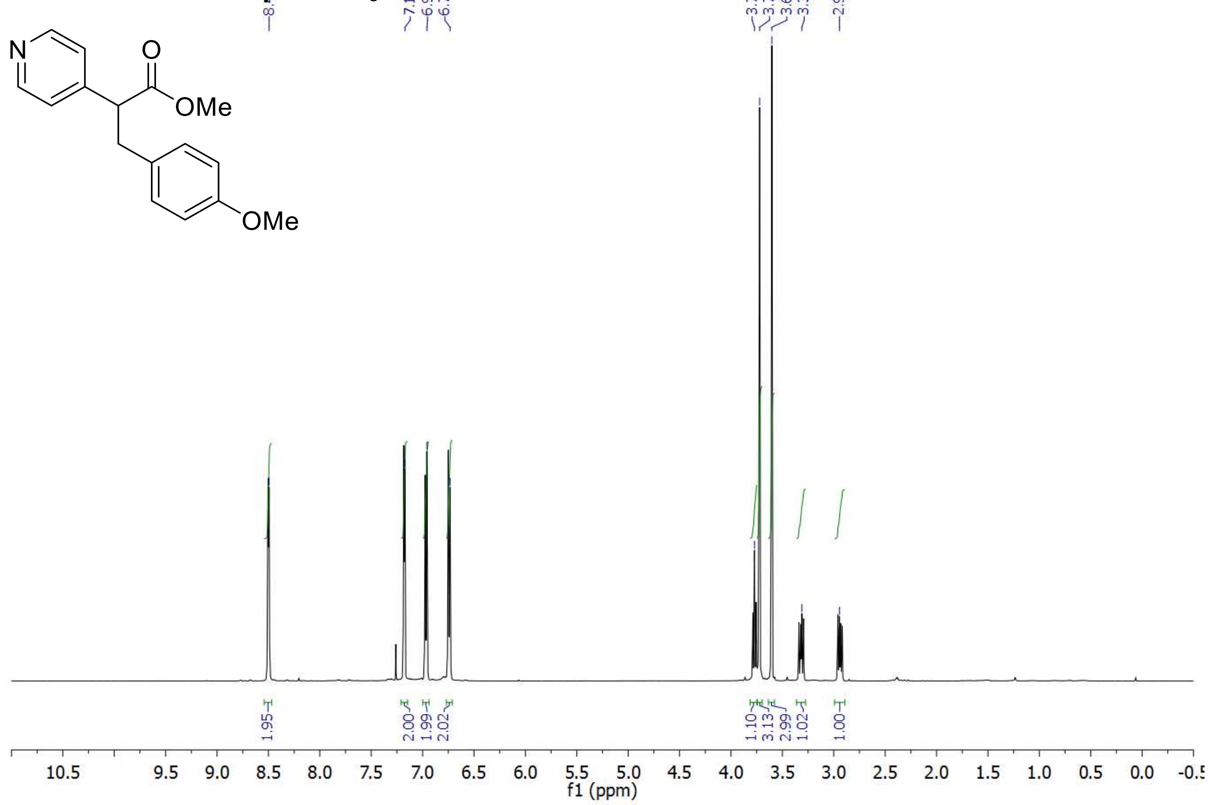

125 MHz $^{13}C\{^{1}H\}$ NMR of **3p** in $CDCl_3$

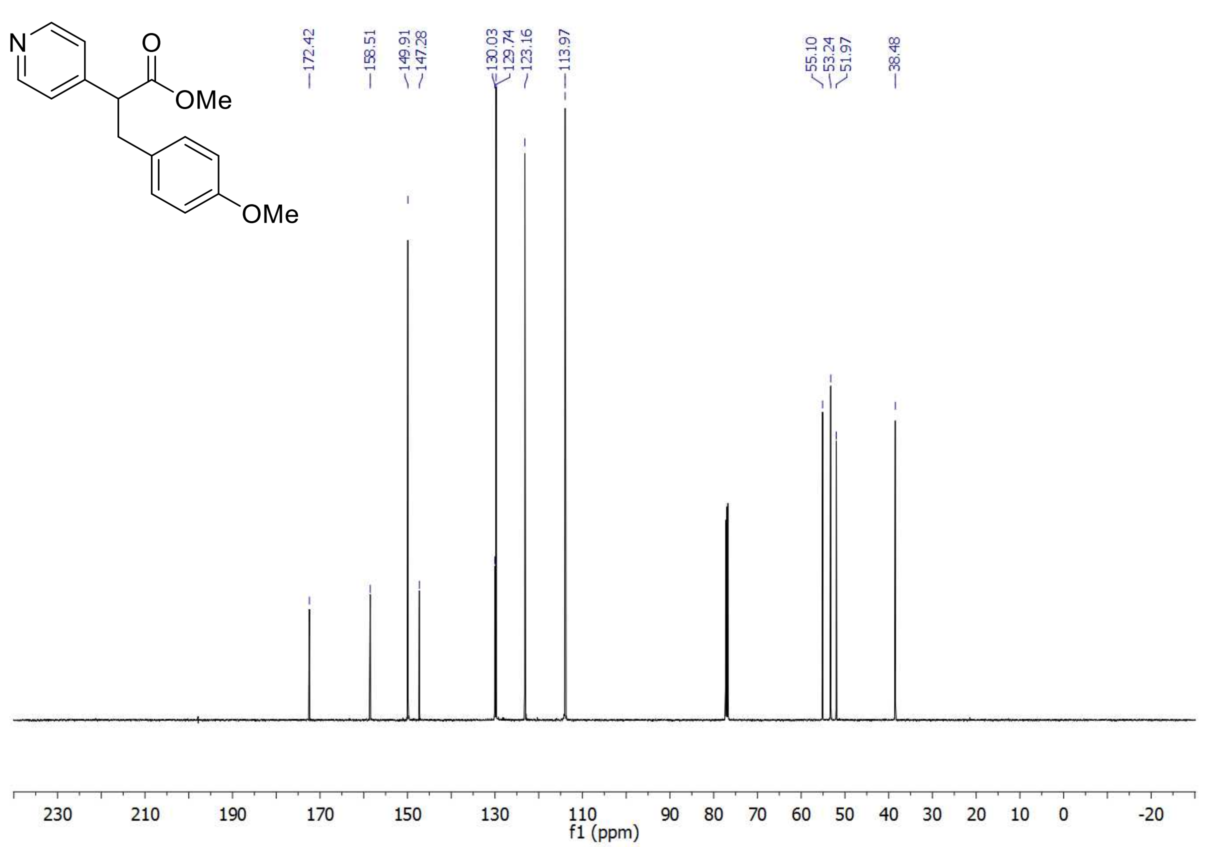

500 MHz ^{1}H NMR of **3q**in $CDCl_3$

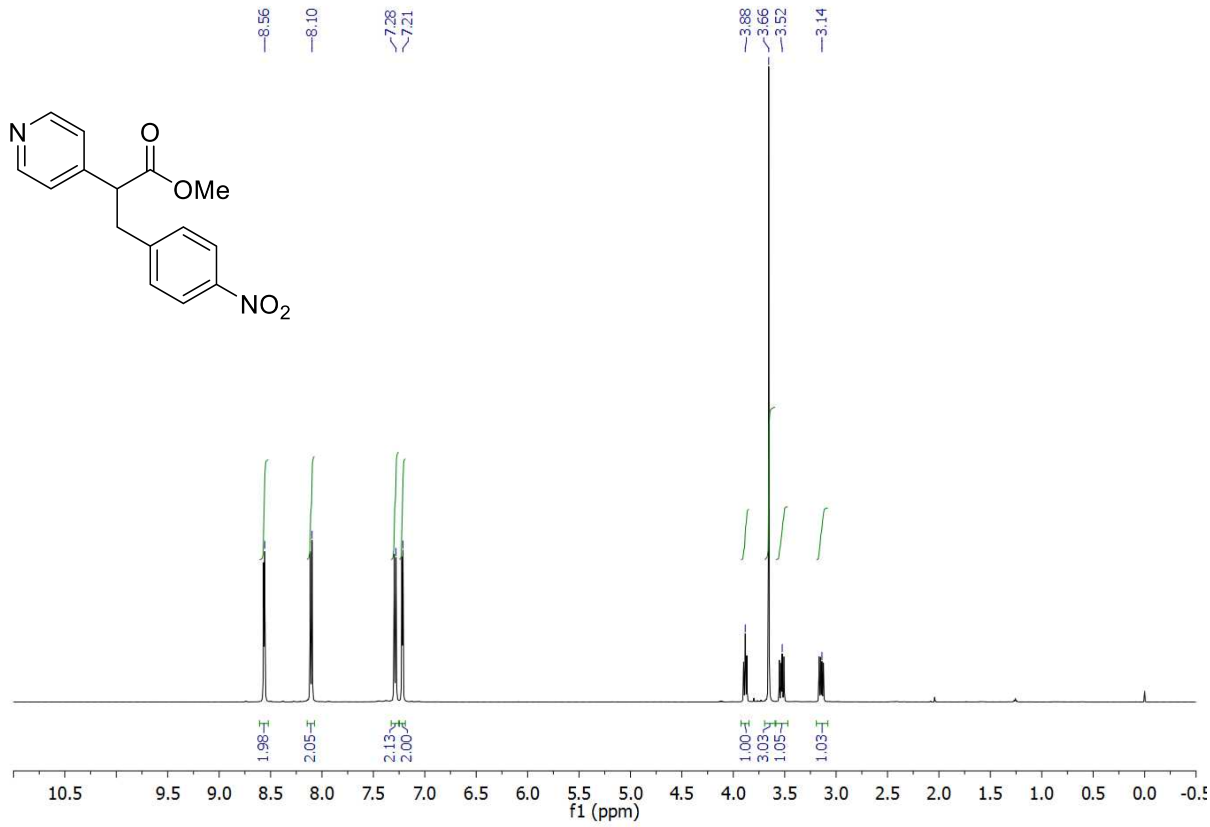

125 MHz $^{13}C\{^1H\}$ NMR of **3q** in $CDCl_3$

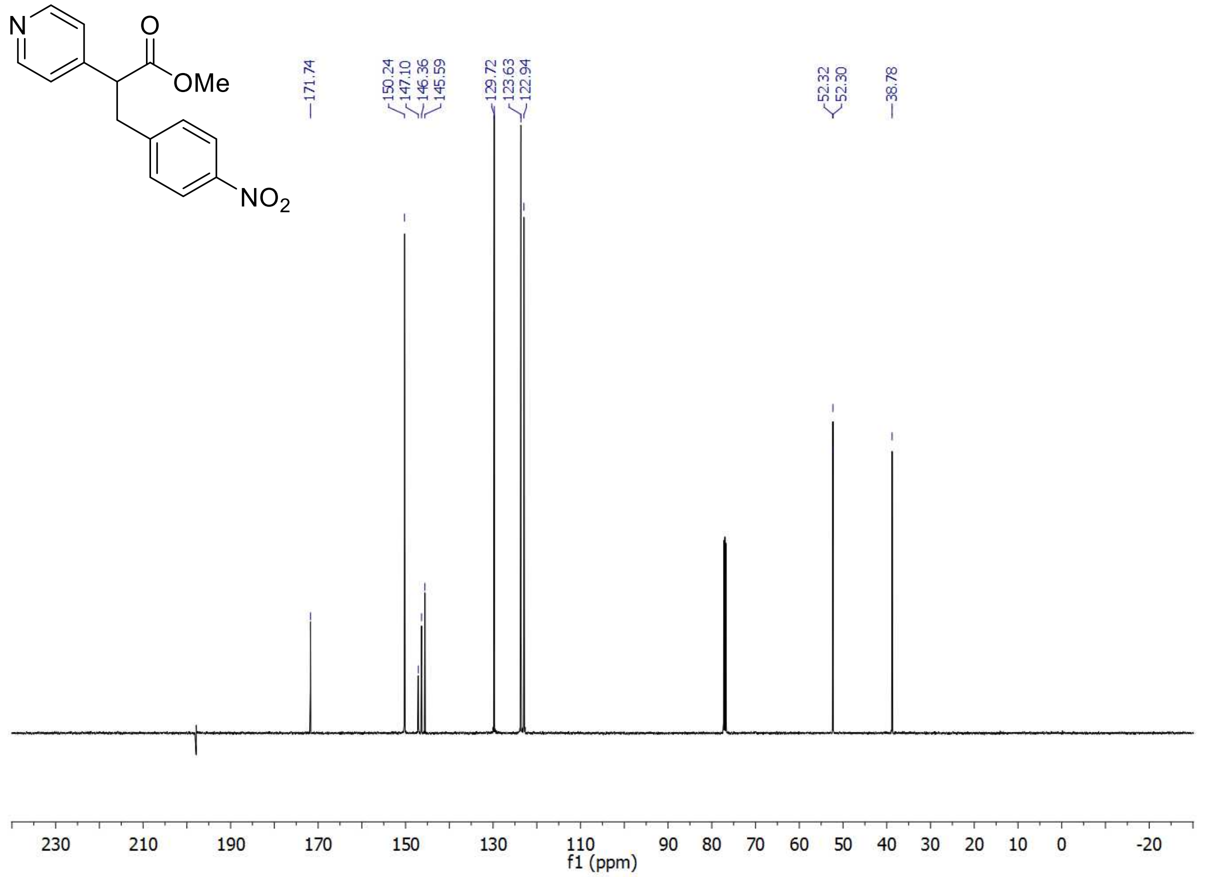

500 MHz ^{1}H NMR of **3r** in $CDCl_3$

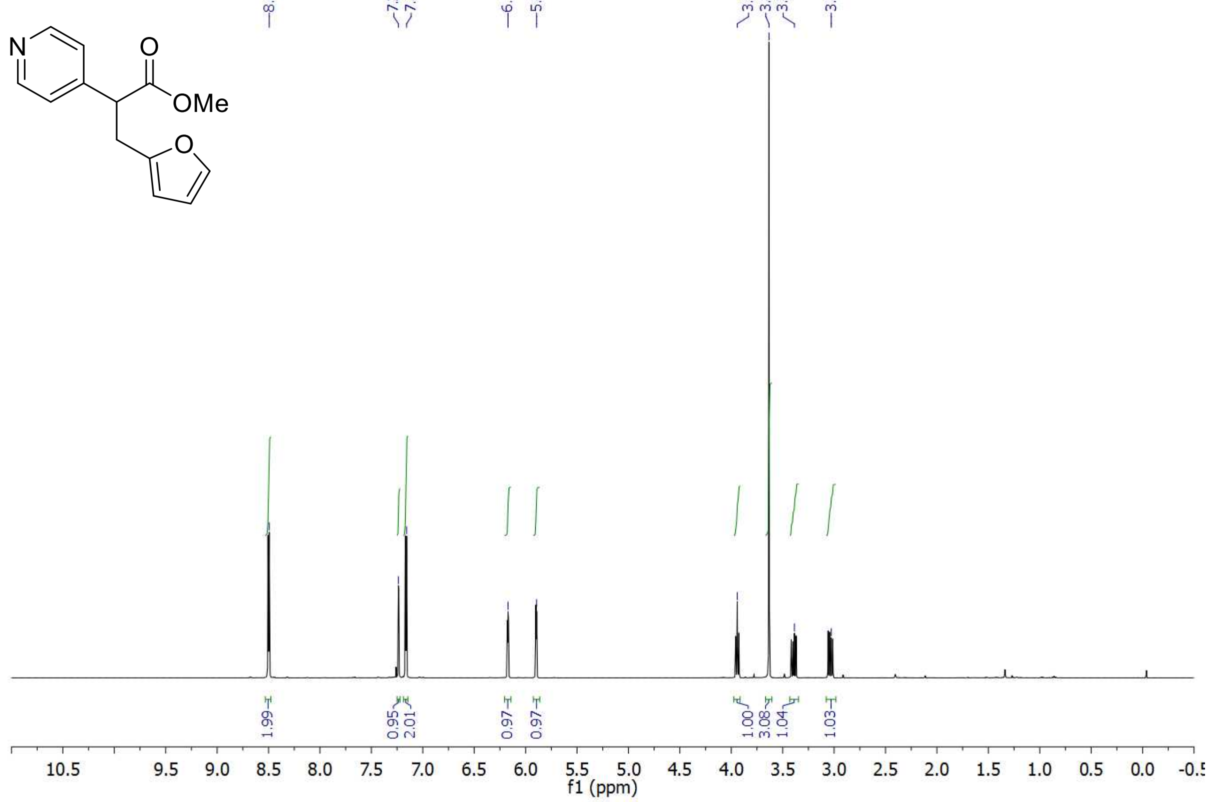

125 MHz $^{13}C\{^{1}H\}$ NMR of **3r** in $CDCl_3$

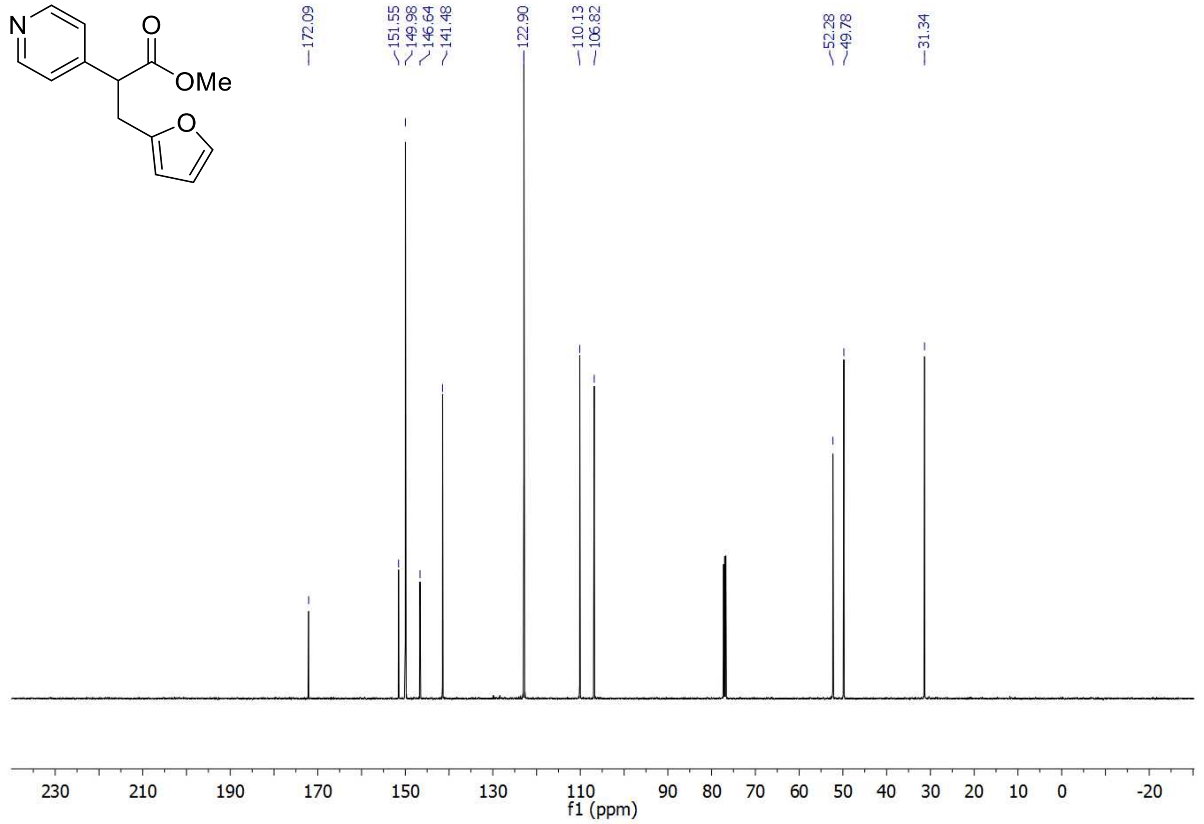

500 MHz ^{1}H NMR of **3s** in $CDCl_3$

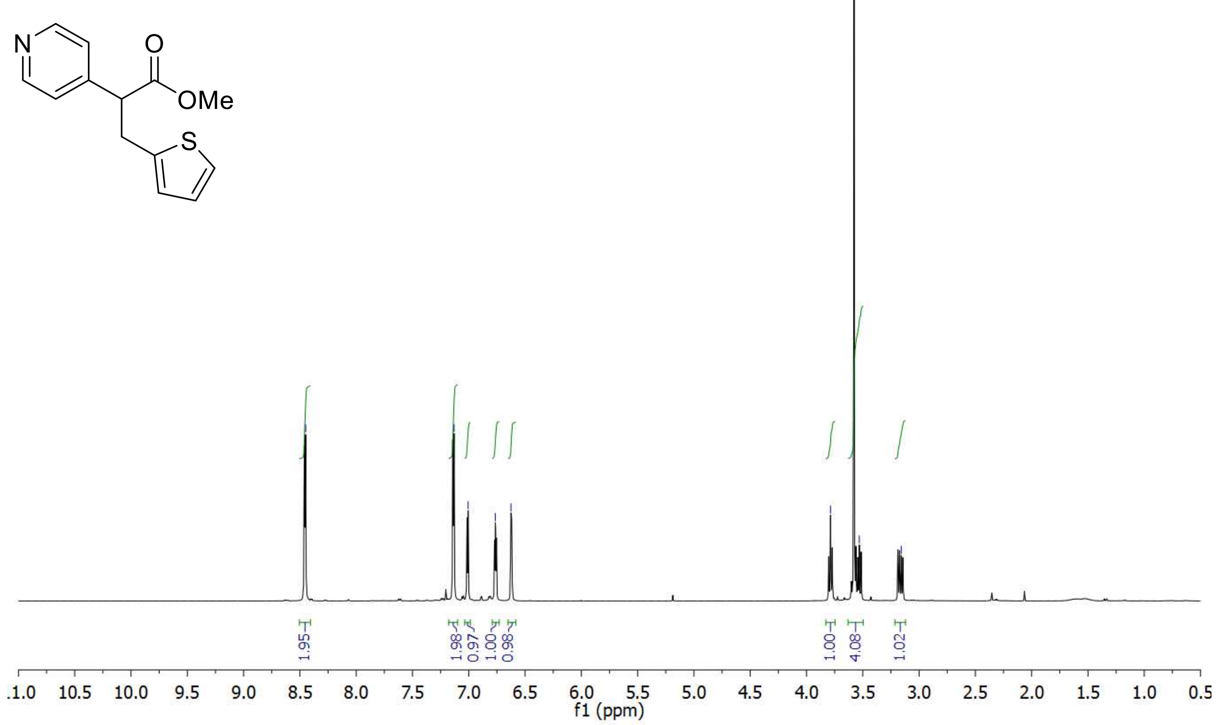

125 MHz $^{13}C\{^{1}H\}$ NMR of **3s** in $CDCl_3$

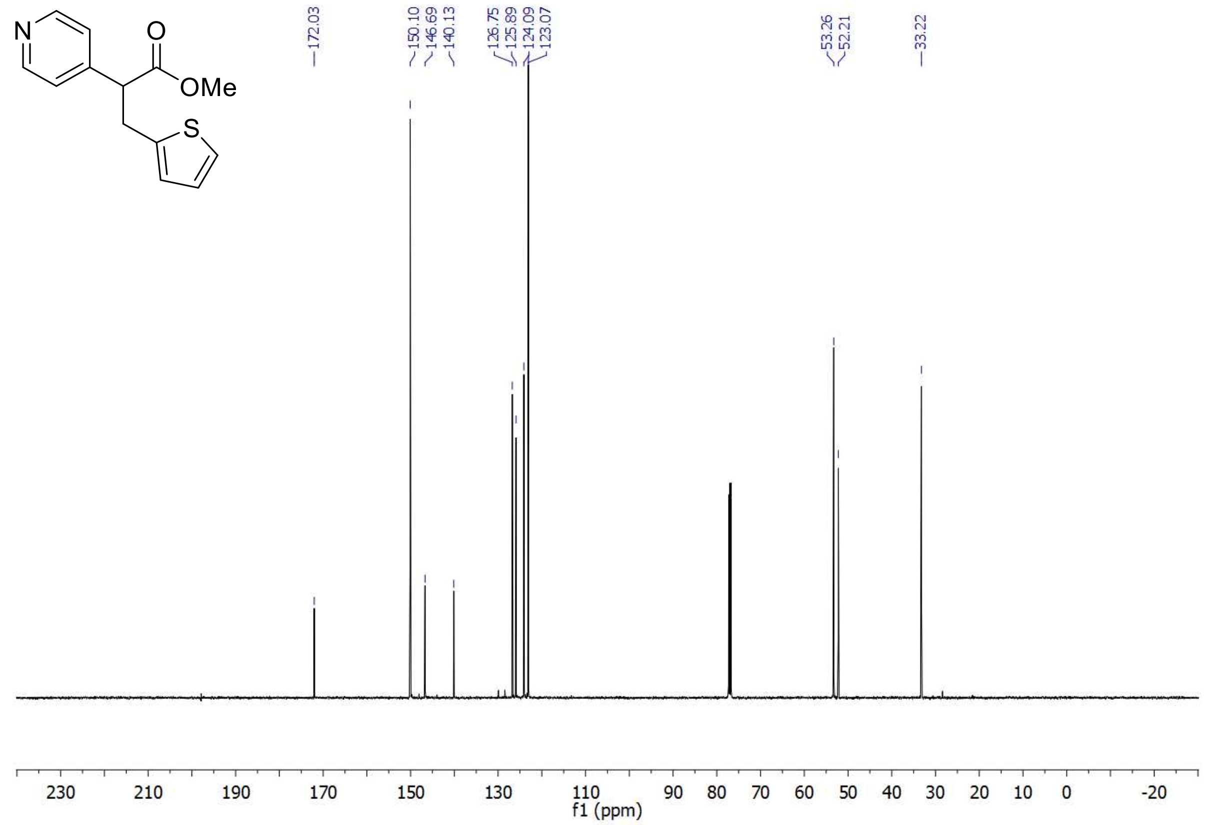

500 MHz ^{1}H NMR of **3t** in $CDCl_3$

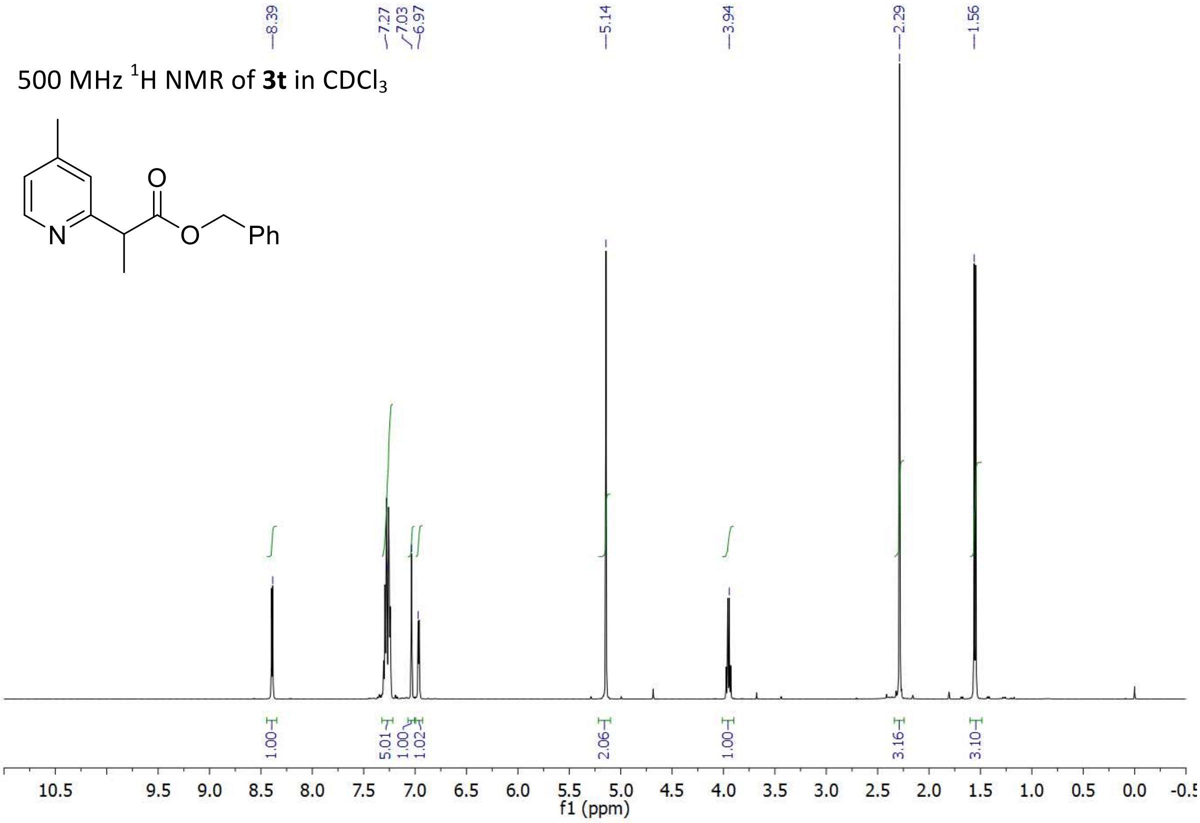

125 MHz $^{13}C\{^{1}H\}$ NMR of **3t** in $CDCl_3$

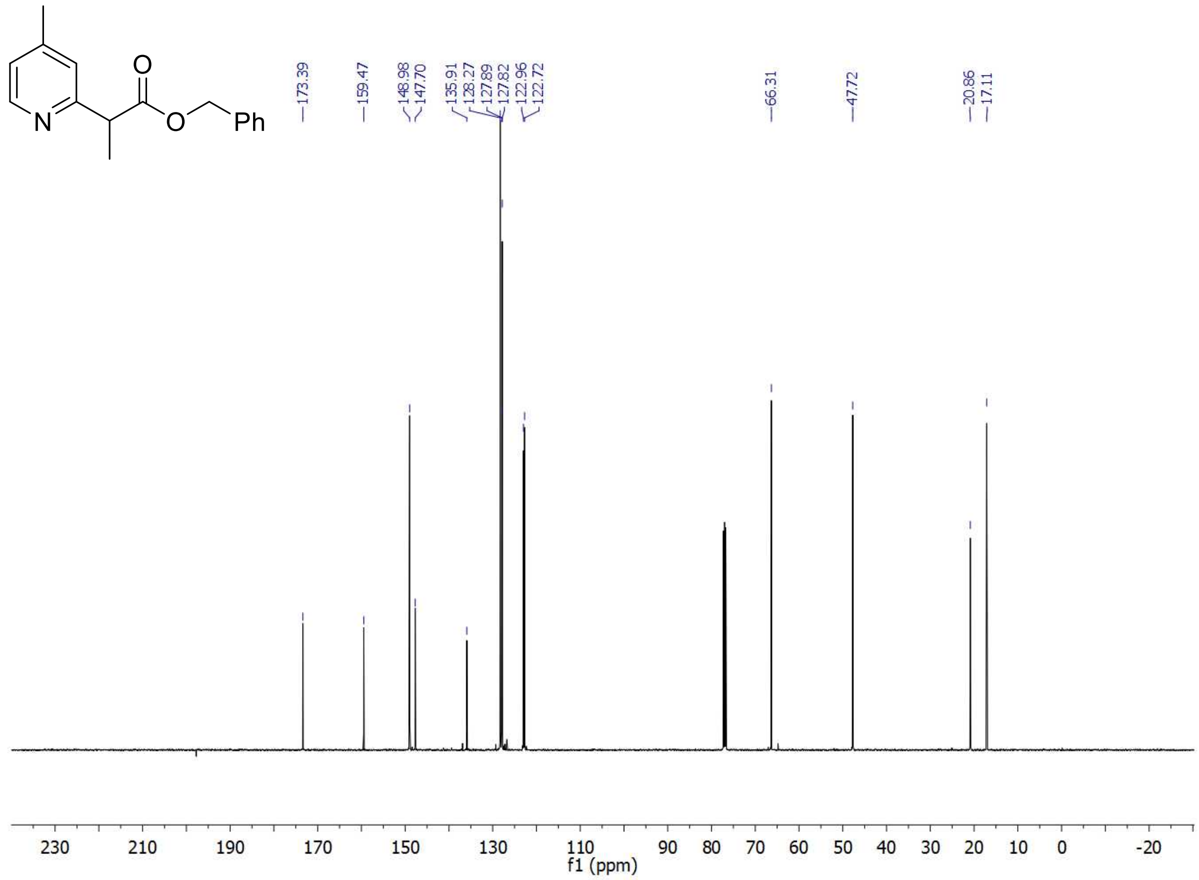

500 MHz ^{1}H NMR of **3u** in $CDCl_3$

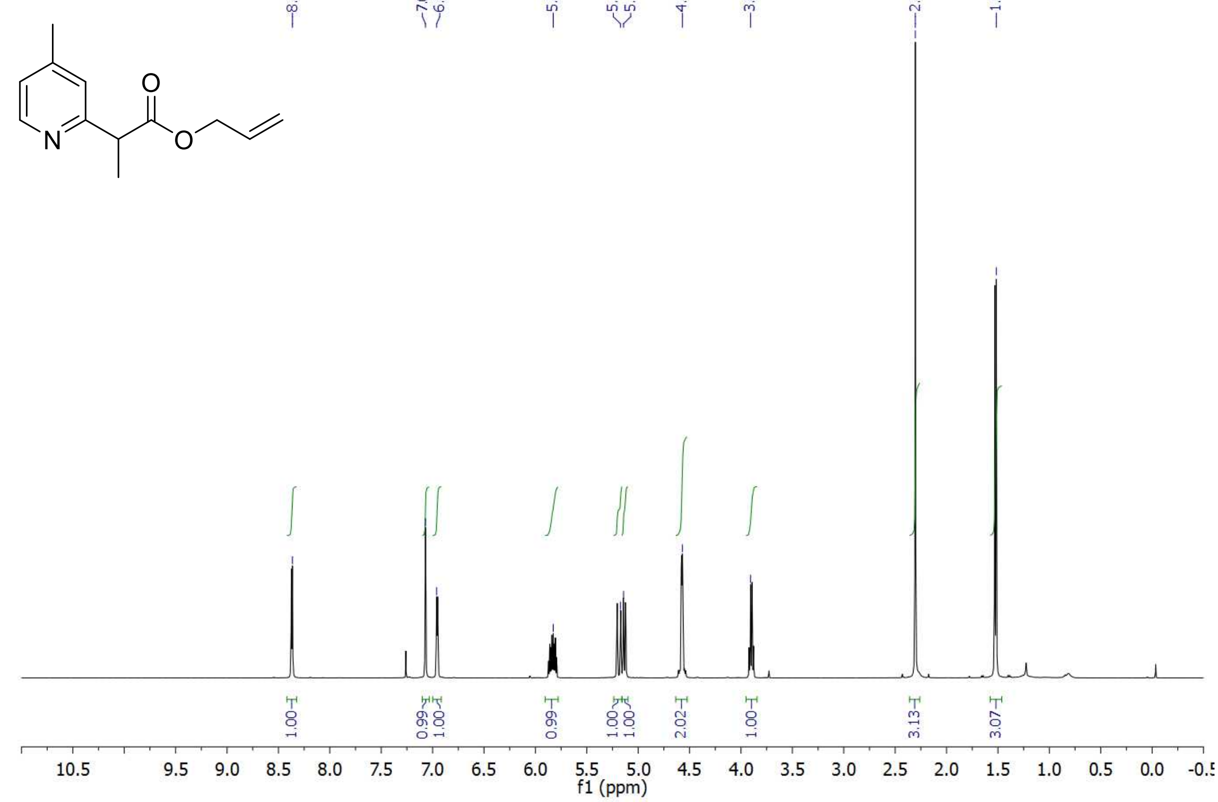

125 MHz $^{13}C\{^{1}H\}$ NMR of **3u** in $CDCl_3$

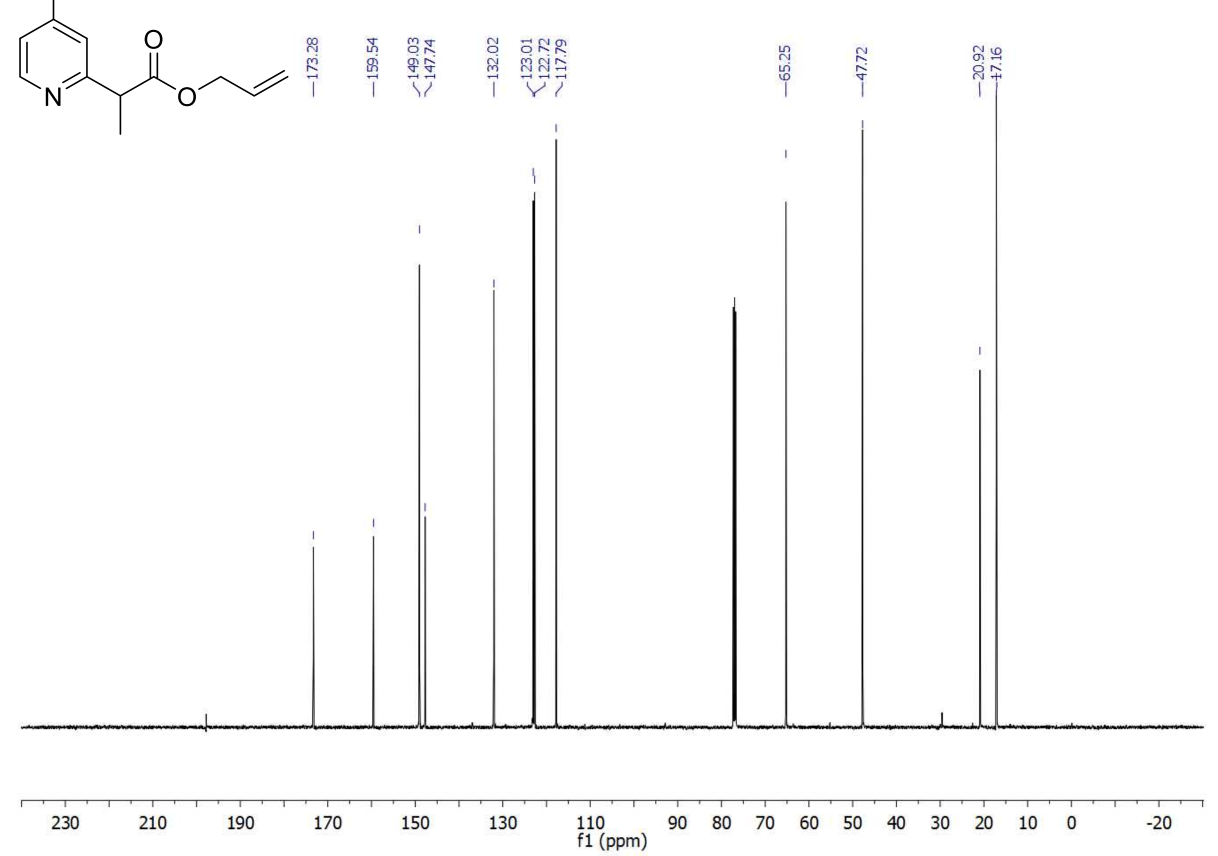

500 MHz ^{1}H NMR of **3v** in $CDCl_3$

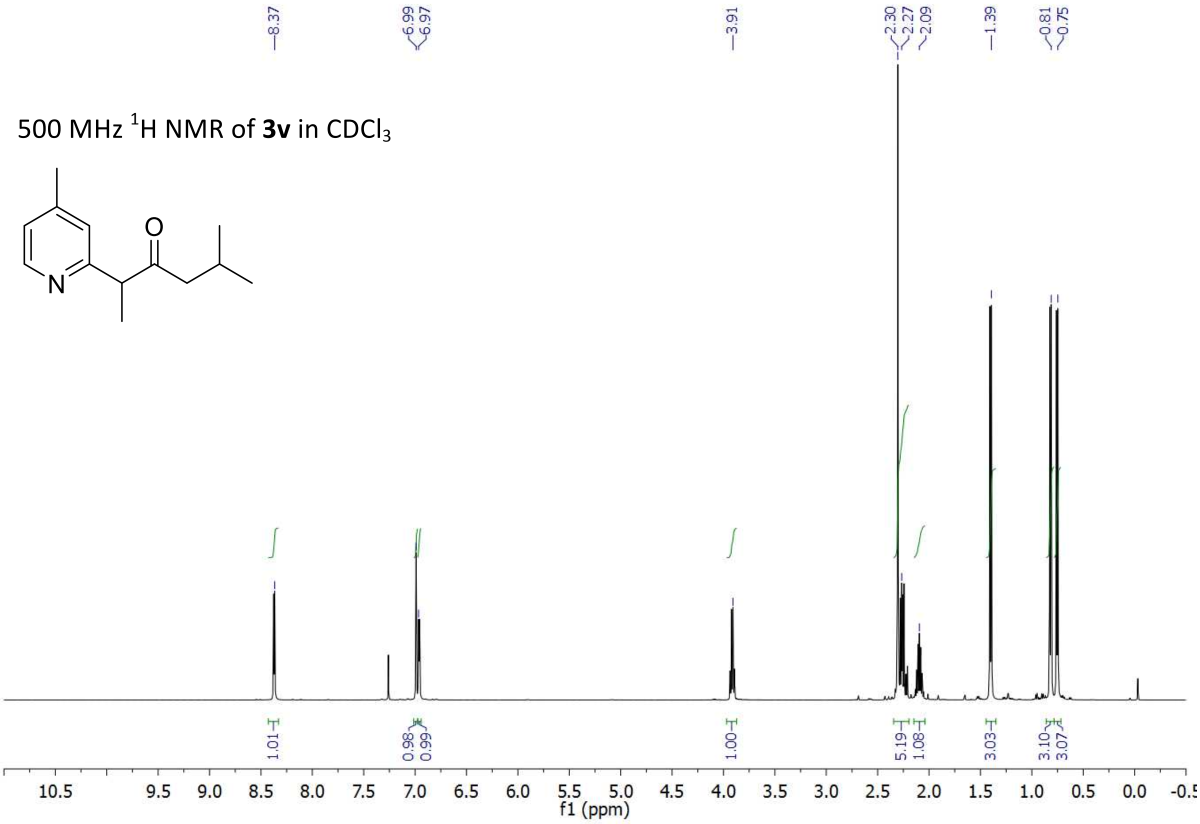

125 MHz ^{13}C{^{1}H} NMR of **3v** in $CDCl_3$

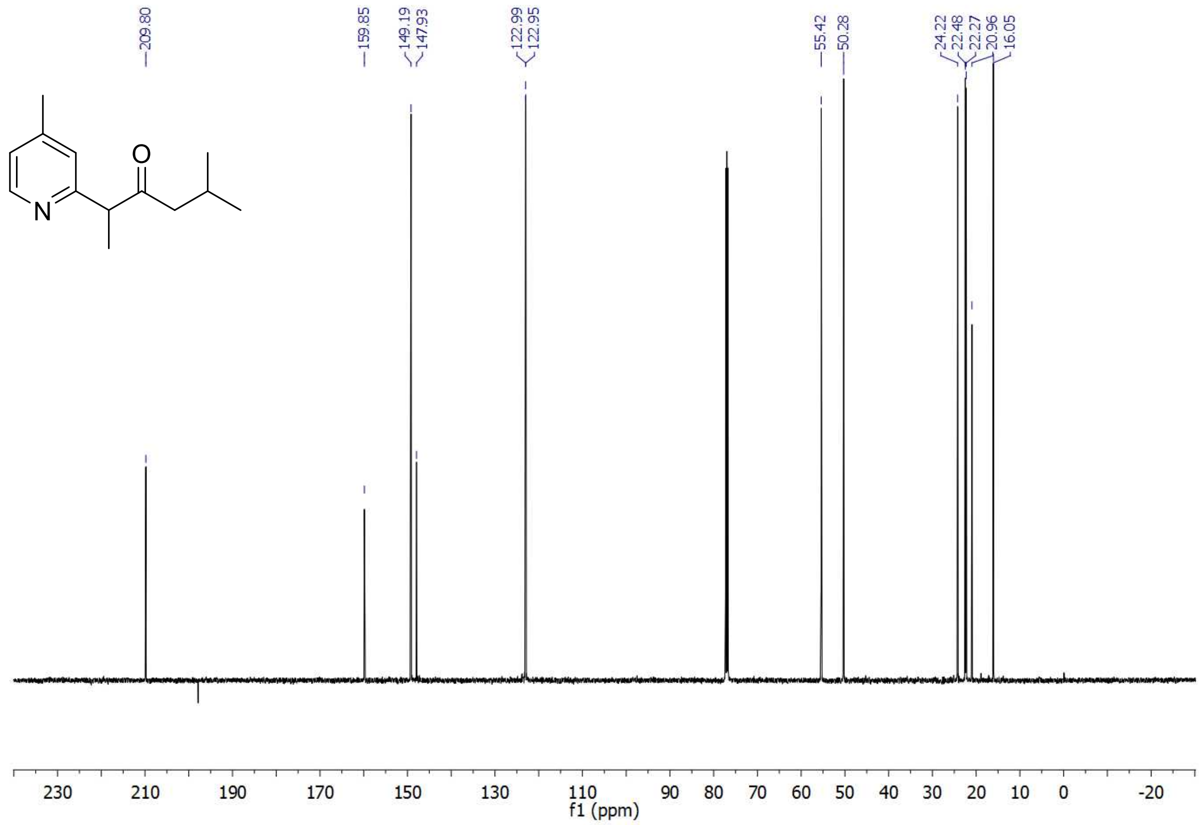

500 MHz ^{1}H NMR of **3w** in $CDCl_3$

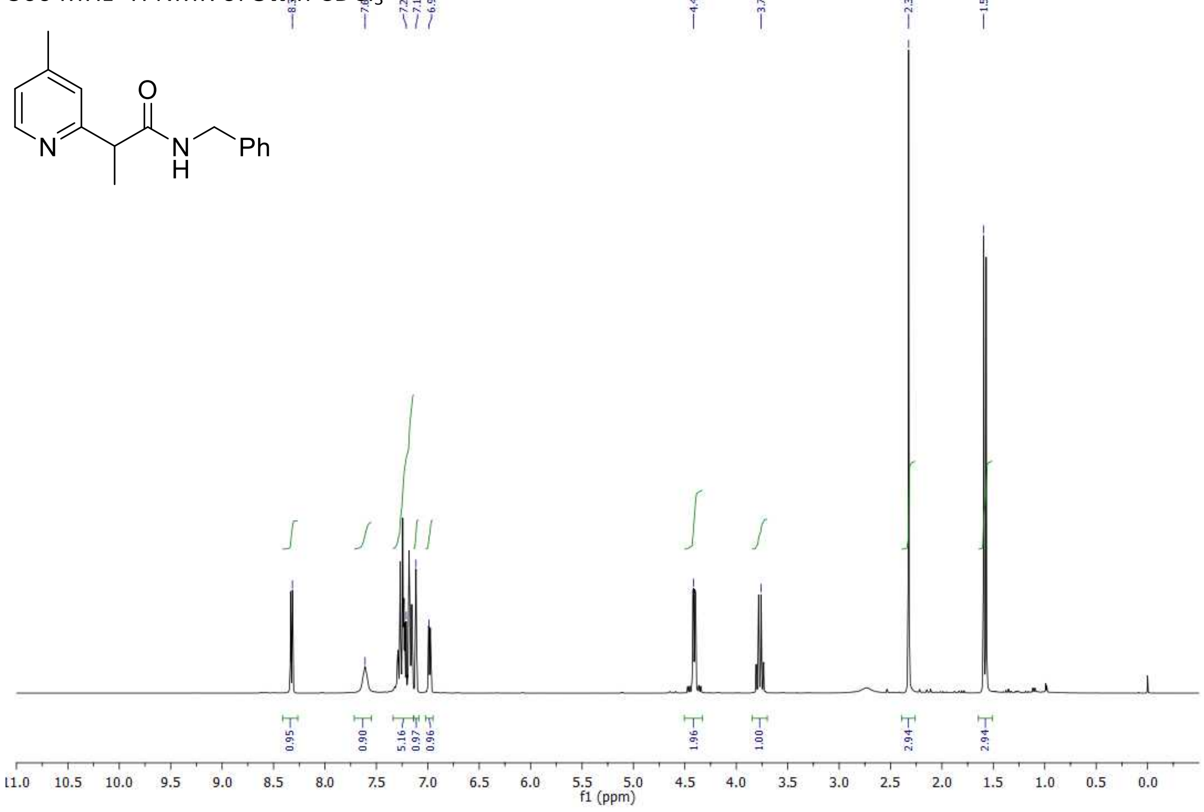

125 MHz $^{13}C\{^{1}H\}$ NMR of **3w** in $CDCl_3$

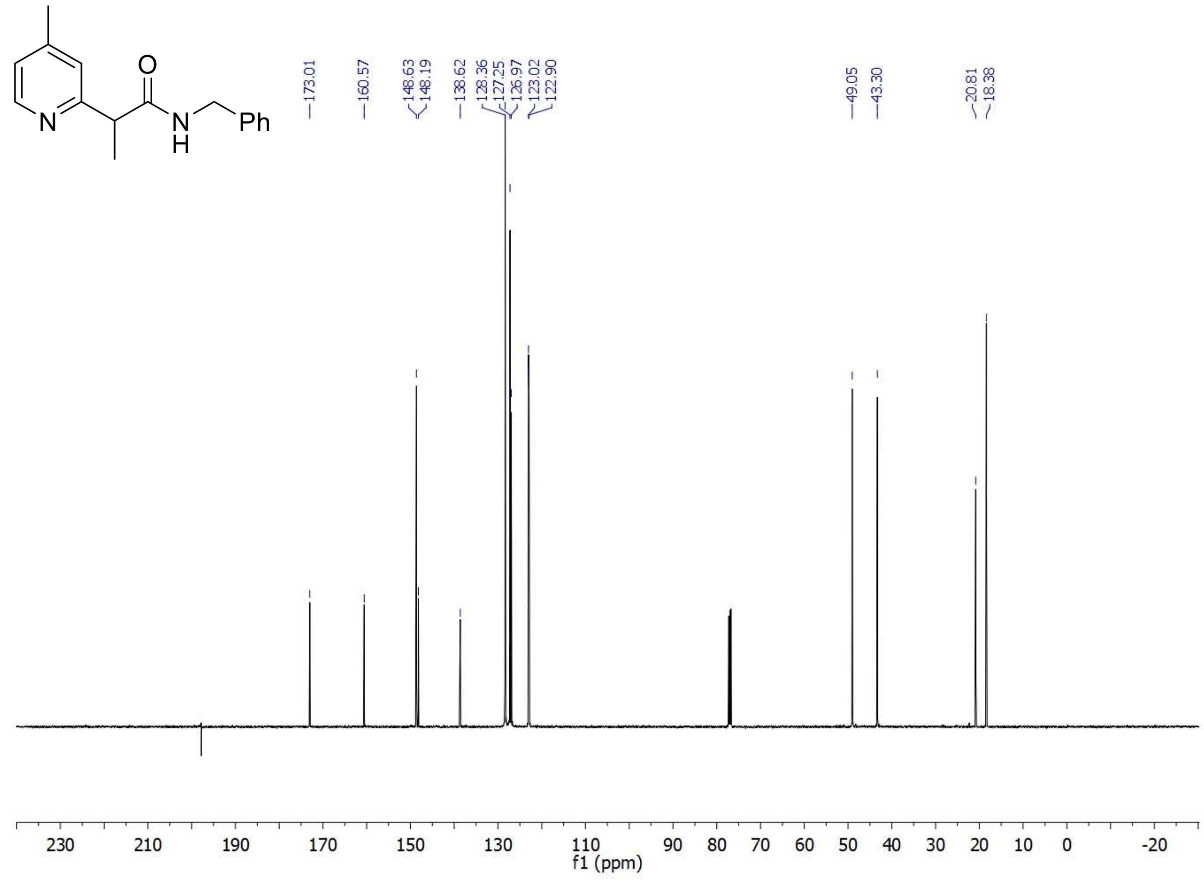

500 MHz ^{1}H NMR of **3x** in $CDCl_3$

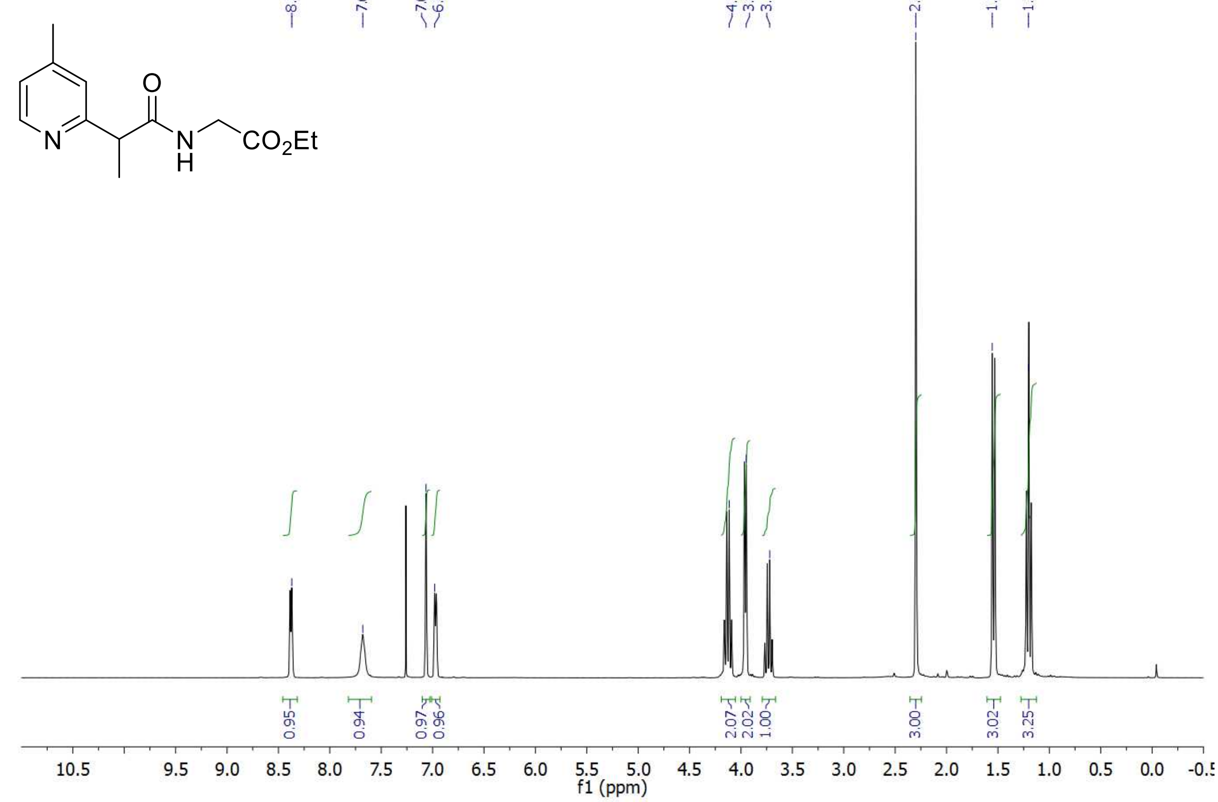

125 MHz $^{13}C\{^{1}H\}$ NMR of **3x** in $CDCl_3$

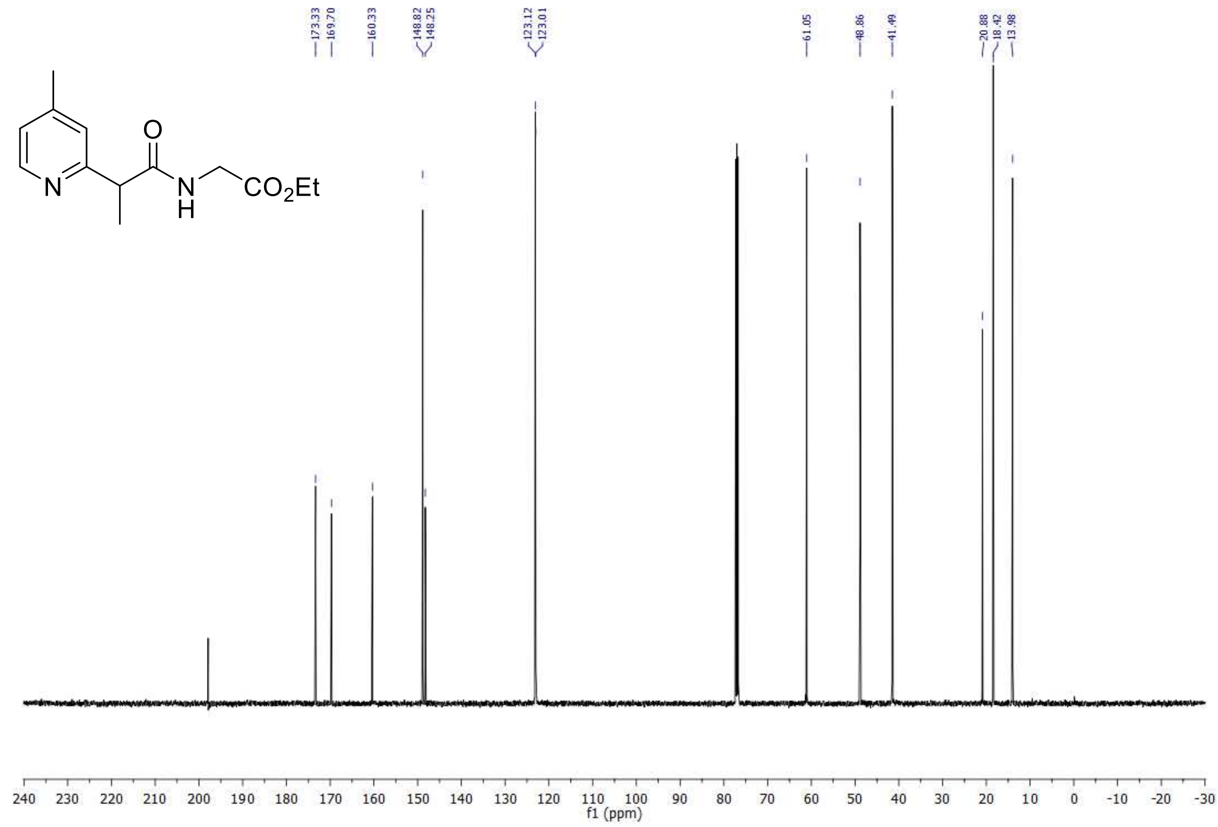

500 MHz ^{1}H NMR of **3y** in $CDCl_3$

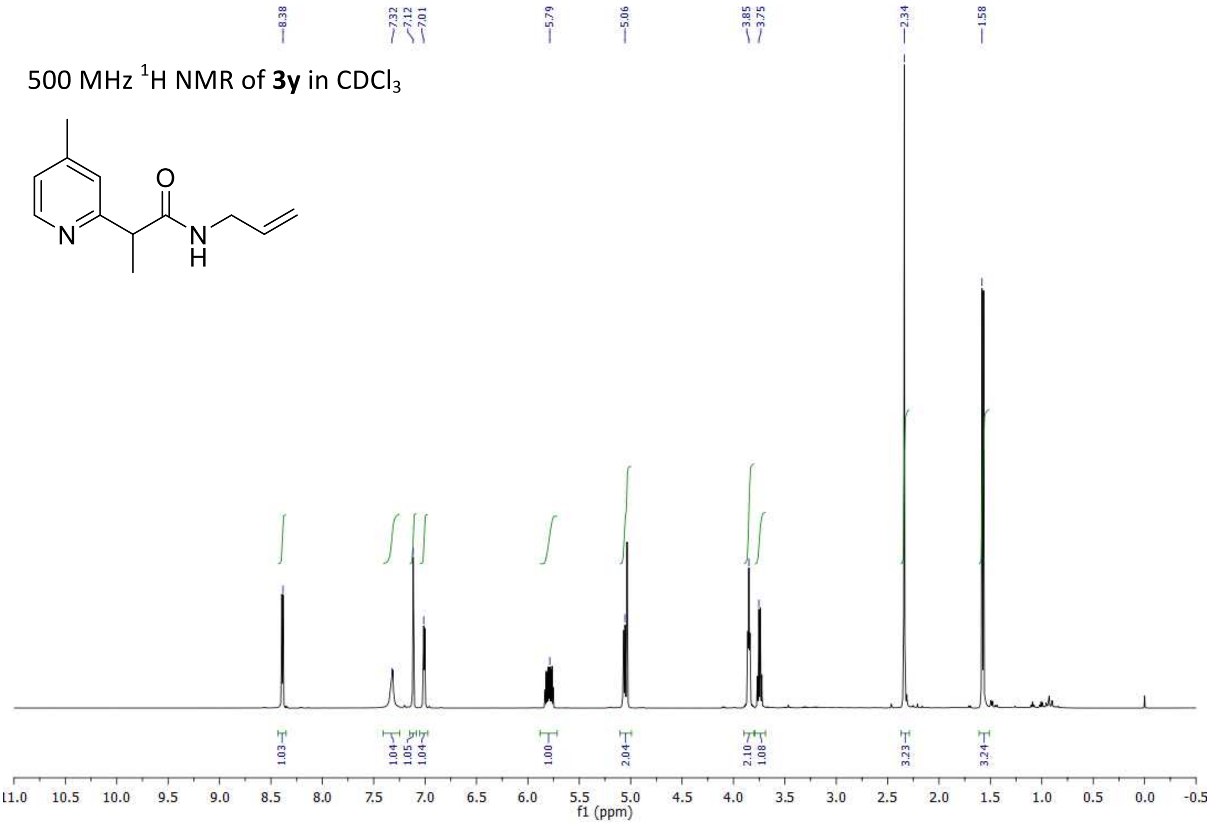

125 MHz $^{13}C\{^{1}H\}$ NMR of **3y** in $CDCl_3$

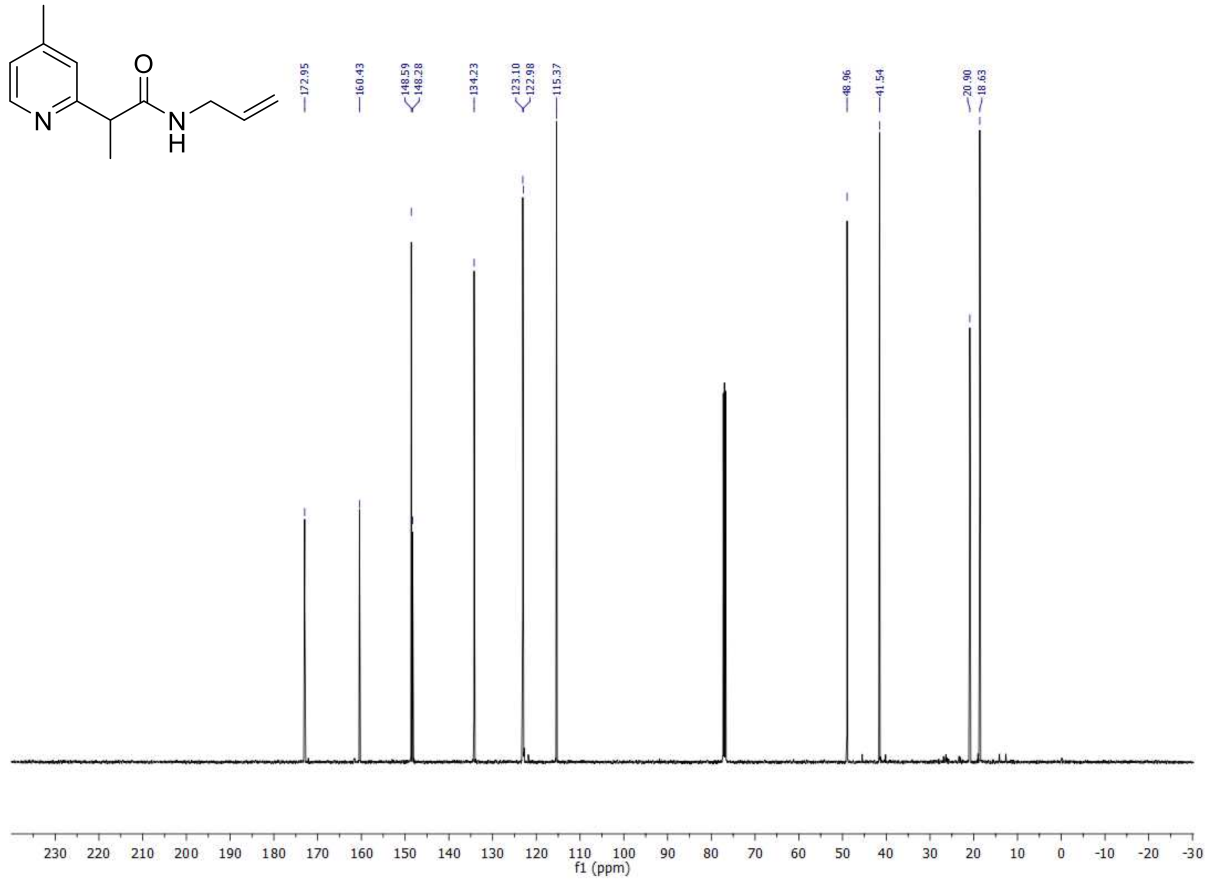

500 MHz ^{1}H NMR of **3z** in $CDCl_3$

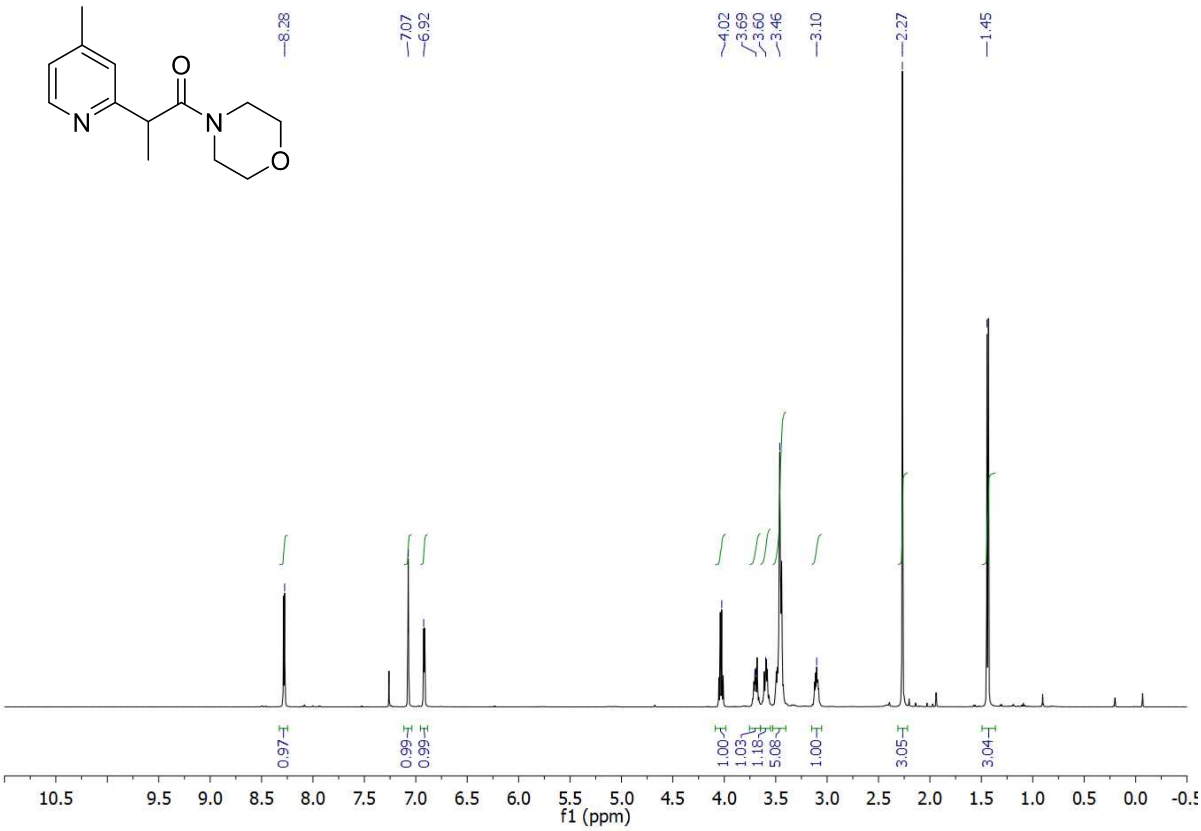

125 MHz $^{13}C\{^{1}H\}$ NMR of **3z** in $CDCl_3$

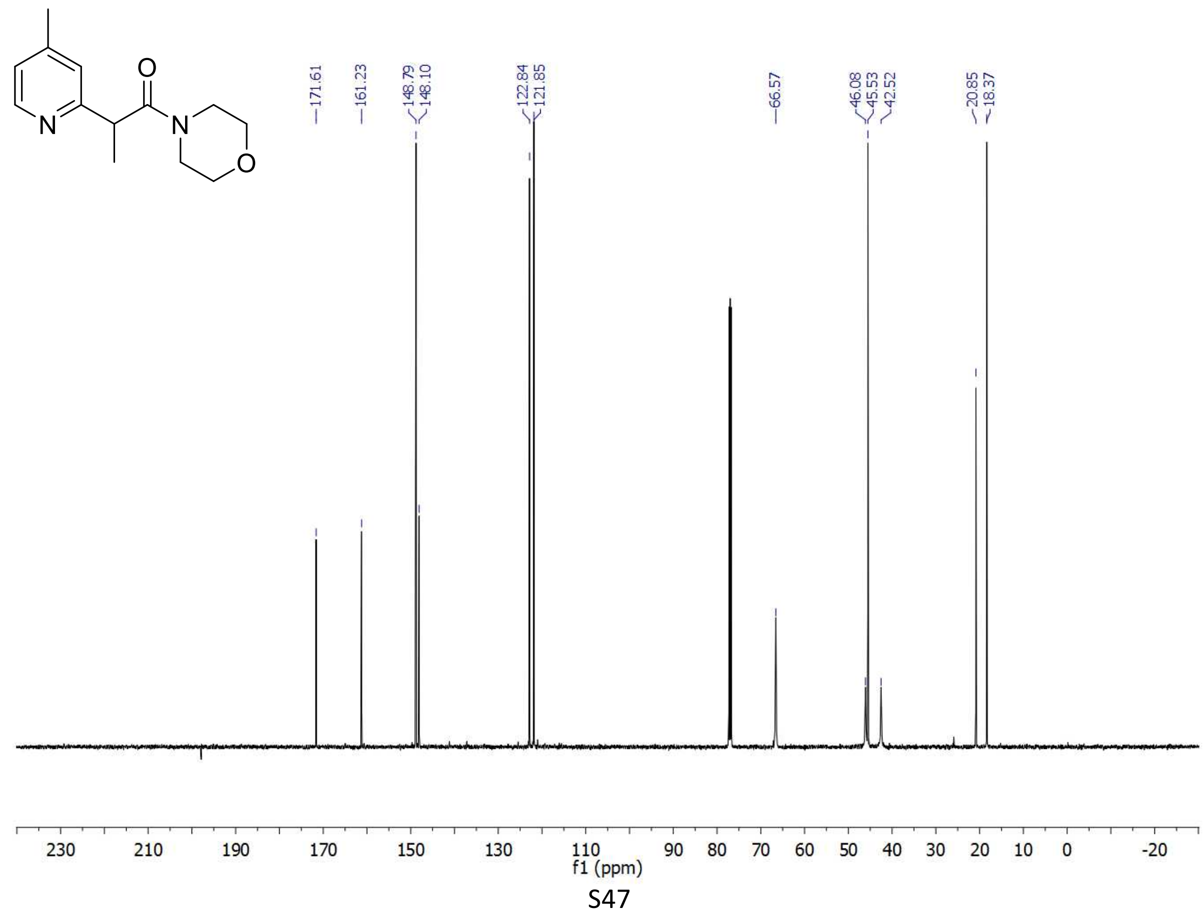

500 MHz ^{1}H NMR of **3aa** in $CDCl_3$

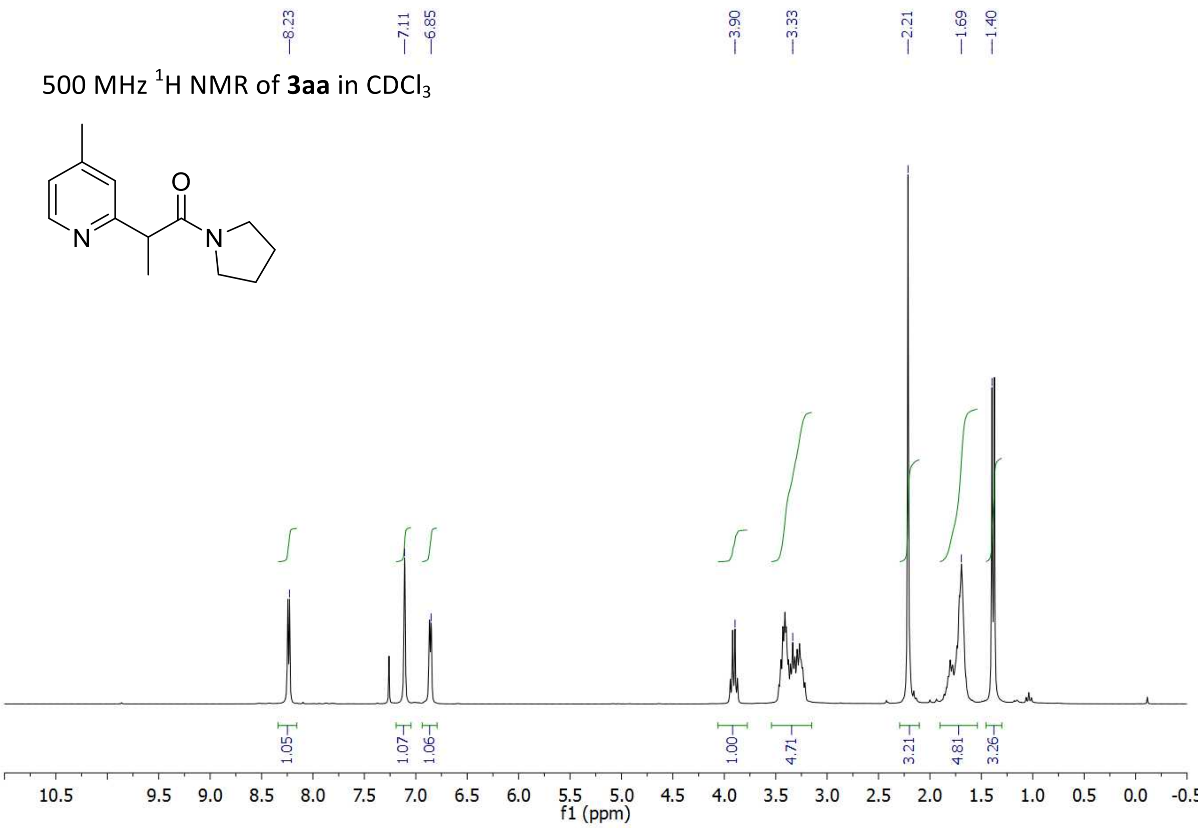

125 MHz $^{13}C\{^{1}H\}$ NMR of **3aa** in $CDCl_3$

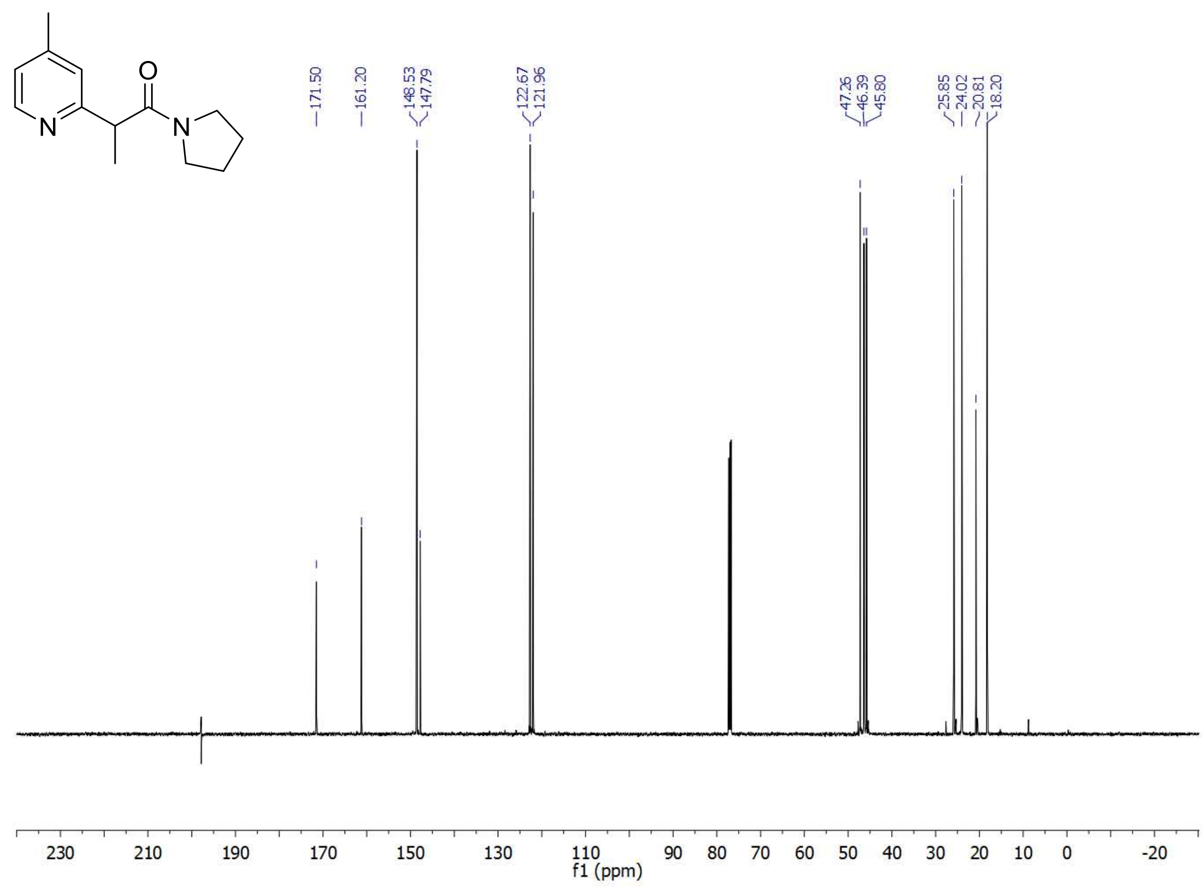

500 MHz ^{1}H NMR of **3ab** in $CDCl_3$

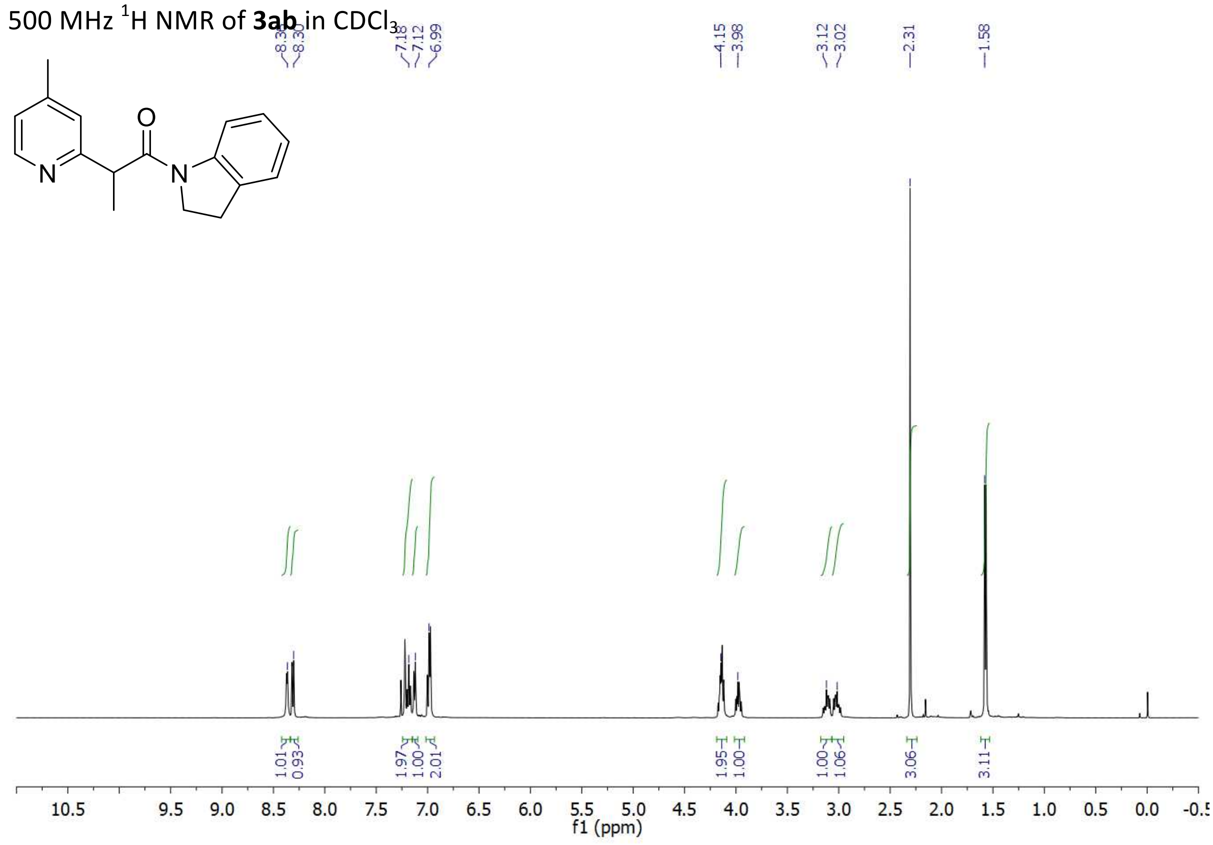

125 MHz $^{13}C\{^{1}H\}$ NMR of **3ab** in $CDCl_3$

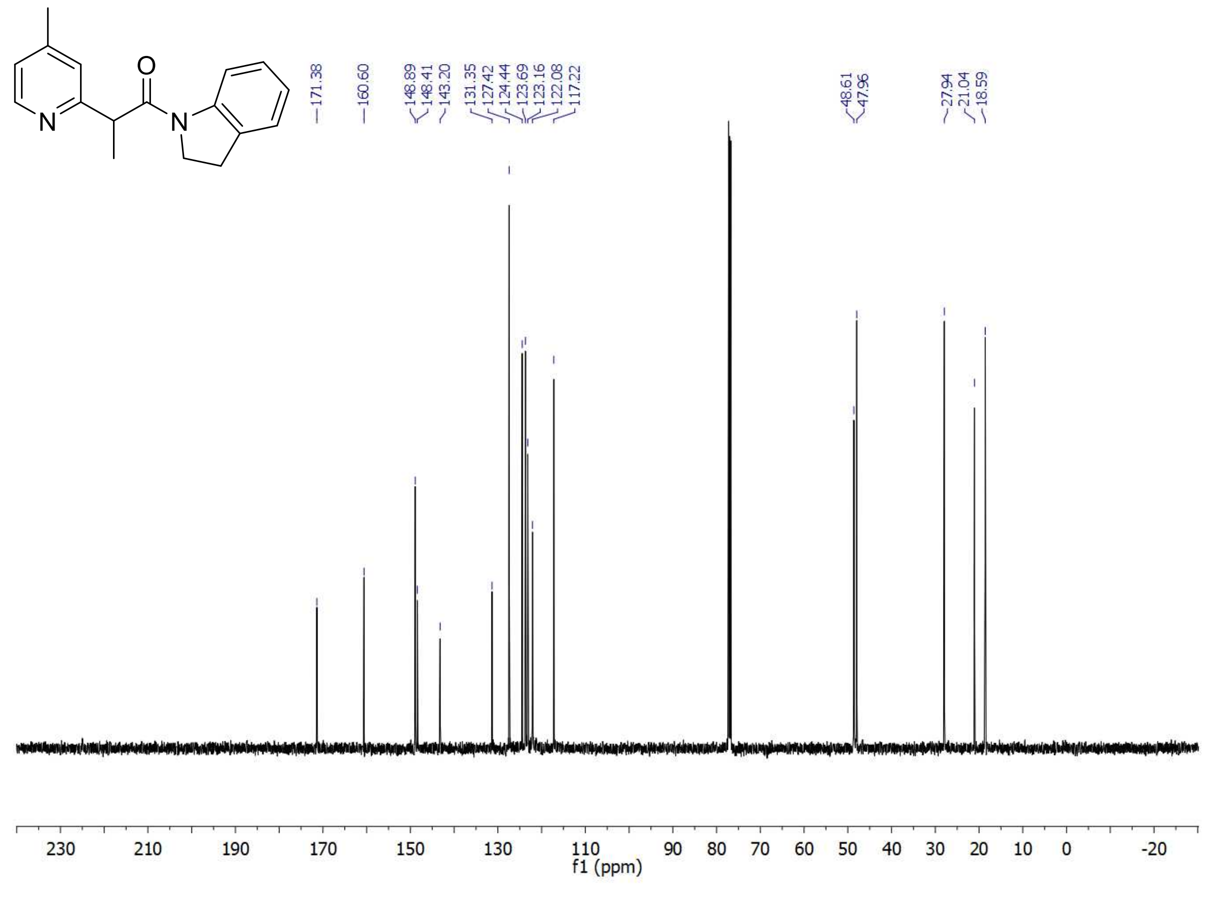

Supplement: Supplementary file 1 — jo2c01597_si_001.pdf [file jo2c01597_si_001.pdf]
